# Splicing the active phases of copper/cobalt-based catalysts achieves high-rate tandem electroreduction of nitrate to ammonia

**DOI:** 10.1038/s41467-022-28728-4

**Published:** 2022-03-02

**Authors:** Wenhui He, Jian Zhang, Stefan Dieckhöfer, Swapnil Varhade, Ann Cathrin Brix, Anna Lielpetere, Sabine Seisel, João R. C. Junqueira, Wolfgang Schuhmann

**Affiliations:** Analytical Chemistry—Center for Electrochemical Sciences (CES), Faculty of Chemistry and Biochemistry, Ruhr University Bochum, Universitätsstr. 150, 44780 Bochum, Germany

**Keywords:** Electrocatalysis, Energy, Metal-organic frameworks

## Abstract

Electrocatalytic recycling of waste nitrate (NO_3_^−^) to valuable ammonia (NH_3_) at ambient conditions is a green and appealing alternative to the Haber−Bosch process. However, the reaction requires multi-step electron and proton transfer, making it a grand challenge to drive high-rate NH_3_ synthesis in an energy-efficient way. Herein, we present a design concept of tandem catalysts, which involves coupling intermediate phases of different transition metals, existing at low applied overpotentials, as cooperative active sites that enable cascade NO_3_^−^-to-NH_3_ conversion, in turn avoiding the generally encountered scaling relations. We implement the concept by electrochemical transformation of Cu−Co binary sulfides into potential-dependent core−shell Cu/CuO_x_ and Co/CoO phases. Electrochemical evaluation, kinetic studies, and in−situ Raman spectra reveal that the inner Cu/CuO_x_ phases preferentially catalyze NO_3_^−^ reduction to NO_2_^−^, which is rapidly reduced to NH_3_ at the nearby Co/CoO shell. This unique tandem catalyst system leads to a NO_3_^−^-to-NH_3_ Faradaic efficiency of 93.3 ± 2.1% in a wide range of NO_3_^−^ concentrations at pH 13, a high NH_3_ yield rate of 1.17 mmol cm^−2^ h^−1^ in 0.1 M NO_3_^−^ at −0.175 V vs. RHE, and a half-cell energy efficiency of ~36%, surpassing most previous reports.

## Abstract (web summary)

Electrocatalytic recycling of waste nitrate to NH_3_ under ambient conditions maybe an appealing alternative to the Haber−Bosch process. Here the authors report a tandem catalyst system involving cooperative adsorption of reaction intermediate on different transition metal active sites for nitrate electroreduction with high efficiency.

## Introduction

Ammonia (NH_3_) is the critical feedstocks of artificial fertilizers and various chemicals and one of the most promising carbon-free energy carriers^1–3^. Currently, industrial NH_3_ synthesis heavily relies on the energy and carbon−emission intensive Haber−Bosch (H–B) process^4–7^. Alternatively, electrocatalytic N_2_-to-NH_3_ conversion (*e*N_2_–NH_3_), using water (H_2_O) as a proton source, has recently attracted significant research interests owing to its mild conditions and high compatibility with renewable electricity^6,8–15^. However, the inherent characters of N_2_, including high dissociation energy of the N≡N bond (945 kJ mol^−1^) and low water solubility, make the *e*N_2_–NH_3_ work at an insufficient selectivity and two orders of magnitude lower yield rate than that of H–B process^9–11,16^. To bridge the gap, the knowledge of the nitrogen cycle brings a renewed attention to the recycling of reactive N–containing species (e.g., NO and nitrate) to NH_3_^17–20^. Among them, the nitrate (NO_3_^−^) anion is particularly attractive because it exhibits comparatively low dissociation energy of the N=O bond (204 kJ mol^−1^) and is widely abundant as pollution in agricultural and industrial wastewaters^20–26^. Furthermore, initial developments of plasma techniques promise to convert air to NO_3_^−^ with low energy consumption^27,28^. Therefore, using NO_3_^−^ as the precursor endows NH_3_ electrosynthesis with sustainable features and opens up an economical route to remedy environmental pollution.

NO_3_^−^-to-NH_3_ conversion in microorganisms is a tandem process; i.e., NO_3_^−^ reduction to NO_2_^−^ using nitrate reductase and subsequent NO_2_^−^-to-NH_3_ conversion employing nitrite reductase or nitrogenase, independently^29–31^. This enzyme-based tandem system allows efficient NH_3_ generation at ambient conditions in nature owing to its specific coordination binding with NO_3_^−^ and NO_2_^−^, respectively^29,31,32^. However, NO_3_^−^ typically shows low binding affinity to transition metals in aqueous electrolytes due to its symmetrical (D_3h_) resonant structure and strong hydrogen bonding to H_2_O^31,33,34^. Moreover, the NO_3_^−^-to-NH_3_ pathway involves a complex eight−electrons transfer and multiple intermediates^17,35,36^. As a result, there is a scaling relation between the binding strengths of NO_3_^−^, NO_2_^−^ and other oxygen−containing intermediates (e.g., NO) on the transition metal surface^37^. Optimizing the adsorption of one species will typically take the others away from their optima^38,39^, making the simultaneous acceleration of sequential NO_3_^−^-to-NO_2_^−^ and NO_2_^−^-to-NH_3_ reactions considerably challenging.

Copper (Cu)-based catalysts have been intensively investigated for the NO_3_^−^ reduction reaction (NO_3_RR) due to its favorable ability to bind NO_3_^−^ and catalyze NO_3_^−^-to-NO_2_^−^ conversion^40–43^. However, pure Cu catalysts commonly suffer from rapid deactivation because of their strong adsorption of the NO_3_RR intermediates (e.g., NO_2_^−^ and NO)^42–44^. Substantial efforts have recently been made to alleviate these limitations by regulating the proton- and/or electron-transfer, as well as the binding strengths of partially reduced intermediates adsorbed on Cu centres. This was performed either by alloying Cu with noble or other transition metals (e.g., Pt, Pd and Ni)^40,45–49^ or through the formation of hybrids with molecular solids or metal oxides (e.g., Cu_2_O)^44,50,51^. These strategies have increased the Faradaic efficiency (FE) of NH_3_ to an impressive 70–100% and the NH_3_ yield rate (Y_NH3_) to a level of 30–200 μmol cm^−2^ h^−1^ ^44,45,51^. However, restricted by the scaling relations, these advances require highly concentrated NO_3_^−^ (e.g., 1 M) and/or relatively high overpotentials (<−0.4 V vs. RHE) to balance the rates of NO_3_^−^-to-NO_2_^−^ and NO_2_^−^-to-NH_3_ reactions, resulting in an increased energy consumption^44,45,51^. To compete with the H–B process, further progress on Y_NH3_ (>1 mmol cm^−2^ h^−1^)^52^ and on decreasing energy consumption are highly desirable.

Energy-efficient NO_3_RR points to low operating overpotentials (e.g., >−0.2 V vs. RHE), at which transition metal (e.g., Cu and Co)-based catalysts often suffer from potential−dependent phase evolution, leading to the coexistence of multiple phases, such as metallic, oxide and hydroxide phases^53–56^. In situ monitoring of the phase evolution of transition metals during the NO_3_RR, while correlating these intermediate phases with specific catalytic steps^53,54,57,58^, may not only guide the rational design of selective catalysts for NH_3_ but also provide insight into the NO_3_RR. Inspired by the tandem NO_3_^−^-to-NH_3_ conversion in nature^29^, we sought, therefore, to circumvent the scaling relations by combining two or more cooperative intermediate phases exhibiting complementary catalytic selectivity into one tandem system, intending to achieve NH_3_ synthesis at low overpotentials. Importantly, it has been clearly demonstrated that the reaction rate and selectivity of a tandem catalyst system, linked to the transport of key intermediates, could be optimized by judiciously tuning the proximity, hierarchy and content ratio of multiple active phases^39,59–62^. Accordingly, the Y_NH3_ could be further improved, however, to the best of our knowledge, tandem catalysts based on earth−abundant elements have never been reported for electrocatalytic consecutive NO_3_^–^-to-NH_3_ conversion.

In this work, we introduce a facile electrochemically driven phase-separation strategy for a tandem catalyst design, which, different from previous approaches based on sequential assembling or deposition^60,61^, enables the in situ formation of multiple active intermediate phases and rich phase interfaces for rapid spillover and transport of reaction intermediates. As a proof-of-concept, we implemented this catalyst synthesis strategy by the electrochemical transformation of pre−synthesized Cu–Co binary metal sulfides into core–shell Cu/CuO_*x*_ and Co/CoO phases on Cu foil. The employment of Co-based phases as a sub−component of the tandem catalysts was inspired by the previously reported high selectivity of Co-based materials and complexes for NH_3_ generation during the NO_3_RR and the specific NO_2_^−^-to-NH_3_ conversion^63,64^. Our electrocatalytic tests, kinetic studies, in situ scanning electrochemical microscopy (SECM) and in situ Raman spectra reveal that at low overpotentials, the inner Cu/CuO_x_ phases preferentially catalyze NO_3_^−^ reduction to NO_2_^−^, while the outer-layer Co/CoO phases selectively catalyze NO_2_^−^ reduction to NH_3_, both of which can be combined for rapid “working-in-tandem” NH_3_ synthesis. As a result, we report NO_3_^−^-to-NH_3_ conversion with a FE of 93.3 ± 2.1% in a wide range of NO_3_^−^ concentrations at −0.175 V vs. RHE, a high NH_3_ yield rate of 1.17 mmol cm^−2^ h^−1^ in 0.1 M NO_3_^−^ at pH 13 and a half−cell energy efficiency of ~36%, which surpass most prevreports.

## Results

### Catalyst design and characterization

**Fig. 1:**
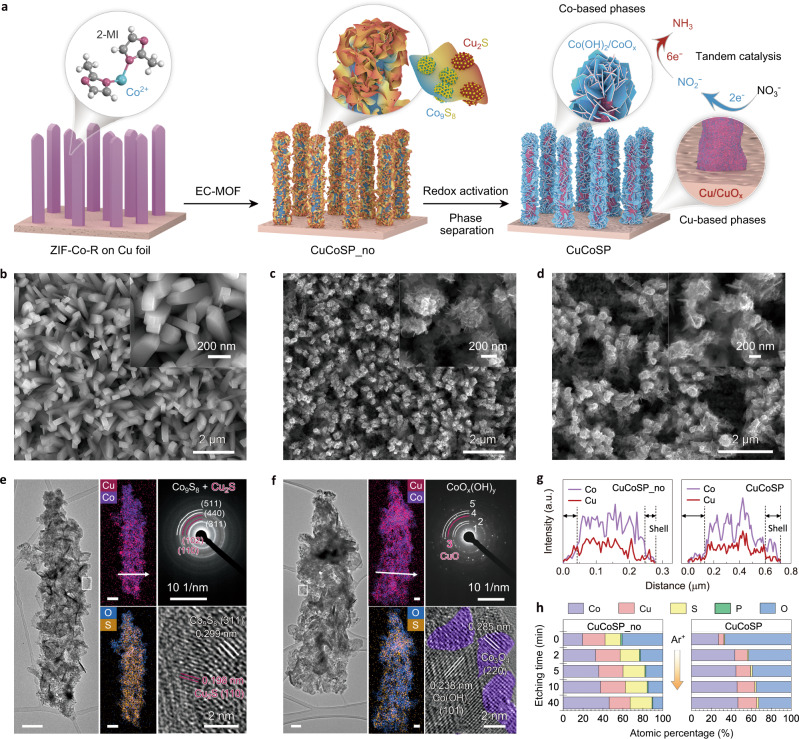
Structural characterizations of catalysts. **a** Schematic illustration of the preparation of a Cu/Co-based binary ‘tandem catalyst’. SEM images of the ZIF-Co-R precursor (**b**), CuCoSP_no (**c**) and CuCoSP (**d**) on the Cu foil substrate. The inset figures are the SEM images at higher magnification. **e**, **f** Typical TEM image, EDX mappings, SAED patterns and HR-TEM image of CuCoSP_no (**e**) and CuCoSP (**f**). Unlabelled scale bars are 100 nm. The SAED patterns in (**f**) show the presence of CuO phases and Co-based oxides/hydroxides (CoO_*x*_(OH)_*y*_), where 1 = Co(OH)_2_ (100), 2 = Co(OH)_2_ (101), 3 = CuO (20$\bar{2}$), 4 = CoOOH (211) and 5 = CoO (220). The purple colours of the HR-TEM image in (**f**) are marked to guide the eye and correspond to the Co_3_O_4_ nanocrystals. **g** EDX line−scan of a selected area marked by the white arrows in (**e**) and (**f**). **h** XPS-determined atomic percentage-depth profile of CuCoSP_no and CuCoSP as a function of Ar^+^ etching time. 2-MI 2-methylimidazole; EC-MOF electrochemically conversion of metal-organic frameworks.

The synthesis of Cu/Co-based tandem catalysts is schematically illustrated in Fig. 1a. A nanorod array of metal-organic frameworks (MOFs) was first grown on Cu foil by optimizing the molar ratio of 2-methylimidazole and Co^2+^ to 20: 1 (hereafter named as ZIF-Co-R/Cu; Fig. 1b and Supplementary Fig. S1). The ZIF-Co-R/Cu was converted into Cu–Co binary metal sulfides following a previously reported electrochemically conversion of MOF (EC-MOF) strategy^65–67^. Upon adding 0.05 M Na_2_HPO_4_ as structure-tuning agents, the nanorod contour of ZIF–Co−R could be retained during the EC-MOF (Fig. 1c), which was otherwise changed into super-thin nanosheets (Supplementary Fig. S2). We denoted the PO_4_^3−^-modified Cu–Co binary metal sulfides as CuCoSP_no and their products after further electrochemical redox activation as CuCoSP. This unique nanorod array configuration of CuCoSP can provide a more efficient pathway for mass and charge transport during the NO_3_RR, contributing to the best performance of CuCoSP for NO_3_RR as discussed below.

Low-resolution transmission electron microscopy (TEM) and high-resolution scanning electron microscopy (HR-SEM) images reveal that a curly nanosheet-assembled shell wraps the nanorod skeleton of CuCoSP_no (Fig. 1c, e and Supplementary Fig. S3a). The corresponding EDX mapping shows strong Co, Cu and S signals well-distributed in the whole nanorod and a weak signal of O due to surface oxidation. Remarkably, Cu is also spread over the nanorod, indicating that the Cu comprised in CuCoSP_no is from the dissolution of Cu foil followed by redeposition and inward diffusion of the released Cu ions during the EC-MOF. The enrichment of Cu on the CuCoSP_no surface is supported by the EDX-linear scan (Fig. 1g and Supplementary Fig. S3). Moreover, HR-TEM images, coupled with selected area electron diffraction (SAED), reveal that the curly nanosheets and skeleton of CuCoSP_no are mainly composed of ~3–5 nm Co_9_S_8_ and Cu_2_S nanocrystals, which are not detectable by X-ray diffraction (XRD) (Supplementary Fig. S4d)^59,66^.

After the electrochemical activation, the obtained CuCoSP preserves the contour of CuCoSP_no, except for the in situ formation of a hexagon-shaped nanosheet-assembled shell (Fig. 1d, f and Supplementary Fig. S5a). Compared to CuCoSP_no, the CuCoSP possesses more complex SAED patterns corresponding to multiple mixed phases (Fig. 1f), which include Co(OH)_2_, CoOOH, CoO and CuO EDX mappings and linear EDX scans (Fig. 1f and Supplementary Fig. S5f) show weaker S and enhanced O signals. These results suggest a significant transformation of Cu–Co binary sulfides into oxides and hydroxides, as corroborated by the XRD patterns (Supplementary Fig. S4e), as well as Raman and X-ray photoelectron spectroscopy (XPS). An HR-TEM image of a hexagon-shaped nanosheet on CuCoSP shows a typical (101) facet of Co(OH)_2_ and some Co_3_O_4_ nanocrystals at the edge. The EDX mapping of CuCoSP shows an intertwined distribution of Cu and Co on the nanorod (Fig. 1f), and the linear EDX scan reveals a higher content of Co in the shell region (Fig. 1g). Therefore, the shell assembled by xagon-shaped nanosheets is a Co element-rich phase, and the redox activation might induce the redistribution of Co-based and Cu-based phases.

To investigate the spatial arrangement of Cu-based and Co-based phases, we carried out XPS depth profiling. We calculated the atomic percentages of Cu, Co, O, S and P in both CuCoSP_no and CuCoSP as a function of Ar^+^ etching time (Fig. 1h and Supplementary Fig. S6). We found that the CuCoSP_no has a Cu-rich surface (Co: Cu = 0.862: 1) and Co-rich core (Co: Cu = 1.83: 1 at 40 min etching time), which is in line with the EDX-linear scan results. The surface of CuCoSP is enriched with Co with a Co/Cu ratio of 5.23: 1. After 5 min etching time, the Co/Cu ratio decreases to 3.07: 1 and slightly decreases in the following 35 min of Ar^+^ etching. The results reveal an electrochemical redox activation-induced outward diffusion of Co ions and a relative inward diffusion of Cu ions, contributing to the phase separation in CuCoSP. The outward diffusion of Co ions is attributed to the easier oxidation of cobalt sulfides than copper sulfides into corresponding oxides or hydroxides. EDX mapping images of CuCoSP_no after one-cycle redox activation provide further evidence (Supplementary Fig. S7), with the maps of Co and O overlapping on the outer-shell layer and those of Cu and residual S in the inner-core layer. This controllable phase separation in metal sulfide matrix driven by electrochemically redox activation may offer a strategy for a tandem catalyst design, which enables in situ formation of multiple active phases and rich phase interfaces for rapid spillover and transfer of reaction intermediates.

This core–shell arrangement of the Cu-based phases and Co-based phases, together with the rich phase interface between them, is the key to achieve a near-unity selectivity for subsequent NO_3_^−^-to-NH_3_ catalysis on CuCoSP. The Cu foil substrate of CuCoSP may play a similar role to the Cu-based phases (Fig. 1a). As a control, we used a Cu foil or ZIF-Co-R grown on carbon paper (CC) as precursors and treated them by the same EC-MOF and activation procedures (Supplementary Fig. S8). The related materials were denoted as CuSP and CoSP, respectively.

### NO_3_RR performance

**Fig. 2:**
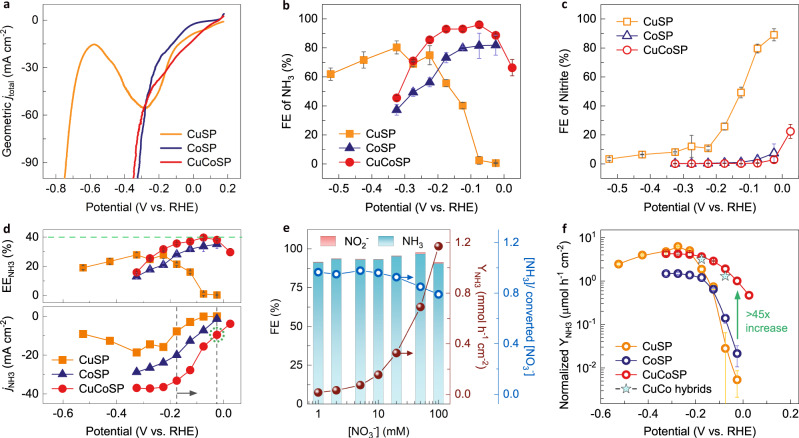
NO_3_^−^-to-NH_3_ conversion performance. LSVs at a scan rate of 5 mV s^−1^ (**a**), Faradaic efficiencies (FE) for NH_3_ (**b**) and NO_2_^−^ (**c**) on CuSP, CoSP and CuCoSP in 0.01 M NO_3_^−^ and 0.1 M KOH (pH 13) at various applied potentials. **d** A comparison of half-cell energy efficiencies of NH_3_ (EE_NH3_) and partial current densities of NH_3_ (*j*_NH3_) on CuSP, CoSP and CuCoSP catalysts at various potentials. **e** The FE of NO_2_^−^ and NH_3_, the NH_3_ yield rate (Y_NH3_), as well as the ratio of the formed NH_3_ concentration [NH_3_] to the converted NO_3_^−^ concentration [NO_3_^−^] on the CuCoSP catalyst at −0.175 V vs. RHE at [NO_3_^−^] in the range of 1−100 mM at pH 13. **f** The ECSA-normalized Y_NH3_ on CuSP, CoSP, CuCoSP and metallic CuCo hybrids in 0.01 M NO_3_^−^ and 0.1 M KOH at various potentials. Error bars denote the standard deviations calculated from three independent measurements.

Linear sweep voltammetry (LSV) and one-hour electrolysis were performed for assessing the activity and selectivity of the as-synthesized catalysts in 0.01 M KNO_3_ and 0.1 M KOH (pH 13) (Fig. 2a–c). Unless otherwise noted, all potentials are corrected vs the reversible hydrogen electrode (RHE). Performing NO_3_RR in alkaline media was chosen due to the reported lower probability of the formation of toxic intermediates in solution (e.g., nitrogen oxides and NH_2_OH), as compared to those in acidic or neutral media, as well as the need of removing NO_3_^−^ ions in alkaline nuclear wastewater^21,42^. We set the concentration of nitrate to be 0.01 M, which ensures the concentration of formed NH_3_ to be easily higher than that of environmental contaminants which in turn helps to compare the intrinsic activity of the catalysts via bypassing the positive effects of high-concentration NO_3_^−^ (e.g., 1 M) with respect to facilitating the NO_3_RR^52,68^.

The LSV of CuSP in the presence of NO_3_^−^ is similar to that of the NO_3_RR on the Cu (100) facet^42^, where a diffusion-limited maximum total current density (*j*_total_) of −55.3 mA/cm^−2^ is reached at around −0.3 V. At <−0.3 V, the surface of CuSP becomes blocked by the strongly adsorbed intermediates of the H_2_ evolution reaction (HER) and/or NO_3_RR until the surface is renewed because of the drastic HER at <−0.6 V^42^. CoSP requires a ~150 mV more negative potential to drive the NO_3_RR, but it shows a sharply increased *j*_total_ for the HER at <−0.2 V, where metallic Co is supposedly formed (Supplementary Fig. S9). The CuCoSP has the catalytic features of both CuSP and CoSP, showing the highest NO_3_RR activity between −0.16 V and 0.085 V. Moreover, the Tafel slope of CuCoSP for the HER in 0.1 M KOH is 69 mV dec^−1^, and smaller than those of CuSP (125 mV dec^−1^) and CoSP (104 mV dec^−1^), suggesting faster kinetics for the HER (Supplementary Fig. S9). Thus, there is a strong synergy between the Cu-based and Co-based phases in CuCoSP for NO_3_RR at >−0.2 V and HER at <−0.2 V.

Determination of the product selectivity for CuSP, CoSP and CuCoSP shows a significant difference in the FE for NH_3_ and NO_2_^−^ (Fig. 2b, c and Supplementary Figs. S10–13). At low over−potentials (>−0.2 V), CuSP exclusively catalyzes the formation of NO_2_^−^, while CoSP shows a high inherent NH_3_ selectivity without any interference from the underlying CC (Supplementary Fig. S14d). The CuCoSP inherits the advantages of CoSP and reaches a maximum FE of 95.9% for NH_3_ at −0.075 V, 14.5% higher than that of CoSP. This finding further suggests the synergy of the Cu-based and Co-based phases in CuCoSP for selective NO_3_^−^-to-NH_3_ conversion. Moreover, the CuCoSP shows a FE of 88.7% for NH_3_ and an NH_3_ partial current densities (*j*_NH3_) of −9.54 mA cm^−2^ at −0.025 V, a potential at which CuSP exhibits exclusive NO_2_^−^ generation and CoSP shows a negligible NO_3_RR activity (Fig. 2a–d and Supplementary Figs. S11–13). CuCoSP retains this advantage even at 0.025 V (a FE of 66.4% for NH_3_ and a *j*_NH3_ of −3.84 mA cm^−2^). Similar to CuSP, the Cu foil and inner Cu-based phases of CuCoSP can catalyze the preferential formation of NO_2_^−^, which might then be further reduced to NH_3_ at the outer Co-based phases. This hypothesis, as further demonstrated below, may well account for the ~2-fold higher *j*_NH3_ for CuCoSP than that of CoSP at >−0.175 V (Fig. 2d).

At higher overpotentials (<−0.2 V), CuCoSP and CoSP show a sharp decline of the FE for NH_3_ but minor changes in their FE for NO_2_^−^ (<1%) (Fig. 2b–d), indicating that the NO_3_RR on CuCoSP and CoSP is challenged by the drastically enhanced HER (Supplementary Fig. S9). The competing HER was previously suppressed by increasing the NO_3_^−^ concentrations and/or the pH value of electrolytes^45,52^. As anticipated, *j*_total_ of CuCoSP linearly increases with the NO_3_^−^ concentrations (Supplementary Fig. S15), indicative of a first-order reaction kinetics. At −0.175 V, the CuCoSP catalyst reaches a nearly equal FE of 93.3 ± 2.1% for NH_3_ and a linearly increased NH_3_ yield rate (Y_NH3_) with a value of 15.7, 33.4, 74.1, 155 and 327 μmol h^−1^ cm^−2^ in 1, 2, 5, 10 and 20 mM NO_3_^−^, respectively (Fig. 2e). When the NO_3_^−^ concentration increases to 50 and 100 mM, the Y_NH3_ of CuCoSP reaches 0.690 and 1.17 mmol h^−1^ cm^−2^, respectively. The latter is comparable to the highest reported value of 1.17 mmol h^−1^ cm^−2^ on strained Ru nanoclusters evaluated at −0.2 V in 1 M NO_3_^−^ and 1 M KOH (pH 14)^52^. The turnover numbers (TON) of nitrate on CuCoSP, defined by the ratio of the yielded NH_3_ concentration [NH_3_] to the converted NO_3_^−^ concentration [NO_3_^−^], are close to 1 at [NO_3_^−^] < 20 mM. This indicates that the formed NH_3_ is mainly derived from the NO_3_^−^ electroreduction rather than any environmental contaminations (Fig. 2e and Supplementary Fig. S15d). However, the TON value decreases to ~0.8 in 100 mM nitrate electrolytes, suggesting that high [NO_3_^−^] might induce the formation of gaseous by-products (e.g., N_2_ and NO_x_)^36^. Finally, the CuCoSP achieves a half-cell energy efficiency of NH_3_ (EE_NH3_) close to 40%. These results endow the proposed CuCoSP ranking among the best NH_3_ synthesizing electrocatalysts (Supplementary Table S1).

To assess the origin of the detected NH_3_ and correct the Y_NH3_, we employed ^1^H NMR to detect the NH_3_ generation on CuCoSP in 0.1 M KOH containing 0.01 M ^15^N-labelled ^15^NO_3_^−^ or ^14^NO_3_^−^ (Supplementary Fig. S16). The ^14^NH_3_ yield quantified by ^1^H NMR is very close to that determined by colorimetric methods^69^, confirming the reliability of our results. The negligible NH_3_ generation in blank 0.1 M KOH and the typical ^1^H NMR double peaks of ^15^NH_4_^+^ after the electrolysis of ^15^NO_3_^−^ suggest that the obtained NH_3_ indeed originates from the NO_3_RR^25,28,44^.

To derive the intrinsic activities of the catalysts, we normalize their Y_NH3_ by the electrochemical active surface area (ECSA) (correlated with the double-layer capacity (C_dl_)) (Fig. 2f and Supplementary Fig. S17). At low overpotentials, the Y_NH3_ of CuCoSP can be ~45 times and two orders of magnitude higher than those of CoSP and CuSP, respectively, confirming the high intrinsic performance of CuCoSP. Remarkably, CuSP lost activity rapidly at >−0.525 V during the NO_3_^−^ electrolysis (Supplementary Fig. S11), which occurred neither at CoSP nor at CuCoSP (Supplementary Fig. S12 and S13). This finding indicates that the Co-based phases existing in CoSP and CuCoSP may help to avoid similar poisoning effects. When the NO_3_RR was performed at −0.175 V for 10 h over the CuCoSP catalyst, no appreciable decay in activity and selectivity for NO_3_^−^-to-NH_3_ conversion was observed, and the structural features of CuCoSP were retained (Supplementary Fig. S18).

### Understanding the high-rate NH_3_ generation on CuCoSP

**Fig. 3:**
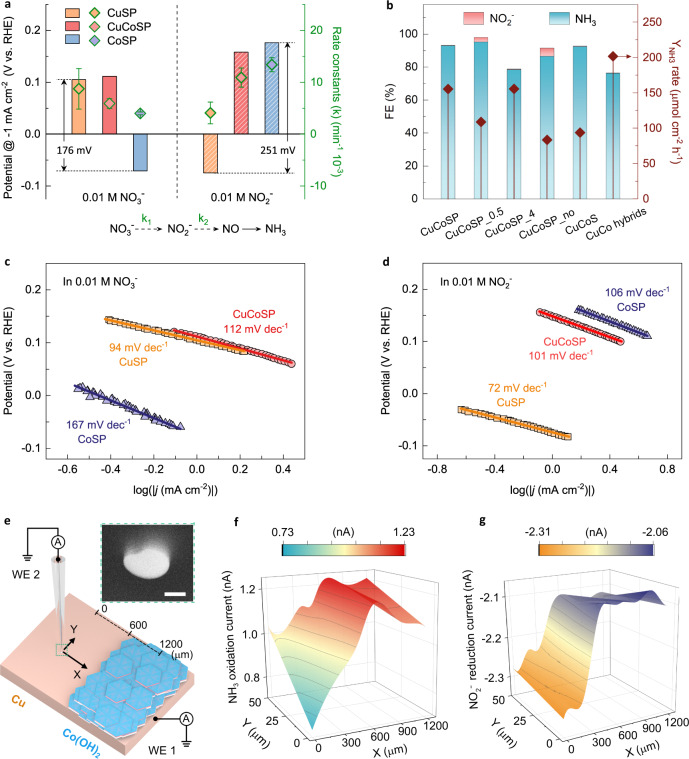
Evaluation of the reaction kinetics and mechanisms of the catalysis of the NO_3_RR. **a** The LSV-derived potentials at a current density of −1 mA cm^−2^ and the calculated reaction constants (k, k_1_ for NO_3_^−^-to-NO_2_^−^ and k_2_ for NO_2_^−^-to-NO conversion) for NO_3_^−^ and NO_2_^−^ reduction on CuSP, CoSP and CuCoSP catalysts. k was calculated based on the concentration evolution of NO_3_^−^ or NO_2_^−^ ions as a function of electrolysis time at −0.175 V vs. RHE in 0.01 M NO_3_^−^ and NO_2_^−^ at pH 13. Error bars denote the standard deviations of k calculated at different time points during 1 h electrolysis. **b** A comparison of the FE and the Y_NH3_ on the control catalysts: CuCoSP_0.5, CuCoSP_4, CuCoSP_no, CuCoS and metallic CuCo hybrids. The LSV-derived Tafel slopes of CuSP, CoSP and CuCoSP in 0.01 M nitrate (**c**) and nitrite (**d**) at pH 13, respectively. The LSVs were recorded at a scan rate of 1 mV s^−1^. **e** Schematic representation of the SECM setup that is operated in a sample generation-tip collection (SG-TC) mode using a Pt-UME (WE 2) to detect NO_2_^−^ and NH_3_ produced during NO_3_RR on Cu_Co(OH)_2_ model catalyst (WE 1). Inset: SEM image of Pt-UME tip; scale bar is 500 nm. **f**, **g** Current maps of NH_3_ oxidation (**f**) and NO_2_^−^ reduction (**g**) recorded at 0.76 V and 0.06 V (vs. RHE) at the Pt-UME, respectively, when a potential of −0.12 V (vs. RHE) is applied to the Cu_Co(OH)_2_ model catalyst in 50 mM NO_3_^−^ at pH 13.

Electrocatalytic NO_3_RR follows a consecutive pathway (Fig. 3a), where NO_2_^−^ is generated as a stable intermediate^17,36^. A fast NO_3_^−^-to-NH_3_ conversion requires the simultaneous acceleration of the sequential NO_3_^−^-to-NO_2_^−^ and NO_2_^−^-to-NH_3_ reactions^35,36^. To rationalize the high Y_NH3_ on CuCoSP at low overpotentials, we firstly compared the potentials of the three catalysts required to reach −1 mA cm^−2^ (kinetic area with negligible mass transport limitation) in 0.01 M NO_3_^−^ and NO_2_^−^, respectively (Supplementary Fig. S20). CuSP shows a 176 mV more positive potential than CoSP for NO_3_^−^ reduction, while CoSP has a 251 mV more positive potential than CuSP for NO_2_^−^ reduction. Therefore, CuSP and CoSP may play a complementary role for the consecutive NO_3_RR. Significantly, CuCoSP combines the positive properties of CuSP and CoSP for NO_3_^−^ and NO_2_^−^ reduction, respectively, implying that there are likely two types of active phases in CuCoSP: Cu-based phases which are similar to those in CuSP, and Co-based phases which are similar to those in CoSP. The two types of active phases in CuCoSP synergistically catalyze the tandem NO_3_RR.

To validate this hypothesis, we further evaluated the rate constants k_1_ and k_2_ of each catalyst for NO_3_^−^-to-NO_2_^−^ and NO_2_^−^-to-NH_3_ conversion, respectively (Supplementary Fig. S21 and Table S2). We found that the rate constants of the three catalysts follow a similar trend as their potentials at −1 mA cm^−2^; a larger rate constant corresponding to a more positive potential for reaching −1 mA cm^−2^ (Fig. 3a). The largest k_1_ but the smallest k_2_ values of CuSP correspond to a fast reduction of NO_3_^−^ to NO_2_^−^ and subsequent desorption of NO_2_^−^ to the electrolyte, resulting in the observed high FE of NO_2_^−^ (Fig. 2c and Supplementary Fig. S11). On the CuCoSP surface, NO_2_^−^ intermediates are preferentially formed on Cu-based phases and then spilt over to nearby Co-based phases. Compared to the k_2_/k_1_ ratio of CuSP (0.468), the larger k_2_/k_1_ ratios of CoSP (3.14) and CuCoSP (1.78) suggest a fast reduction of the intermediate NO_2_^−^ to NH_3_ over their Co-based phases, thus well explaining their near-unity selectivity for NH_3_ generation. We used the k_1_ values of CoSP and CuCoSP to estimate their local surface NO_2_^−^ concentration during the NO_3_RR and find a 1.54-fold higher value on CuCoSP than on CoSP (Supplementary Table S2), which is consistent with the observed 1.66-fold larger *j*_NH3_ of CuCoSP compared to CoSP at −0.175 V (Fig. 2d). This finding unveils the crucial role of Cu-based phases in CuCoSP for creating a high local NO_2_^−^ concentration, which accelerates subsequent NH_3_ generation on its Co-based phases, ultimately contributing to the tandem catalysis of NO_3_RR.

Considering the lowest k_1_ value of CoSP, the smaller k_1_ value of CuCoSP than CuSP can be rationalized since the less active Co-based phases of CuCoSP may block its active Cu-based phases (especially the Cu foil substrate) for NO_3_^−^ reduction. As such, we synthesized the CuCoSP_0.5 and CuCoSP_4 with 0.5 h and 4 h growth of ZIF-Co-R on Cu foil, respectively. The CuCoSP_0.5 shows catalytic features close to CuSP, thus giving a higher FE for NO_2_^−^ than CuCoSP (Fig. 3b and Supplementary Fig. S22). This finding indicates that possible Co doping in Cu-based phases of CuCoSP_0.5 does not change their catalytic nature. Likewise, the CuCoSP _4 shows a similar catalytic feature to CoSP with a low sum FE for NO_3_RR. Therefore, the content ratio of Cu-based and Co-based phases in CuCoSP, rather than the Cu or Co doping, determines the rates of the NO_3_^−^-to-NO_2_^−^ and NO_2_^−^-to-NH_3_ reactions. This is a typical feature of tandem catalysts^39,59^.

Electrokinetic analysis was conducted to determine the rate-determining step (RDS) of the NO_3_^−^ and NO_2_^−^ reduction catalyzed by the three catalysts. In 0.01 M NO_3_^−^ (Fig. 3c), CuSP and CuCoSP show Tafel slopes of 94 and 112 mV dec^−1^, respectively, a little lower than 120 mV dec^−1^, suggesting that the RDS is the first one-electron transfer occurring during the NO_3_^−^-to-NO_2_^−^ conversion^34,48,70^. The much higher Tafel slope of CoSP (167 mV dec^−1^) indicates that the NO_3_RR over CoSP is limited by the initial adsorption and activation of NO_3_^−^^48^. In 0.01 M NO_2_^−^ (Fig. 3d), CoSP and CuCoSP show Tafel slopes of 106 and 101 mV dec^−1^, respectively, suggesting that the RDS is the first one-electron transfer for the reduction of NO_2_^−^ to NO^34,48^. CuSP has a Tafel slope of 72 mV dec^−1^, close to the critical value of 60 mV dec^−1^, implying that the RDS is a chemical step^71,72^. Based on the theoretical NO_3_RR pathways on Cu^44^, this RDS is likely the coupling of strongly adsorbed *NO and *H. Remarkably, the smaller Tafel slope of CuSP than those of CoSP and CuCoSP points to a faster NO_2_^−^ reduction kinetics on CuSP, but CuSP suffers from a faster deactivation in 0.01 M NO_2_^−^ than in 0.01 M NO_3_^−^ (Supplementary Fig. S21), thus giving the smallest apparent k_2_ value. Accordingly, the observed poisoning of CuSP might be ascribed to the strongly adsorbed *NO species. Therefore, CuCoSP combines the nature of CuSP for NO_3_^−^ reduction with that of CoSP for NO_2_^−^ reduction.

To assess the possible roles of anionic ligands (PO_4_^3−^ and S^2−^) in our system, we tested the NO_3_RR performance of CuCoSP_no catalysts with the maximum PO_4_^3−^ and S^2−^ ligands and CuCoS catalysts with only S^2−^ ligand. The lower FE (for NH_3_) and Y_NH3_ of CuCoSP_no compared with those of CuCoSP (Fig. 3b), in combination with the near-unity NH_3_ selectivity but low Y_NH3_ of CuCoS (Fig. 3b and Supplementary Fig. S23), suggest that PO_4_^3−^ and S^2−^ ligands most likely do not play a prominent role. We evaluated the final morphology of CuCoSP after repeating three electrolysis of one hour at −0.325 V and observed a separation of Cu-based phases (nanorods) and Co-based phases (hexagonal nanosheets) based on EDX mapping and HR-TEM images (Supplementary Fig. S24). This result rules out the formation of a bulk CuCo alloy, as corroborated by the XRD patterns (Supplementary Fig. S4f). To identify the impact of surface CuCo metallizing and/or alloying possibly during the NO_3_RR, we electrodeposited a hybrid of Cu–Co metals and alloy (Co: Cu = 2.82: 1, close to that on CuCoSP surface) on CC (Supplementary Fig. S25). The metallic CuCo hybrids show Tafel slopes of 61 mV dec^−1^ in 0.01 M NO_3_^−^ and 79 mV dec^−1^ in 0.01 M NO_2_^−^, indicating a significantly different catalytic mechanism and kinetics than CuCoSP. Despite its higher apparent Y_NH3_, the CuCo hybrids have a lower FE for NH_3_ and ECSA-normalized Y_NH3_ than CuCoSP (Fig. 2f and 3b). The ECSA-normalized Y_NH3_ of CuCo hybrids is about 85 and 67% of that formed on CuCoSP at −0.175 V and −0.025 V, respectively. As such, the excellent intrinsic performance of CuCoSP towards NH_3_ generation, especially at low overpotentials, is unlikely from surface Cu–Co metallizing or alloying. It can be concluded that the high-rate NO_3_^−^-to-NH_3_ conversion on CuCoSP is due to the coexistence of the complementary Cu-based and Co-based phases: the former catalyzes NO_3_^−^-to-NO_2_^−^ reduction and the latter catalyzes the NO_2_^−^-to-NH_3_ conversion, contributing to the tandem catalysis of NO_3_RR.

To offer a direct evidence for this tandem catalysis of NO_3_RR, we performed SECM experiments in a surface-generation tip-collection mode, which involves using a positioned Pt ultramicroelectrode (Pt-UME) to detect the NO_2_^−^ and NH_3_ formed on the surface of a Cu_Co(OH)_2_ model catalyst (Fig. 3e) during the NO_3_^−^ electrolysis. This Cu_Co(OH)_2_ model catalyst has two adjacent layers to simulate the CuSP (Cu layer) and CoSP (Co(OH)_2_ layer) catalysts, while the border between the two layers may play a similar role of as the adjacent phases in CuCoSP (Supplementary Fig. S26). During the SECM measurements, the catalyst layer was polarized to −0.12 V (vs. RHE), while at the positioned Pt-UME cyclic voltammetry (CV) in the potential range between −0.12 V and 1.58 V (vs. RHE) at a rate of 200 mV s^−1^ was performed. We were applying −0.12 V at catalysts as we found that at this potential, CuSP and CoSP is relatively more stable and active compared to potentials of −0.075 V and −0.025 V, which is beneficial for in situ detection of the intermediately formed NO_2_^−^ and NH_3_. The same reason is the use of a concentration of 50 mM NO_3_^−^. At −0.12 V, CuSP mainly produces NO_2_^−^, and CoSP exhibits a much lower NO_3_RR activity compared to CuCoSP, as indicated in Fig. 2a–c and Supplementary Fig. S11–13.

We firstly performed cyclic voltammograms at the Pt-UME separately in 10 mM NO_3_^−^, NO_2_^−^ and NH_4_Cl at pH 13, which are compared with those in pure 0.1 M KOH (Supplementary Fig. S27a–c). The results indicate that the Pt-UME does not exhibit any activity for the NO_3_RR, but it can efficiently catalyze NO_2_^−^ reduction and NH_3_ oxidation in alkaline media, which is well consistent with previous studies^73–75^. This enables selectively detecting the in situ generated NO_2_^−^ (at 0.06 V) and NH_3_ (at 0.76 V) (Supplementary Fig. S27d–f), when the tip of Pt-UME approaches the surface of Cu_Co(OH)_2_ layer. Accordingly, a high current from NH_3_ oxidation and NO_2_^−^ reduction corresponds to a high local concentration of NH_3_ and NO_2_^−^, respectively, above the surface of Cu_Co(OH)_2_ catalyst. The SECM array scans (Fig. 3f, g) reveal that the amount of generated NH_3_ is substantially increasing at the border (X ≈ 600 μm) between the Cu and Co(OH)_2_ layers, accompanying by a sharply decreased amount of generated NO_2_^−^. The SECM results unequivocally confirmed that the NO_2_^−^ is preferentially formed on the Cu layer and then diffuses to the near Co(OH)_2_ layer, where the NO_2_^−^ is reduced into NH_3_ according to a typical tandem catalysis process. Note that the formed NH_3_ at the border of the Cu_Co(OH)_2_ layers diffuses to both sides, leading to the formation of a concentration gradient around the border during the course of the SECM measurement.

### Identification of the active phases for NO_3_RR

**Fig. 4:**
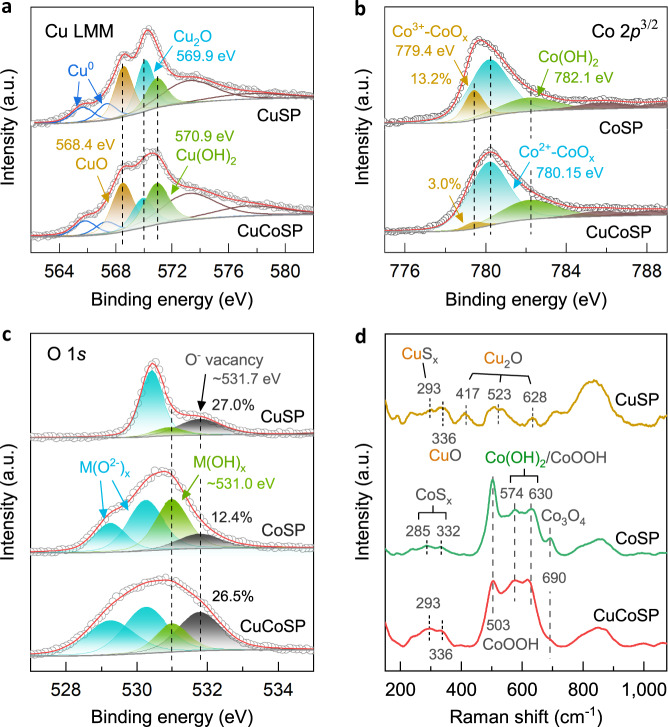
Surface phase compositions of CuSP, CoSP and CuCoSP catalysts. Cu LMM (**a**), Co 2*p*^3/2^ (**b**) and O 1*s* (**c**) XPS spectra. **d** Ex situ Raman spectra.

Ex situ XPS and Raman spectra were obtained to identify the surface phase compositions of the three catalysts (Fig. 4, Supplementary Fig. S28 and Fig. S29). We find that the redox activation induced the transformation of the initial Cu/Co-based sulfide phases into the corresponding oxides and hydroxides (see detailed assignments of the XPS and Raman peaks in Supplementary Notes 1 and 2). As a result, CuSP consists of Cu^0^, CuO, Cu_2_O and Cu(OH)_2_^53,54,76^, whereas CoSP is composed of Co^2+^-dominated CoO and Co(OH)_2_, as well as Co^3+^-containing Co_3_O_4_ and CoOOH^65,77–79^. As anticipated, CuCoSP exists as a combination of CuSP and CoSP in phase compositions, except for the observed suppression of Co^3+^–CoO_x_ phases and increase of Cu(OH)_2_ phases (Fig. 4a, b), which points to the synergy interaction between Cu-based and Co-based phases in CuCoSP. O 1*s* XPS spectra reveal a ~2-fold higher content of O vacancies on CuSP and CuCoSP than on CoSP (Fig. 4c and Supplementary Fig. S28c)^80^. Together with the observed smaller Tafel slopes of CuSP and CuCoSP compared to CoSP (Supplementary Fig. S28f), this result highlights a potential role of O vacancies in Cu-based phases for the initial adsorption and/or activation of free NO_3_^−^ ions^50^, which is a key challenge for most of the reported NO_3_RR catalysts.

**Fig. 5:**
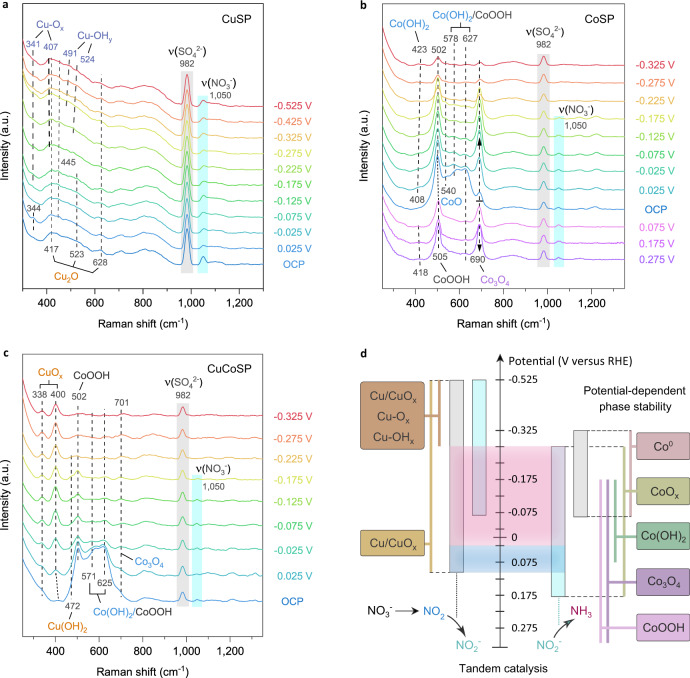
In situ Raman spectra of the catalysts and schematic of the tandem mechanisms of CuCoSP catalysts. In situ Raman spectra of CuSP (**a**), CoSP (**b**) and CuCoSP (**c**) at different applied potentials in electrolytes containing 0.01 M NO_3_^−^, 0.04 M K_2_SO_4_ and 0.01 M KOH. **d** A proposed reaction mechanism of CuCoSP tandem catalysis of NO_3_RR at low overpotentials. In the middle of (**d**), the light blue region corresponds to the potential range for the reduction of NO_3_^−^ to NO_2_ species at the Cu/CuO_*x*_ phases, resulting in the etching of catalysts, while the light pink region shows the potential range for efficient tandem reduction of NO_3_^−^ to NH_3_.

To derive the active phases for NO_3_RR experimentally, we used in situ Raman spectroscopy to monitor the phase evolution of the three catalysts at a series of applied potentials in 0.01 M KOH in the presence or absence of 0.01 M NO_3_^−^ (Fig. 5 and Supplementary Fig. S30). NO_3_^−^ ions exhibit a characteristic peak at ~1050 cm^−1^ ^81^. K_2_SO_4_ was added to ensure sufficient ionic conductivity and provides SO_4_^2−^ ions as an external Raman reference with a typical signal at ~982 cm^−1^ ^53^.

Figure 5a shows the Raman spectra of CuSP at reducing potentials related to NO_3_RR. The initial broad bands at 417, 523 and 628 cm^−1^, associated with Cu_2_O phases^53,54^, persist at as low as −0.525 V. Remarkably, at <−0.175 V, two sets of peaks emerge at 341, 407, 491 and 524 cm^−1^, previously assigned to Cu–O and Cu–OH modes, respectively^53^. The same but more notable signals are observed in the absence of NO_3_^−^ (Supplementary Fig. S30a), partly due to the NO_3_RR delaying the surface phase evolution. It is worth noting that CuSP can prevent poisoning only at <−0.425 V, where the surface evolution of CuSP might promote stable NH_3_ generation. As a control, we further assessed the performance of a metallic Cu foil for NO_3_RR (Supplementary Fig. S14). The results show that at potentials >−0.425 V, Cu foil suffered from deactivation in a much higher rate, compared to CuSP containing mixed Cu/CuO_x_ phases. Moreover, at low overpotentials, the Cu foil mainly electrochemically catalyzes the reduction of NO_3_^−^ to highly oxidative NO_2_ and acts as reducing reagents for the subsequent chemical reduction of NO_2_ to NO_2_^−^, which is supported by the over 100% FE for NO_3_RR at −0.025 and −0.125 V (vs. RHE) (Supplementary Fig. S14c). This chemical step will lead to the partial oxidation of metallic Cu to CuO_*x*_ in alkaline electrolytes. According to recent DFT calculations on Cu and mixed Cu/Cu_2_O phase, the latter was suggested to be more active for NO_3_^−^ to NO_2_^−^ conversion^44^. Therefore, at low overpotentials, the catalytic nature of CuSP for NO_3_RR is related to the Cu/CuO_*x*_ phases, considering the XPS-evidenced presence of Cu^0^ phase.

On the CoSP catalysts, the characteristic Raman peaks of multiple Co^3+^-based phases and Co(OH)_2_ are fast attenuated with decreasing potentials from 0.025 V to −0.325 V (Supplementary Fig. S30b)^77–79^. This indicates the gradual conversion of Co^3+^-based phases and Co(OH)_2_ into CoO and metallic Co in the absence of NO_3_^−^^77^. In 0.01 M NO_3_^−^ the attenuation rate of these Raman peaks was much slower (Fig. 5b). Remarkably, the Raman signals associated with CoOOH and Co_3_O_4_ phases increase at 0.025 and −0.025 V, suggesting the partial oxidation of Co^2+^ in CoSP to Co^3+^^77,78^. Because of this, the electrolysis product of NO_3_^−^ at 0.025 and −0.025 V should be only the highly oxidative NO_2_ species in Ar-saturated alkaline electrolytes^49^, and their formation slows down the phase conversion. In this context, the Co^2+^-based phases act as chemical reducing reagents and offer additional electrons for reduction of NO_2_ to NO_2_^−^, after which the formed NO_2_^−^ will be electrochemically reduced to NH_3_ as evidenced by the low overpotential of CoSP for the NO_2_^−^ reduction (Fig. 3a), finally contributing to the apparently highest FE for NH_3_ at low overpotentials (Fig. 2b). At <−0.025 V, the Raman signals of Co^3+^-based phases and Co(OH)_2_ start to weaken. This result, together with the potential at −1 mA cm^−2^ of CoSP catalysts for NO_3_^−^ reduction (−0.071 V), suggests that the fast reduction of NO_3_^−^ into NO_2_^−^ on CoSP requires the in−situ formation of metallic Co. By contrast, we found that the Co^2+^ in CoSP was oxidized to Co^3+^ already at >0.025 V^77–79^. Since the potential at −1 mA cm^−2^ of CoSP for NO_2_^−^ reduction is limited to 0.176 V (Fig. 3a), it can be concluded that the sharply increased Co^3+^-based phases at potentials >0.025 V are inactive for NO_2_^−^ reduction. Thus, the active phase of CoSP for reducing NO_3_^−^ to NO_2_^−^ is related to metallic Co, while that for NO_2_^−^-to-NH_3_ conversion is a Co^2+^-dominated CoO_*x*_ phase.

For CuCoSP, the Raman peaks associated with Co-based phases were quickly attenuated with decreasing potentials, while a phase assigned to CuO_x_ emerges at 338 and 400 cm^−1^ (Fig. 5c and Supplementary Fig. S30c)^54^. These results suggest an electrochemically driven phase separation in CuCoSP, leading to the formation of hybrid of Cu/CuO_x_ and Co/CoO phases. The CuO_*x*_ persists to potentials as low as −0.325 V, further decreasing the probability of in situ surface CuCo alloying, especially at potentials >−0.2 V. This is further supported by the XRD patterns, HR-TEM and EDX-mapping images of CuCoSP after repeating three electrolysis cycles of one hour at −0.325 V (Supplementary Fig. S4f and Fig. S24). The rate at which these Raman peaks are evolving is almost not impacted by the NO_3_RR (Fig. 5c and Supplementary Fig. S30c). This might be attributed to the high rate of NO_3_RR on CuCoSP, which establishes a depletion layer of NO_3_^−^ explaining the minor effects of the NO_3_RR on the phase-evolving rate. In stark contrast to CoSP (Fig. 5b), the Raman signals of Co^3+^-based phases in CuCoSP were not enhanced at both 0.025 and −0.025 V in 0.01 M NO_3_^−^. This finding indicates fast reduction of NO_3_^−^ to NO_2_^−^ rather than to oxidative NO_2_ species on the Cu-based phases of CuCoSP. Accordingly, the active Co^2+^-based phases of CuCoSP are stabilized by the Cu/CuO_x_ phases, both of which are combined to form a tandem system for cascade NO_3_^−^-to-NH_3_ conversion at low overpotentials (Fig. 5d).

## Discussion

In summary, we present a concept for designing efficient tandem catalysts, which involves the coupling of potential-dependent intermediate phases of transition metals to act as cooperative catalytic sites for cascade NO_3_^−^-to-NH_3_ conversion. This concept was verified using Cu/CuO_*x*_–Co/CoO hybrids with a well-defined spatial arrangement that is achieved by electrochemical redox activation-induced phase reconstruction of Cu/Co-based binary metal sulfides. In this tandem catalysis system, NO_3_^−^ ions are reduced to NO_2_^−^ preferentially on Cu/CuO_x_ phases, while the NO_2_^−^ intermediates are then transferred and selectively converted to NH_3_ on Co/CoO phases. The sequential NO_3_^−^ and NO_2_^−^ reduction on two different adjacent metal/metal oxide phases enables a high-rate NH_3_ generation at low overpotentials. At −0.175 V vs. RHE, the designed CuCoSP catalysts show an excellent FE for NH_3_ (90.6%) and super−high Y_NH3_ of 1.17 mmol cm^−2^ h^−1^ in 0.1 M NO_3_^−^ at pH 13, outperforming most of the NO_3_RR catalysts at the same conditions. Although tandem catalysis was widely employed in heterogeneous systems, this study provides a direct demonstration of efficiently using distinct potential-dependent intermediate phases as tandem catalytic sites. This concept of splicing active phases of transition metals represents a powerful strategy towards designing high-performance, multi-functional electrocatalysts for multi-step chemical reactions, such as e.g. urea electrosynthesis by integrating NO_3_RR with CO_2_ reduction.

## Methods

### Chemicals

Na_2_HPO_4_ (≥99.0 %) and NaClO solution (17 %) were purchased from VWR. Maleic acid (≥99.0 %) was obtained from Riedel-de Haën. All other chemicals and Cu foil (99.98 %) were from Sigma-Aldrich. All chemicals were used without further purification. Carbon cloth was provided by PHYCHEMI.

### Growth of ZIF-Co-R nanorods on Cu foil and carbon cloth

The Cu foil ($3\times 0.5$ cm^2^) was washed with acetone, ethanol and distilled water, finally dried by blotting paper before use. The CC was initially treated with 1 M HCl (≥37%) for 12 h, washed with deionized water and dried in a 70 °C oven. Then, the CC was soaked in an aqueous solution of 2-methylimidazole (0.4 M, 99% purity) for 12 h and dried by blotting paper before use. For the growth Co-ZIF-R nanorods, the Cu foil or CC was immersed into a 14 ml growth solution, which was prepared by adding 10 ml of 2-methylimidazole (0.4 M) into 4 ml aqueous solution of Co(NO_3_)_2_ 6H_2_O (50 mM, ≥98%). The Co−ZIF-R nanorods were grown under static conditions at 25 °C for 105 min. The purple Co-ZIF-R nanorods on Cu foil or CC were washed with deionized water and dried in a 70 °C oven.

### EC-MOF for synthesis of CuCoSP_no

In a typical three-electrode system, the Co-ZIF-R on Cu foil was used as the working electrode, and Ag/AgCl (sat. KCl) and an FTO slide were employed as the reference and counter electrodes, respectively. In Ar-saturated electrolytes (aqueous solution of 0.5 M thiourea (≥99.0%), 0.25 M KCl (≥99.0%) and 0.05 M Na_2_HPO_4_), the ZIF-Co-R was electrochemically converted via continuous CV scanning between −1.76 V and −0.15 V at a series of scan rates for different CV cycles (at 0.2 V s^−1^ for 1200 CV cycles, at 0.1 V s^−1^ for 300 CV cycles, at 0.05 V s^−1^ for 150 CV cycles and finally at 0.02 V s^−1^ for 100 CV cycles) using an Autolab potentiostat. The black products on Cu foil were taken out, rinsed with water and acetone, blow-dried by Ar gas and stored at −21 °C for further characterization. Using the same method, the CuSP_no was prepared from Cu foil directly; the CoSP_no on CC was synthesized using ZIF-Co-R nanorods on CC as precursors; the CuCoS_no on Cu foil was obtained in an electrolyte with no Na_2_HPO_4_. As a control, metallic CuCo hybrids were prepared via electrodeposition of CuCo metals and alloy on CC in 0.05 M H_2_SO_4_ (98%) solution containing 4 mM CuSO_4_ (≥99%) and 16 mM CoSO_4_ (≥99%) at −1.75 V vs. Ag/AgCl for 400 s.

### Electrochemical redox activation of the samples

Using a Gamry interface 1000 workstation, the samples were activated in 0.1 M KOH (≥85%) and 0.01 M potassium nitrate (≥99%) at 60 mA cm^−2^ for 45 s. They then underwent continuous LSV sweeping at a rate of 20 mV s^−1^ from −0.8 V to −1.65 V (vs. Ag/AgCl) until the polarization curves reached a steady state. This activation process was repeated two or three times. Then, the samples were gently washed with water and acetone, and dried under Ar flow for further tests or characterizations. After the redox activation, the samples of CuCoSP_no, CuSP_no, CoSP_no and CuCoS_no were named as CuCoSP, CuSP, CoSP and CuCoS, respectively.

### Material characterization

SEM was performed using a Quanta 3D FEG scanning electron microscope. TEM images, high-angle annular dark-field TEM images, SAED patterns, element mappings and EDX line-scan were carried out on a JEOL-2800 TEM/STEM system using gold grids. XPS was recorded using an AXIS Nova spectrometer (Kratos Analytical) equipped with a monochromatic Al K*α* X-ray source (1487 eV, 15 mA emission current) and an inert ion gas gun for depth-profiling composition analysis as a function of etching time. For the core-level spectra, the binding energies were calibrated based on the C 1*s* feature located at 284.8 eV. The nuclear magnetic resonance (NMR) spectroscopy was performed on a Bruker 400 MHz NMR spectrometer. XRD were obtained using a Bruker D8 Discover X-ray diffractometer with Cu Kα radiation ($\lambda =1.5418{\AA }$).

### Electrochemical NO_3_RR tests

The electrochemical tests were performed using a three-electrode system connected to the Gamry workstation in a typical H-type cell. The H-type cell was separated by a Nafion 117 membrane (Dupont) that was pretreated following reported procedures^82^. The catalysts were used as the working electrode, while Ag/AgCl (3 M KCl) and platinum mesh were used as the reference and counter electrodes, respectively. The electrolytes were Ar-saturated 0.1 M KOH (pH 13) containing different concentrations of NO_3_^−^. The electrochemical cell was maintained in an Ar atmosphere during experiments. The LSV curves were collected at a scan rate of 5 mV s^−1^. All potentials were calibrated to the RHE reference scale using ${E}_{{{{{{\rm{RHE}}}}}}}={E}_{{{{{{\rm{Ag}}}}}}/{{{{{\rm{AgCl}}}}}}}+0.207{{{{{\rm{V}}}}}}+0.0591\times {{{{{\rm{pH}}}}}}$. The current density was normalized to the geometric electrode area (~0.5 cm^2^). Note that the electrode area was 0.2 cm^2^ for tests in 0.05 and 0.1 M nitrate. The solution resistance (*R*_S_) was measured using potentiostatic electrochemical impedance spectroscopy with a frequency range of 0.1 Hz to 200 kHz and an amplitude of 10 mV_pp_. The potentials were compensated by iR_S_-drop from the electrolyte resistance. Potentiostatic measurements were performed for 1 h in 30 ml cathode electrolyte with a stirring rate of 300 rpm, and then the electrolyte was stored at 4 °C (no more than 2 days) before analysis. To assess the performance change of CuCoSP during ten cycles of one-hour electrolysis at −0.175 V (vs. RHE), the electrolyte (30 ml, 0.1 M KOH and 0.01 M nitrate) was collected after each one-hour electrolysis for product analysis and a fresh electrolyte was used for the next cycle of one-hour electrolysis. Note that NH_3_ volatilization in the electrolytes (pH 13) is negligible during the one-hour electrolysis (Supplementary Fig. S19). The C_dl_ was determined by CV scanning in a non-faradaic potential window at different scan rates (10–120 mV s^−1^). The plot of capacitive anode and cathode current differences $[({j}_{{{{{{\rm{a}}}}}}}-{j}_{{{{{{\rm{c}}}}}}})/2]$ at a set potential against the CV scan rates shows a linear relationship, and the slope is C_dl_.

### Kinetic evaluation

The LSVs of the catalysts were recorded at a scan rate of 1 mV s^−1^ in 0.1 M KOH (pH 13) containing 0.01 M NO_3_^−^ or 0.01 M NO_2_^−^ (KNO_2_, ≥96%). To obtain the rate constant, the electrolysis at −0.175 V (vs. RHE) were performed for 1 h in 22 ml electrolyte (0.1 M KOH) containing 0.01 M NO_3_^−^ or 0.01 M NO_2_^−^ in the cathode chamber. The reaction constant (k_1_ for NO_3_^−^ reduction and k_2_ for NO_2_^−^ reduction) was obtained by monitoring the concentration evolution of NO_3_^−^ or NO_2_^−^ ions as a function of electrolysis time, assuming that their concentrations decayed exponentially as per first-order rate, that is, ${C}_{{{{{{\rm{t}}}}}}}={C}_{0}{\exp }(-k\times t)$, where, *C*_0_ is the initial molar concentration of reactant (NO_3_^−^ or NO_2_^−^) and *C*_t_ is the molar concentration of reactant at time *t*.

### Determination of ion concentrations

#### NH_4_^+^ quantification

The produced NH_3_ was quantitatively determined using the indophenol blue method^10,69^. Typically, a certain amount of electrolyte was taken out from the reaction cell and diluted to 2 ml. Then, 2 ml of 1 M NaOH (≥98%) solution containing sodium citrate (≥99%) and salicylic acid (≥99%) (stored at 4 °C) and 1 ml of freshly prepared 0.05 M NaClO was added. The mixed solution was shaken for few seconds. Finally, 0.2 ml of 1 wt.% sodium nitroferricyanide (≥99%) solution (stored at 4 °C) were added for the colour reaction. After keeping at room temperature for 1 h, the resulting solution was measured using an ultraviolet–visible (UV–Vis) spectrophotometer. The absorbance at ~655 nm was used to determine the concentration of NH_3_. In order to quantify the amount of NH_3_, a calibration curve was built using standard NH_4_Cl (≥99.5%) solution in 0.1 M KOH.

#### NO_2_^−^ quantification^44^

A specific colour reagent for NO_2_^−^ quantification was prepared by mixing 0.20 g of N-(1-naphthyl) ethylenediamine dihydrochloride (≥98%), 4.0 g of sulfonamide (≥99%) and 10 ml of phosphoric acid (85 wt.% in H_2_O) (${{{{{\rm{\rho }}}}}}=1.7{{{{{\rm{g}}}}}}/{{{{{\rm{ml}}}}}}$) with 50 ml of deionized water. In a typical colourimetric test, 1 ml HCl (1 M) was firstly added into the 5 ml of diluted post-electrolysis electrolytes, and then 0.1 ml of colour reagent was added and shaken to obtain a uniform solution. The UV–Vis absorbance at 540 nm was recorded after 20 min at room temperature. The amount of NO_2_^−^ was determined using a calibration curve of NaNO_2_ (≥96%) solutions. N_2_H_4_ and NH_2_OH were probably produced during the electroreduction of nitrate. However, their concentrations are expected to be very low and only measurable at intermediate times, owing to their high reactivity in basic media^42,43^. Thus, we mainly focused on analyzing the yields of NH_3_ and NO_2_^−^.

#### NO_3_^−^ quantification^44,83^

A certain amount of post-electrolysis electrolytes was diluted to 4 ml. Then, 1 ml of 1 M HCl and 0.1 ml sulfamic acid (98%) solution (0.8 wt.%) were added, and the final mixed solution was shaken to obtain a uniform solution. UV–Vis spectrophotometer was used to record the absorption intensities at wavelengths of 220 nm and 275 nm. The calculated absorbance value A ($A={A}_{220{{{{{\rm{nm}}}}}}}-2\times {A}_{275{{{{{\rm{nm}}}}}}}$) is linearly related to the NO_3_^−^ concentrations. In order to quantify the amount of NO_3_^−^, a calibration curve was obtained using KNO_3_ standard solution.

### Calculation of the FE, Y_NH3_ and *j*_NH3_

The FE was defined as the charge consumed for the formation of a specific product (e.g., NH_3_) divided by the total charge passing through the electrodes (Q) during electrolysis. Given that eight electrons are consumed to produce one NH_3_ molecule, the FE of NH_3_ (FE_NH3_), Y_NH3_ and *j*_NH3_ can be calculated as follows: ${{{{{{\rm{FE}}}}}}}_{{{{{{\rm{NH}}}}}}3}=(8\times {{{{{\rm{F}}}}}}\times {C}_{{{{{{\rm{NH}}}}}}3}\times V)/Q$, ${{{{{{\rm{Y}}}}}}}_{{{{{{\rm{NH}}}}}}3}=({C}_{{{{{{\rm{NH}}}}}}3}\times V)/(A\times t)$, and ${j}_{{{{{{\rm{NH}}}}}}3}=(Q\times {{FE}}_{{{{{{\rm{NH}}}}}}3})/(A\times t)$, where *F* is the Faraday constant, *C*_NH3_ is the molar concentration of detected NH_3_, V is the volume of the electrolytes, *A* is the electrode geometric area, and *t* is the reaction time. Given that two electrons are consumed to produce one NO_2_^−^ molecule, the FE of NO_2_^−^ can be calculated as follows:$\,{{{{{\rm{FE}}}}}}({{{{{{\rm{NO}}}}}}}_{2}^{-})=(2\times {{{{{\rm{F}}}}}}\times C({{{{{{\rm{NO}}}}}}}_{2}^{-})\times V)/Q$, where *C*(NO_2_^−^) is the molar concentration of detected NO_2_^−^.

### ^15^NO_3_^−^ Isotope labelling experiments and ^14^NH_3_ quantification by ^1^H NMR

To quantify the ^14^NH_4_^+^ yield after electrolysis of 0.01 M K^14^NO_3_ at −0.175 V (vs. RHE) for 1 h, a calibration curve of ^1^H NMR (400 MHz) measurements was constructed using a series of ^14^NH_4_Cl solutions with defined concentrations (1, 2, 3, 4 and 5 mM) as standards. In a typical procedure^84^, 125 μl of the standard solution/electrolytes was mixed with 125 μl of 15 mM maleic acid in DMSO-D_6_ (99.9 atom% D), 50 μl of 4 M H_2_SO_4_ in DMSO-D_6_ and 750 μl of DMSO-D_6_. The peak area integral ratio of ^14^NH_4_^+^ to maleic acid is positively correlated with the concentrations of ^14^NH_4_^+^. To confirm the source of NH_3_ qualitatively, 0.01 M Na^15^NO_3_ (>98 atom%^15^N, ≥99% purity) and 0.1 M KOH were used as the feeding electrolytes for 1 h electrolysis at −0.175 V (vs. RHE) and ^15^NH_4_^+^ in the electrolyte was detected using ^1^H NMR^84^.

### *Operando* SECM test

To prepare the model catalyst, a CuSP slid ($0.5\times 3$ cm^2^) was cleaned using 0.1 M HCl under ultrasonication for 20 min, washed by deionized water, and dried by blotting paper. Then, half of the CuSP slid was immersed in a MOFs growth solution (a mixture of 1.5 ml of 2-methylimidazole (0.4 M) and 1.5 ml aqueous solution of Co(NO_3_)_2_ 6H_2_O (50 mM)) in Ar-atmosphere for 6 h. The formed Cu_ZIF-Co hybrid layers were washed with deionized water, dried with blotting paper, and further immersed in 1 M KOH solution bubbled with Ar gas for 1.5 h in a gas-tight cell, during which the ZIF-Co film was completely converted into a uniform and compacted Co(OH)_2_ layer, due to the poor stability of ZIF-Co MOFs in water^85^. After washed with water and ethanol and drying at 25 °C, half of the CuSP slide was exposed in Air and the other half was covered by Co(OH)_2_ nanosheets, both of which constitute the Cu (CuO_x_)_Co(OH)_2_ model catalyst.

The SECM setup with shear-force-based distance control is located in a faraday cage to allow for shielding of electrical noise, with the exception of the lock-in amplifier and the potentiostat^86,87^. During the SECM measurements, the Cu_Co(OH)_2_ catalyst were used as the sample (working electrode 1; WE 1), while a Pt-UME with a diameter of ~1 μm (working electrode 2; WE 2), Ag/AgCl/3 M KCl (reference electrode) and a Pt-mesh (counter electrode separated by a Zirfon membrane) were assembled to a four-electrode system. The sample was polarized to −0.12 V (vs. RHE) for triggering the NO_3_RR, while at the Pt-UME cyclic voltammograms were performed in a potential range between −0.12 V and 1.58 V (vs. RHE) at a scan rate of 200 mV s^−1^ to identify intermediately formed NO_2_^−^ (at 0.06 V vs. RHE) and NH_3_ (at 0.76 V vs. RHE). Each SECM array scan was recorded from the Cu/CuO_*x*_ layer to the Co(OH)_2_ layer with an overall x-displacement of 1200 μm, while the border between the two layers is at ~600 μm.

### In situ Raman spectroscopy

Raman spectroscopy was performed with a Lab−RAM HR Raman microscopy system (Horiba Jobin Yvon, HR550) equipped with a 532 nm laser as the excitation source, a water immersion objective (Olympus LUMFL, 60×, numerical aperture = 1.10), a monochromator (1800 grooves/mm grating) and a Synapse CCD detector. Each spectrum is an average of five continuously acquired spectra with a collection time of 50 s each. A three-electrode electrochemical cell was used for in situ Raman tests. Pt wires and Ag/AgCl (3 M KCl) were used as counter and reference electrodes, respectively. To protect the objective from the corrosive 0.1 M KOH electrolyte, 0.01 M KOH (pH 12) was used instead. K_2_SO_4_ (**≥**99.0%) was added to ensure sufficient ionic conductivity (keeping the total concentration of K^+^ to be 0.1 M) and provides SO_4_^2−^ ions as an external Raman reference. Typically, in the presence of 0.01 M KNO_3_, the supporting electrolytes were 0.01 M KOH and 0.04 M K_2_SO_4_. In the absence of KNO_3_, the electrolytes were 0.01 M KOH and 0.045 M K_2_SO_4_.

## Supplementary information

Supporting Information

Description of Additional Supplementary Files

## Data Availability

The data generated and analyzed during this study are provided in the main text and Supplementary information file or can be obtained from the corresponding authors on reasonable request.
