# Supplementary material for: Splicing the active phases of copper/cobalt-based catalysts achieves high-rate tandem electroreduction of nitrate to ammonia
Source: Nat Commun. 2022 Mar 2;13:1129. doi: 10.1038/s41467-022-28728-4 (PMC8891333; doi:10.1038/s41467-022-28728-4)
Supplement: Supplementary file 1 — Supporting Information [file 41467_2022_28728_MOESM1_ESM.pdf]

Splicing the active phases of copper/cobalt−based catalysts achieves high−rate tandem electroreduction of nitrate to ammonia

Wenhui He[1], Jian Zhang[1], Stefan Dieckhöfer[1], Swapnil Varhade[1], Ann Cathrin Brix[1], Anna Lielpetere[1], Sabine Seisel[1], João R. C. Junqueira[1], and Wolfgang Schuhmann[1]*

[1]Analytical Chemistry—Center for Electrochemical Sciences (CES), Faculty of Chemistry and Biochemistry, Ruhr University Bochum, Universitätsstr. 150, D−44780 Bochum (Germany)

* Corresponding authors, e−mail address: wolfgang.schuhmann@rub.de,

Supplementary Figures

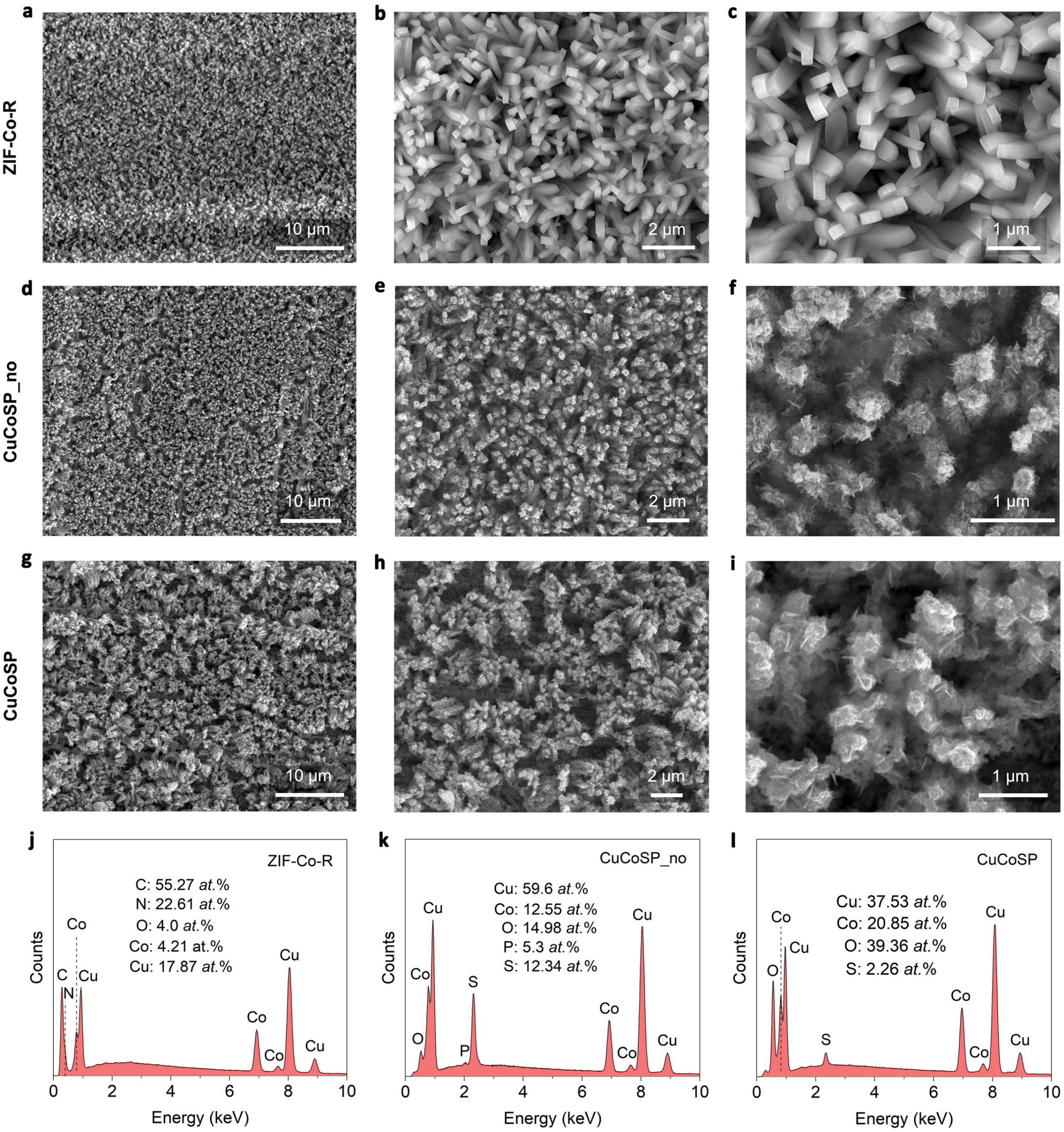

Supplementary Fig. S1 | Morphology and composition of ZIF−Co−R, CuCoSP_no and CuCoSP on Cu foils. a−c, SEM images of ZIF−Co−R nanorods grown on Cu foil with different magnification. **d−f,** SEM images of CuCoSP_no synthesized by electrochemical conversion of ZIF−Co−R. **g−i,** SEM images of CuCoSP obtained from electrochemical redox activation of CuCoSP_no. **j−l,** The corresponding energy dispersive X−ray spectroscopy (EDS) of ZIF−Co−R (**j**), CuCoSP_no (**k**) and CuCoSP (**l**).

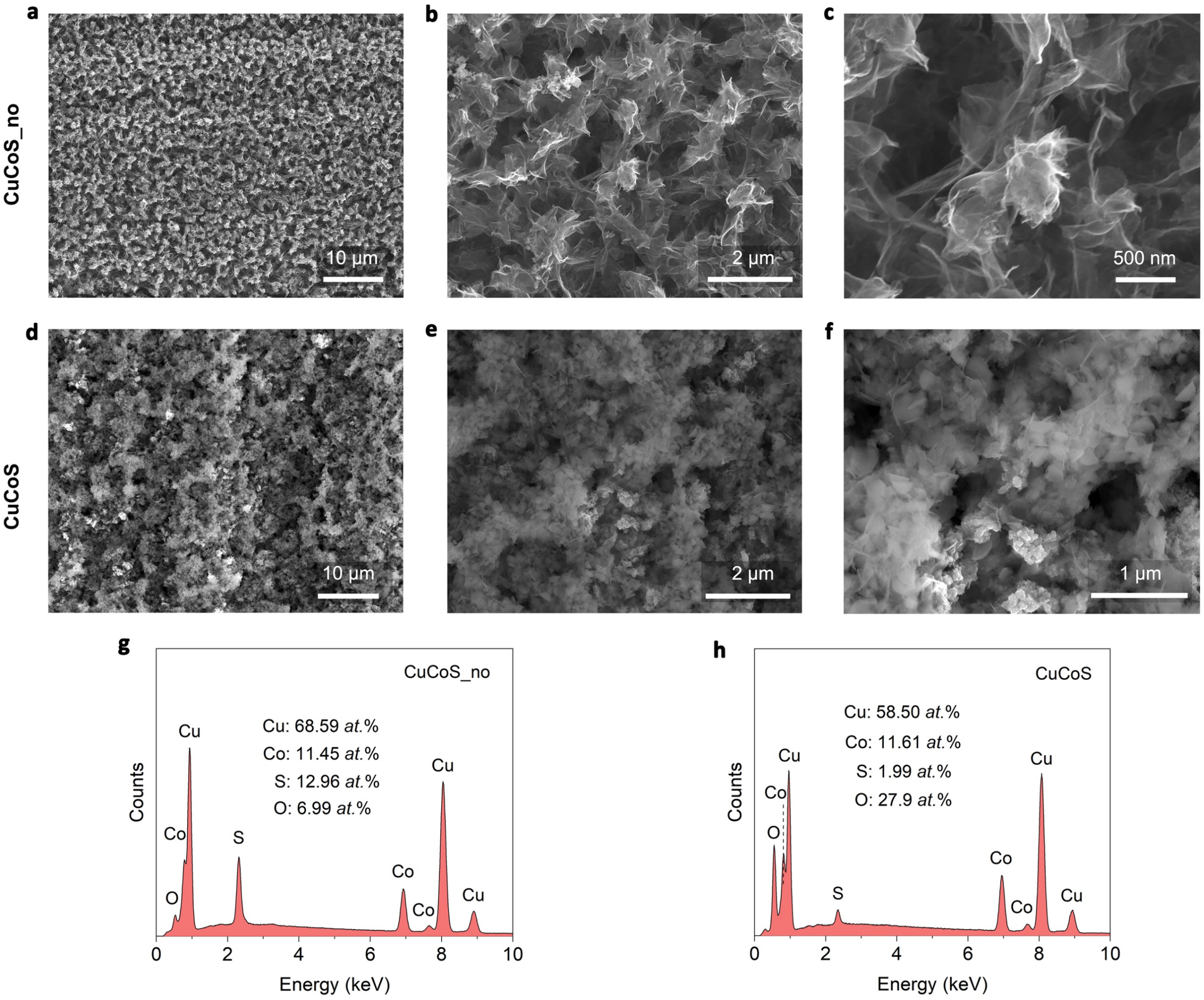

Supplementary Fig. S2 | Morphology and composition of CuCoS_no and CuCoS on Cu foils. a–c, SEM images of CuCoS_no synthesized by electrochemical conversion of ZIF–Co–R on Cu foil without adding 0.05 M Na_2HPO_4 in the electrolytes. **d–f**, SEM images of CuCoS obtained via electrochemical redox activation of CuCoS_no. **g**, **h**, EDS patterns of CuCoS_no (**g**) and CuCoS (**h**).

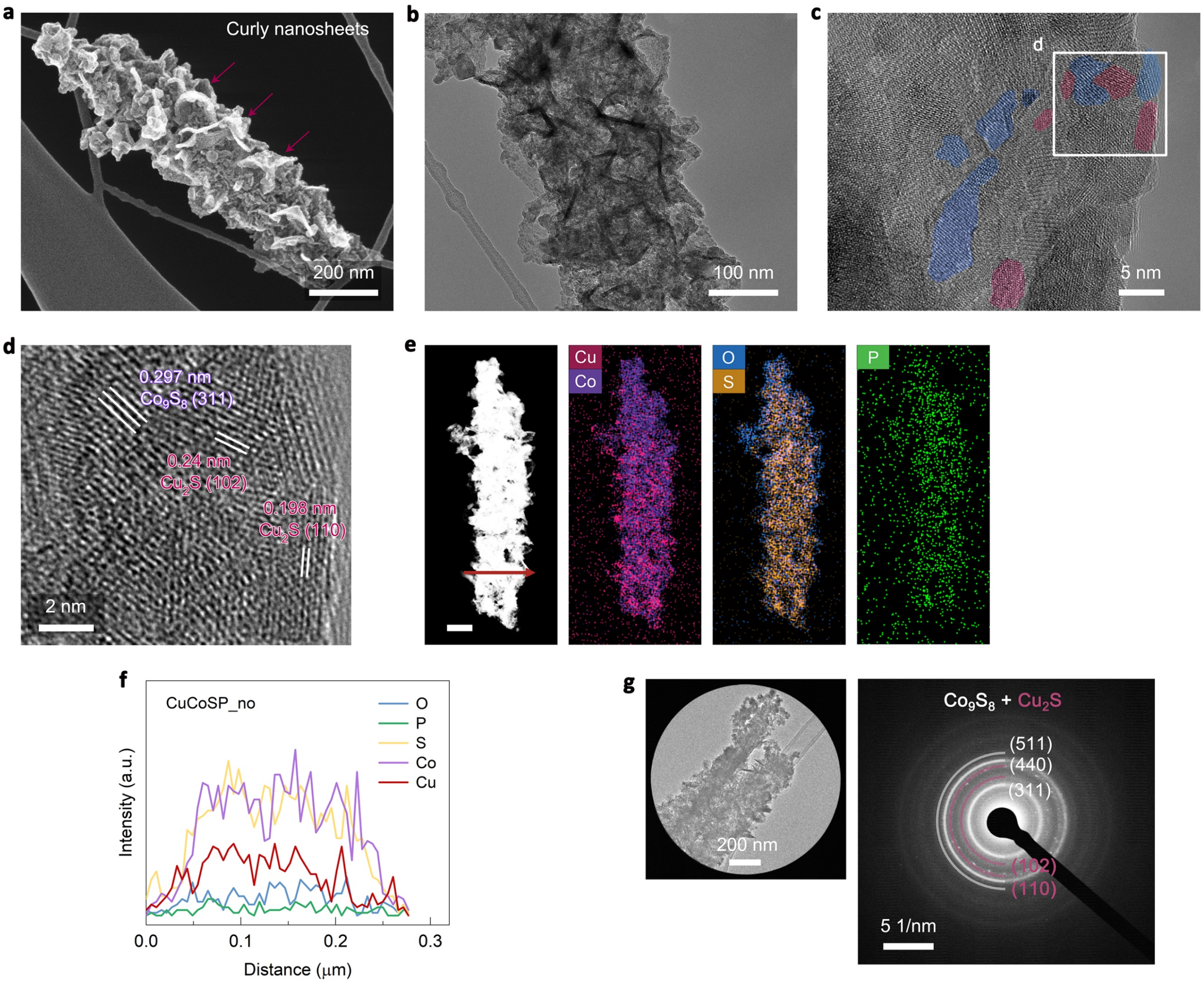

Supplementary Fig. S3 | Nanostructure and element distribution of CuCoSP_no. a, A typical high−resolution SEM (HR−SEM) image of CuCoSP_no, showing the presence of curly nanosheets that correspond to the Cu/Co−based binary metal sulfides. **b–d**, A typical transmission electron microscope (TEM) image and the corresponding high−resolution TEM (HR−TEM) images of CuCoSP_no. The crystalline lattices reveal the coexistence of Co_9S_8 and Cu_2S nanocrystals with a size of about 3−5 nm, as shown in (**c**). The blue and red colors in (**c**) are marked to guide the eye and correspond to Co_9S_8 and Cu_2S nanocrystals, respectively. **e**, **f**, EDX mapping (**e**) and EDX linear scan (**f**) of the area marked by the red arrow in (**e**). Scale bar in (**e**) is 100 nm. **g**, Selected area electron−diffraction (SAED) patterns of CuCoSP_no.

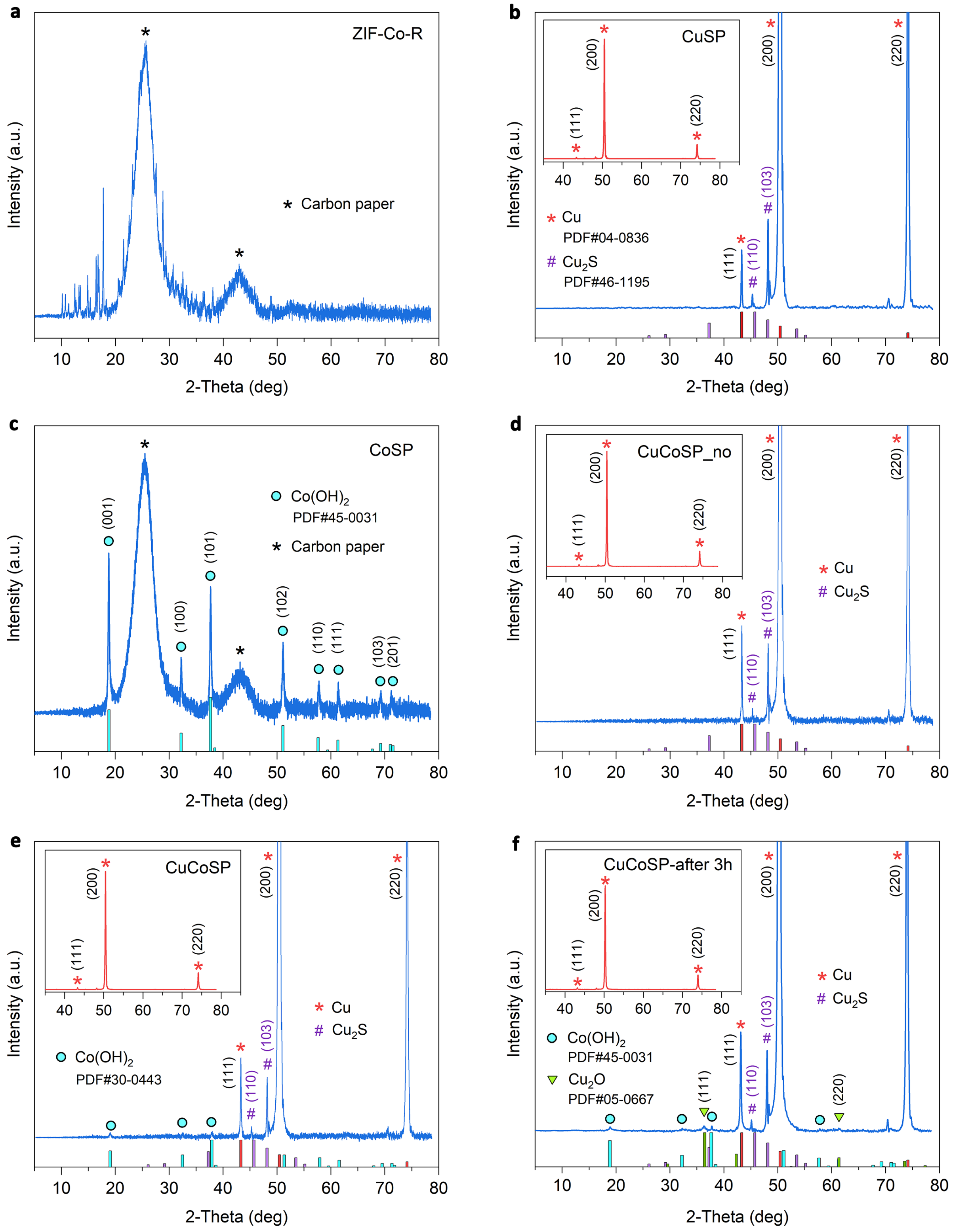

Supplementary Fig. S4 | X-ray diffraction (XRD) patterns of the samples. a, ZIF−Co−R on carbon paper. **b**, CuSP on Cu foil. **c**, CoSP on carbon paper. **d**, CuCoSP_no Cu foil. **e**, CuCoSP on Cu foil. **f**, CuCoSP on Cu foil after repeating three electrolysis of one hour at −0.325 V. All the samples on Cu foils show preferential exposure of the Cu (200) facet, as well as two peaks related to Cu_2S. The latter suggest that there is a stable Cu_2S phase in the Cu foil substrate, which should be produced during the EC−MOF process.

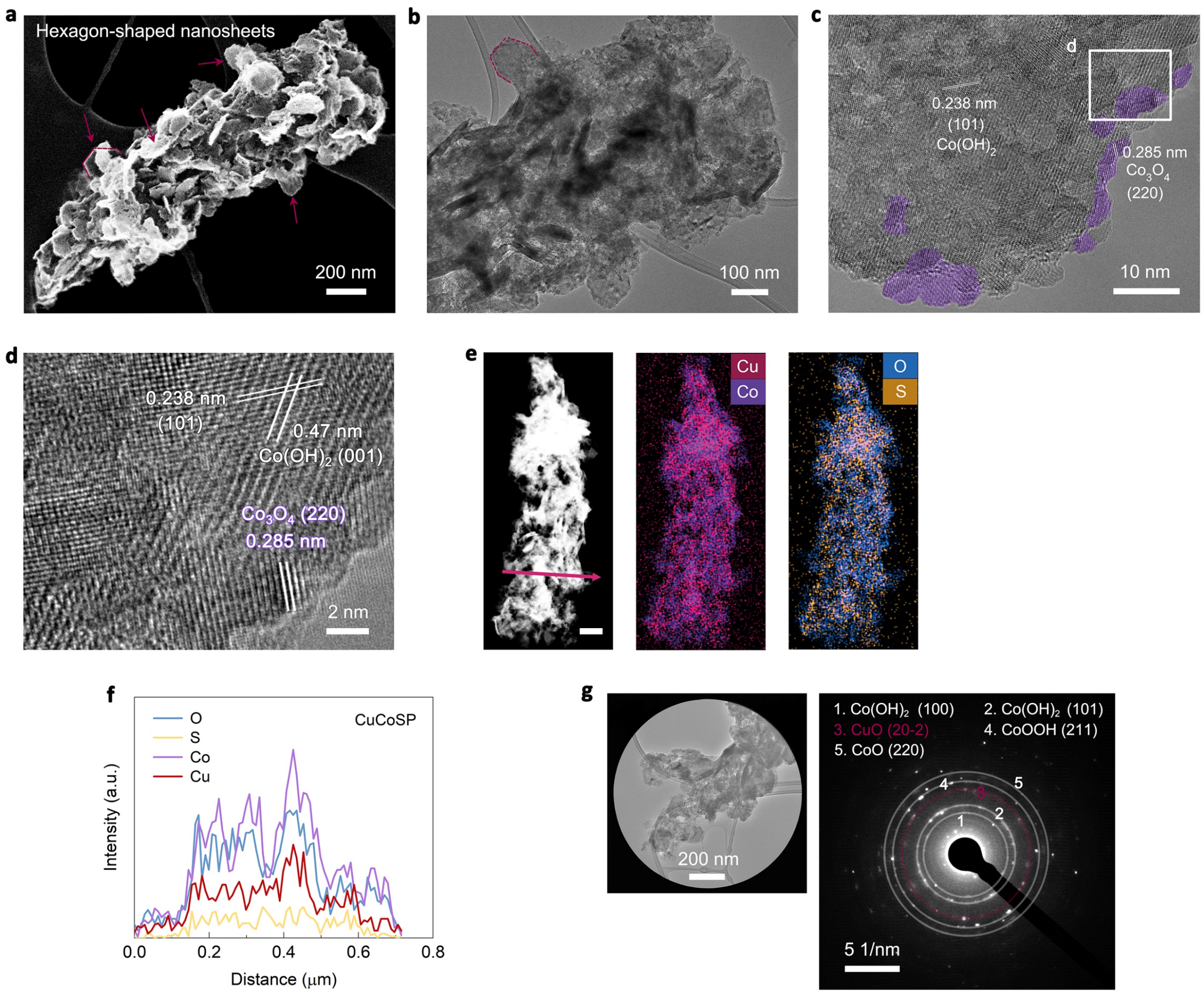

Supplementary Fig. S5 | Nanostructure and element distribution of CuCoSP. a, A typical HR–SEM image of CuCoSP, showing a contour assembled by hexagon–shaped nanosheets, a typical feature of Co–based hydroxides. **b–d**, TEM and HR–TEM images of a hexagon–shaped nanosheet on CuCoSP. The crystalline lattices suggest that the hexagon–shaped nanosheets are mainly composed of $Co(OH)_2$ with few Co_3O_4 nanocrystals mainly distributing at the edge, as shown in (**c**). The purple colors in (**c**) are marked to guide the eye and correspond to the Co_3O_4 nanocrystals. **e**, **f**, EDX mapping (**e**) and linear scan (**f**) of the area marked by the red arrow in (**e**). Scale bar in (**e**) is 200 nm. **g**, SAED patterns of CuCoSP.

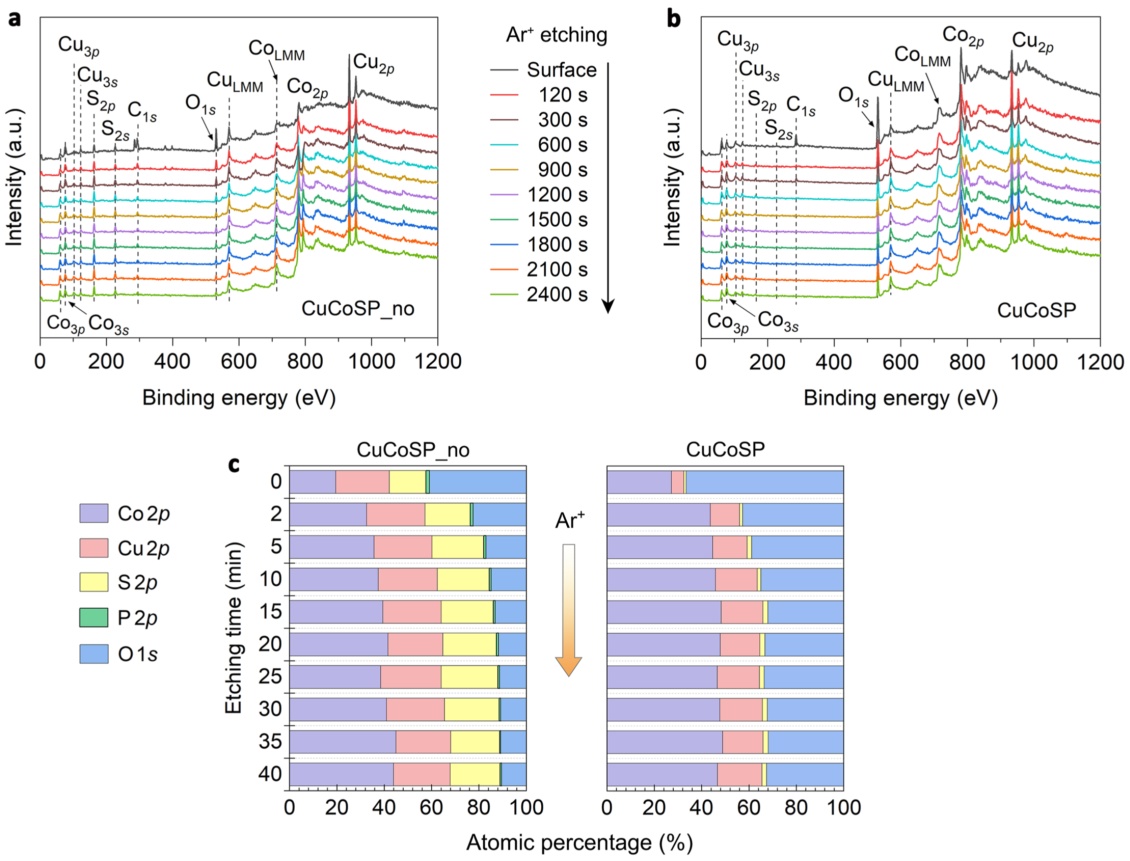

Supplementary Fig. S6 | Depth−profiling X−ray photoelectron spectroscopies (XPS) of CuCoSP_no and CuCoSP. **a**, **b**, XPS spectra of CuCoSP_no (**a**) and CuCoSP (**b**) as a function of Ar^+ etching time. **c**, XPS−determined atomic percentage−depth profile of CuCoSP_no and CuCoSP as a function of Ar^+ etching time.

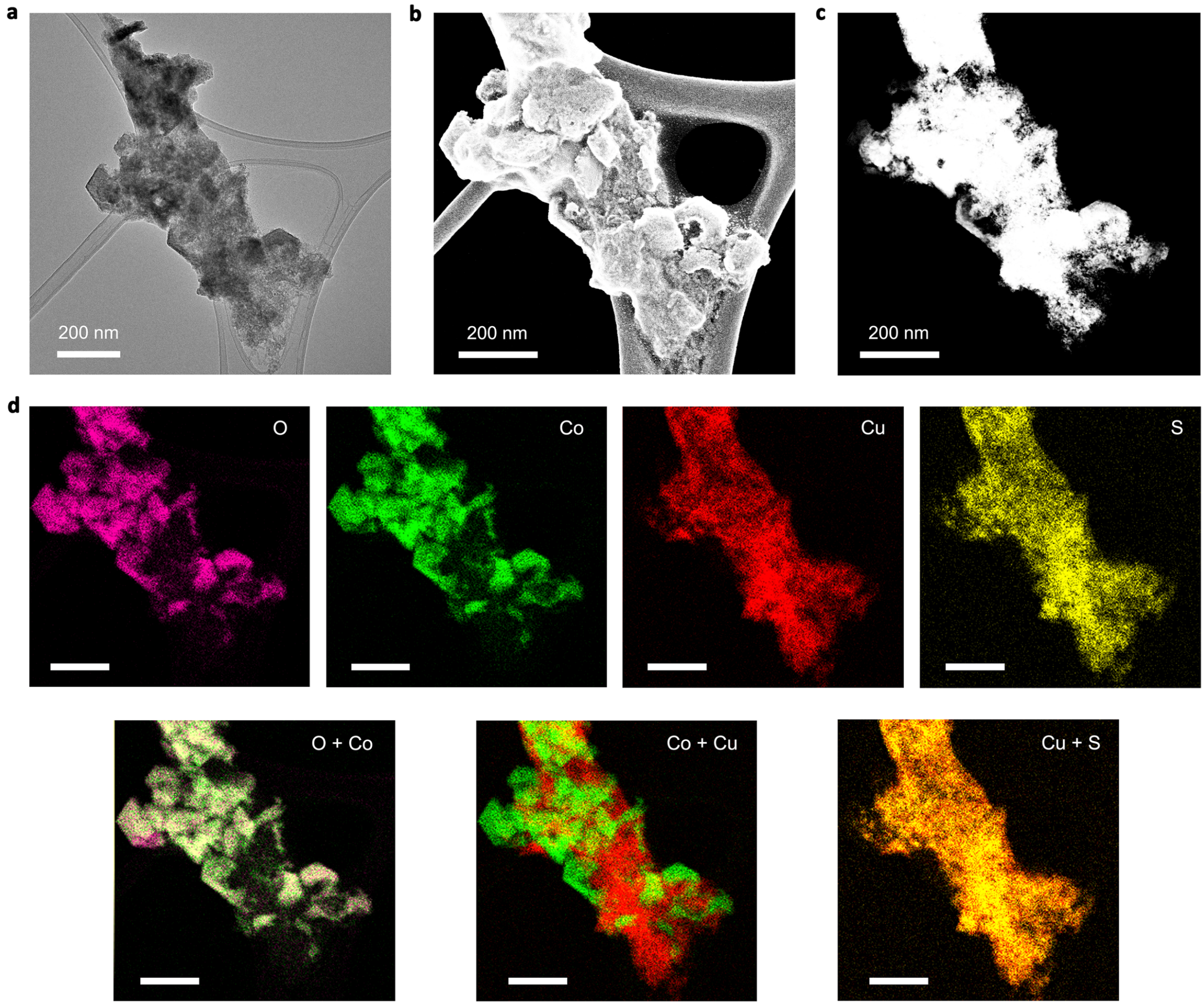

Supplementary Fig. S7 | Phase separation in CuCoSP_no after electrochemical redox activation. a–d, TEM (**a**), HR–SEM (**b**), HAADF–STEM (**c**) and EDX mapping (**d**) images of CuCoSP_no after redox activation. Scale bar in (**d**) is 200 nm. The complete overlapping of O and Co, as well as Cu and residual S elements, suggest that the cobalt sulfide phases are easier to be oxidized than the copper sulfide phases during the redox activation, leading to the separation of Co–based and Cu–based phases, as well as the formation of rich phase interfaces.

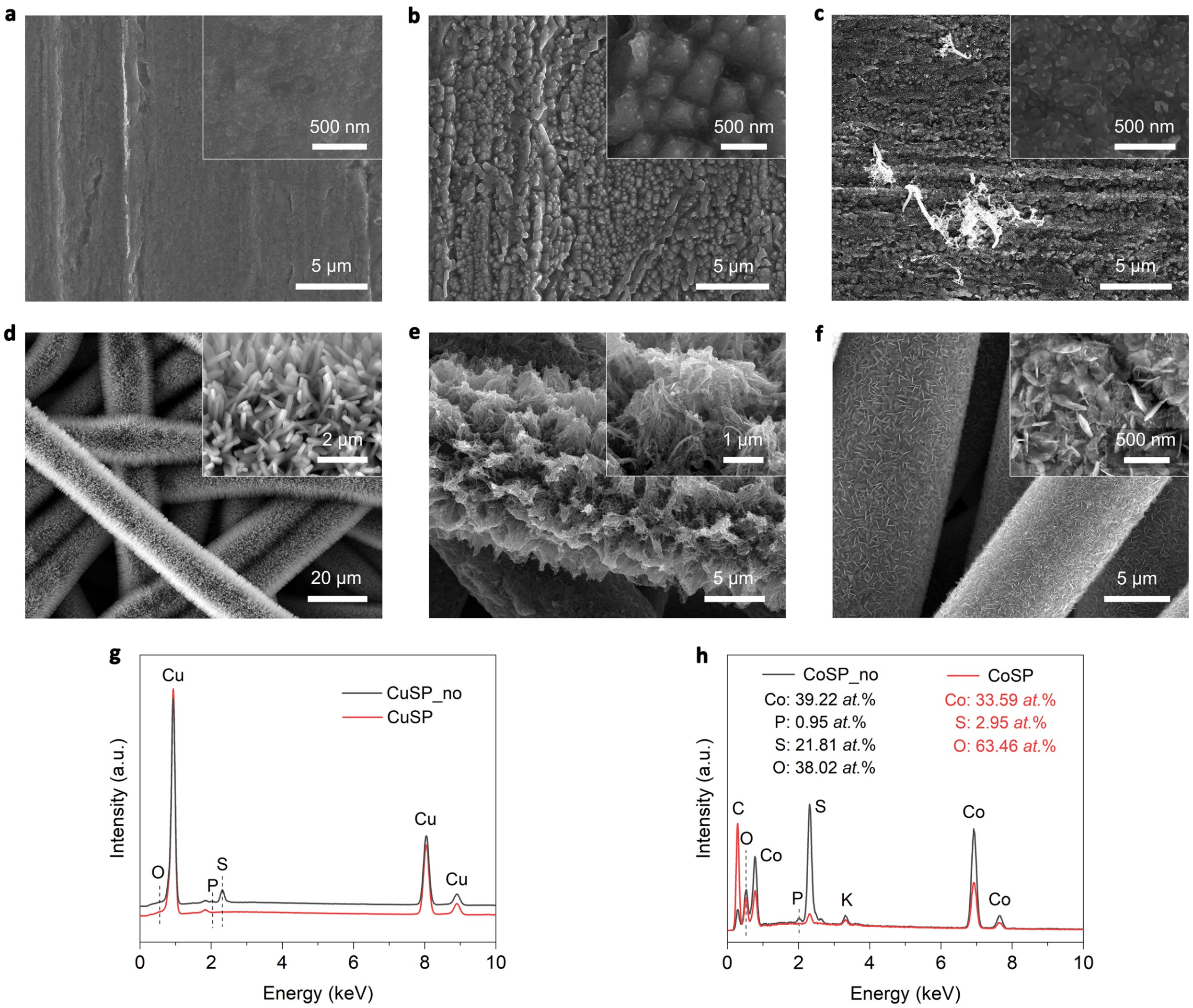

Supplementary Fig. S8 | Morphology and composition of CuSP_no, CuSP, CoSP_no and CoSP. a–c, Typical SEM images of a Cu foil substrate (**a**), CuSP_no (**b**), and CuSP (**c**). **d–f**, Typical SEM images of Co–ZIF–R on carbon papers (**d**), CoSP_no (**e**), and CoSP (**f**). The insets show the corresponding SEM images with higher magnification. **g**, EDS spectra of CuSP_no and CuSP. **h**, EDS spectra of CoSP_no and CoSP.

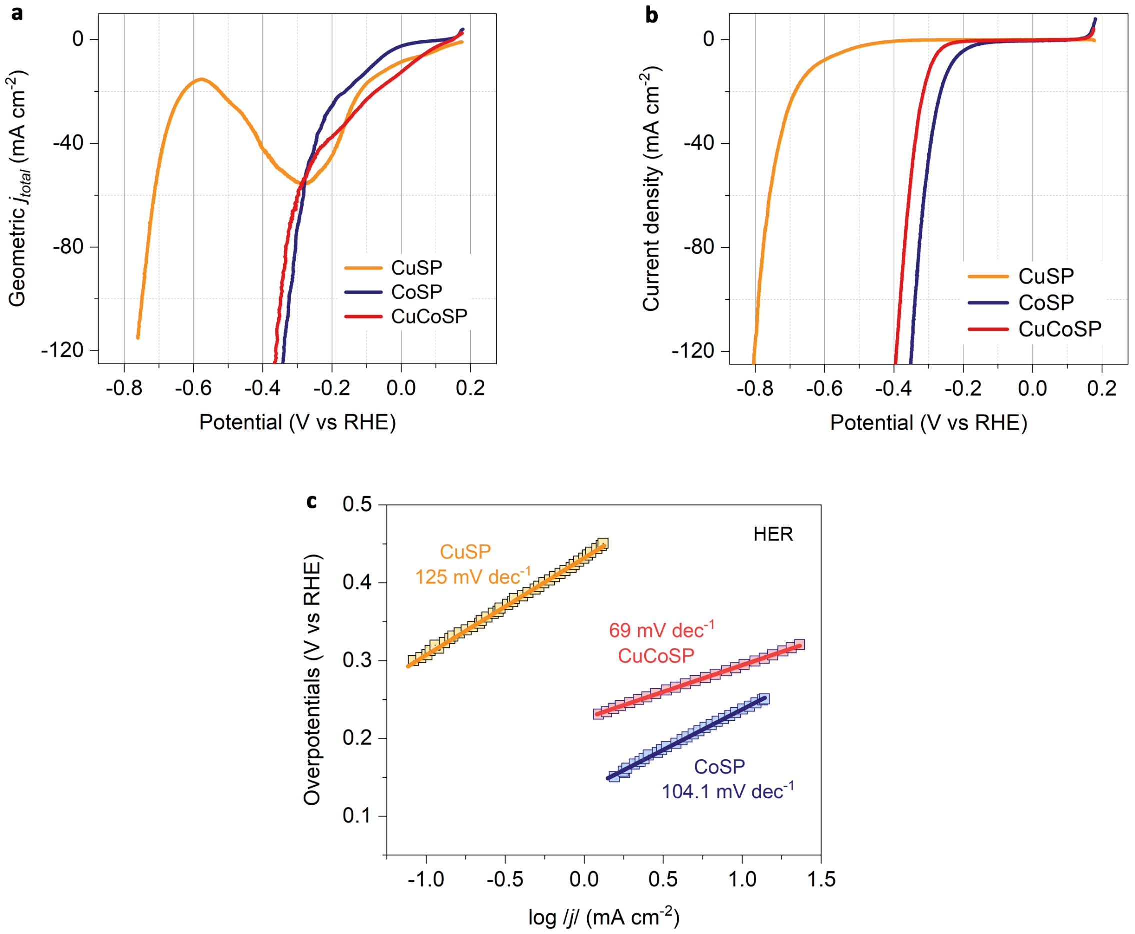

Supplementary Fig. S9 | Linear sweep voltammograms (LSV) tests of CuSP, CoSP and CuCoSP. a–b, LSV curves of CuSP, CoSP and CuCoSP in 0.1 M KOH (pH 13) electrolytes with 0.01 M NO_3^- (**a**) and without NO_3^- (**b**). The LSVs in (**b**) show the H_2–evolving activities (HER) of the catalysts in 0.1 M KOH. **c**, Tafel slopes of the catalysts for HER in 0.1 M KOH. The LSV curves were recorded at a scan rate of 5 mV s^{-1}.

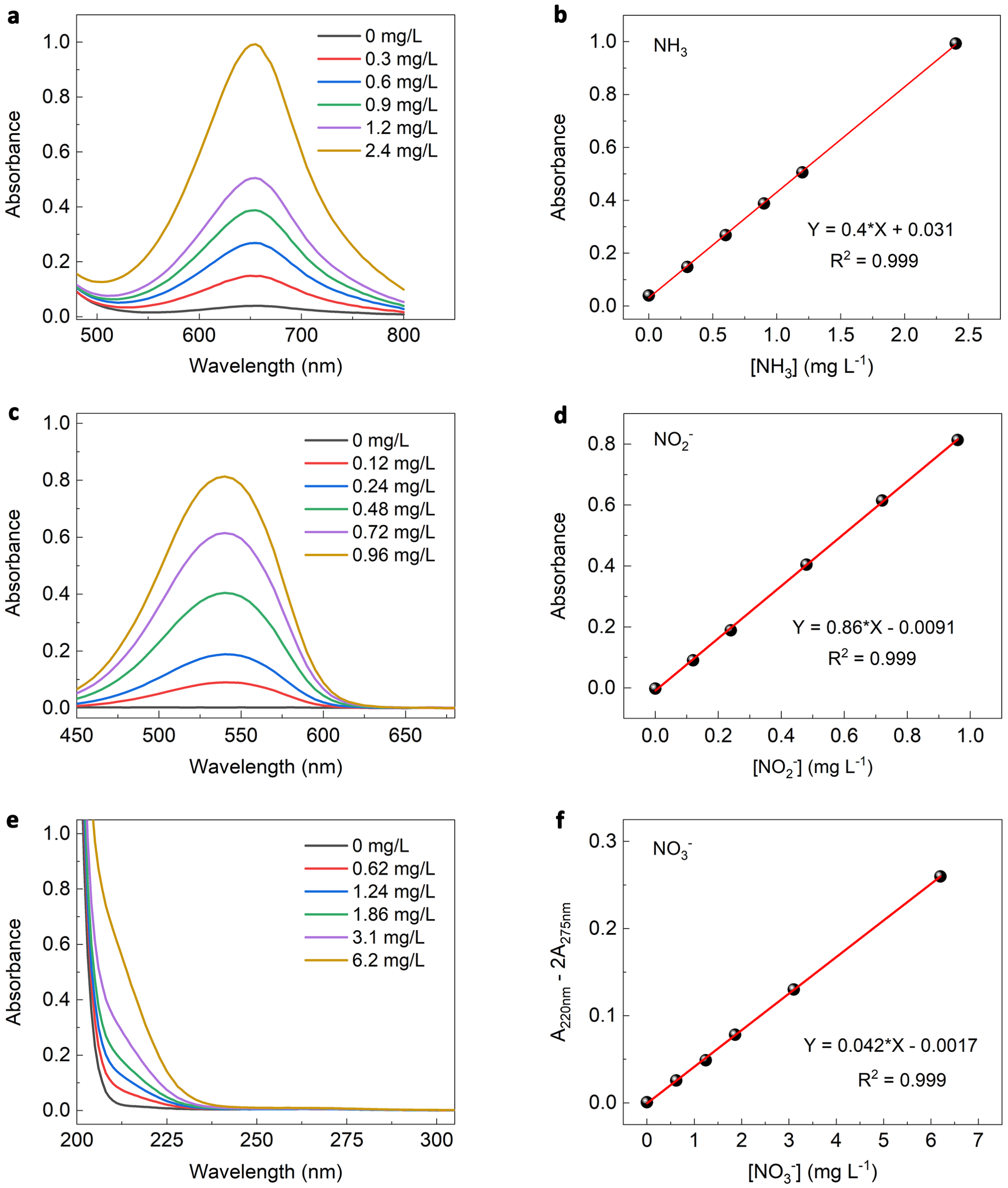

Supplementary Fig. S10 | NH_3, NO_2^- and NO_3^- quantification using UV–vis absorption spectroscopy. **a**, **b**, UV–vis absorption spectra and corresponding calibration curve for the NH_3 assay using the indophenol blue method. **c**, **d**, UV–vis adsorption spectra and corresponding calibration curve for the NO_2^- assay. **e**, **f**, UV–vis absorption spectra and corresponding calibration curve for the NO_3^- assay.

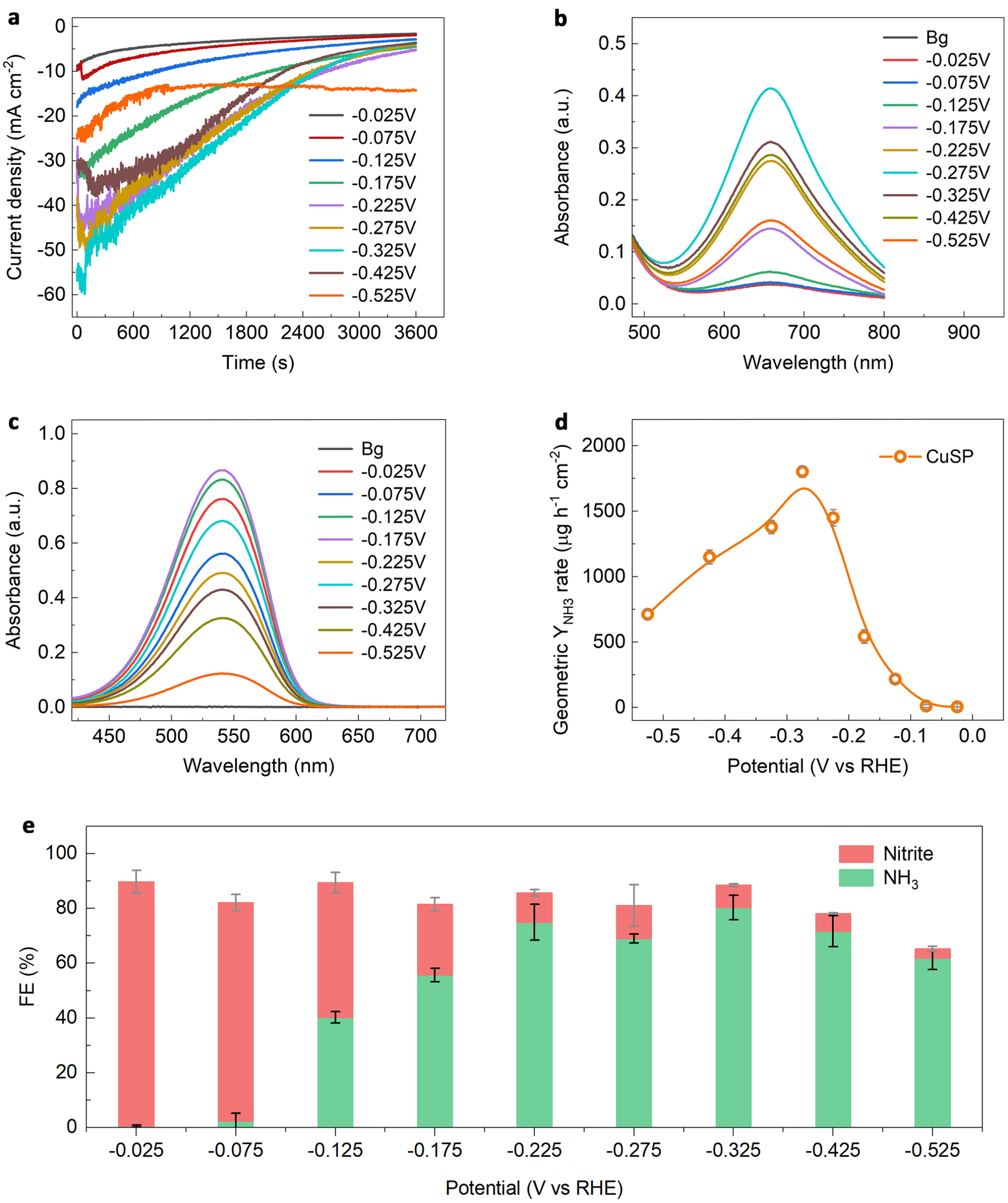

Supplementary Fig. S11 | NH_3 synthesis performance of CuSP at a series of potentials. **a**, Chronoamperometry curves at different potentials for 1 h in 0.1 M KOH and 0.01 M NO_3^-. **b**, **c**, UV–vis absorption spectrum of NH_3 (**b**) and NO_2^- (**c**) measurements. Note that to detect the NH_4^+, the post–electrolysis electrolytes at −0.025 V and −0.075 V were diluted 6 times, while at the other electrolysis potentials, the electrolytes were diluted 30 times. As for the NO_2^- tests, the electrolytes at all the potentials were diluted 50 times. Background (Bg) signals were recorded using 0.1 M KOH. **d**, NH_3 yield rate (Y_{NH3}) of CuSP defined by the geometric area of electrodes. **e**, Faradaic efficiency (FE) of NH_3 and NO_2^-. Error bars denote the standard deviations of Y_{NH3} and FE calculated from three independent samples.

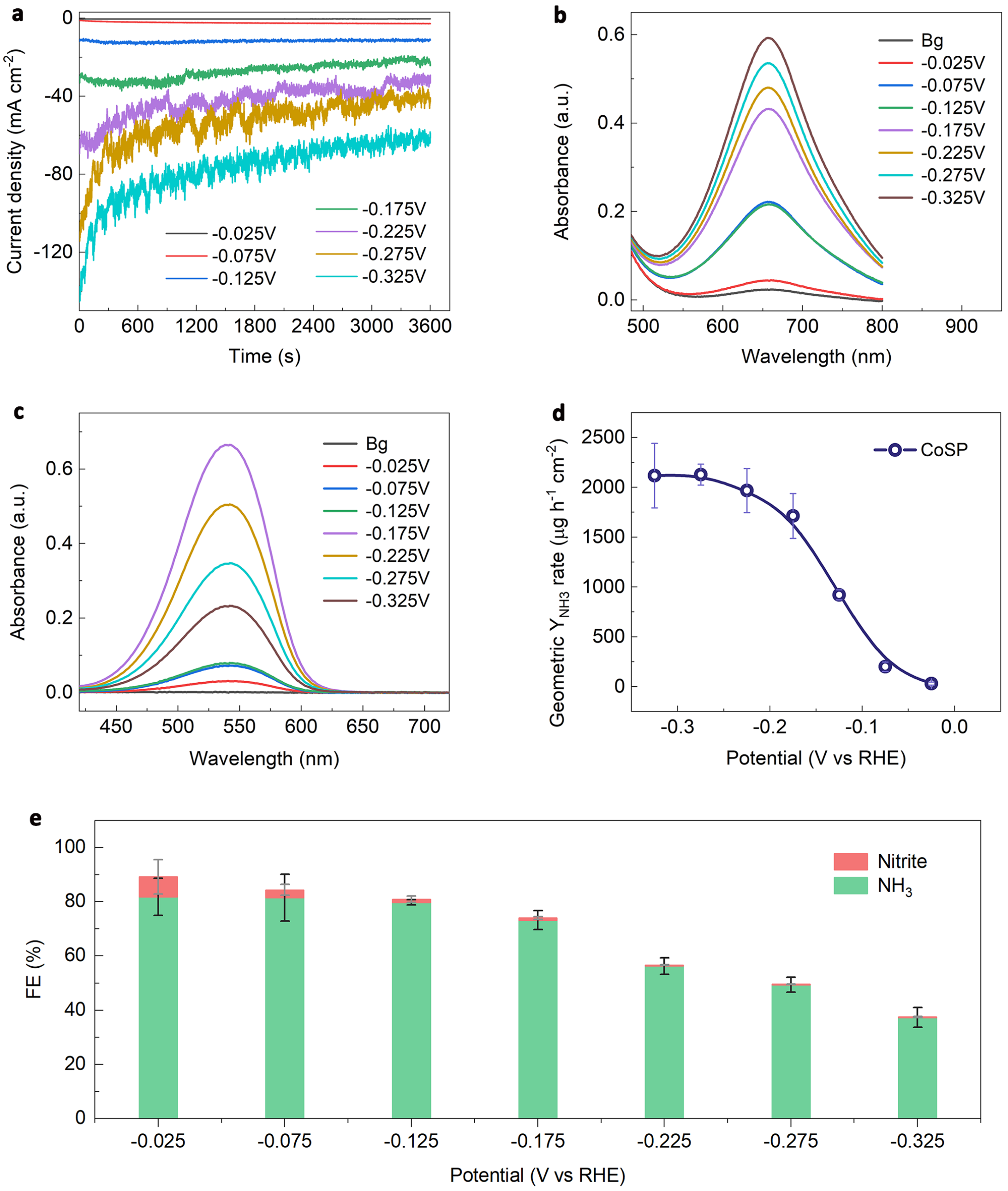

Supplementary Fig. S12 | NH_3 synthesis performance of CoSP at a series of potentials. a, Chronoamperometry curves at different potentials for 1 h in 0.1 M KOH and 0.01 M NO_3^-. **b**, **c**, UV–vis absorption spectrum of NH_3 (**b**) and NO_2^- (**c**) measurements. Note that to detect the NH_4^+, the post–electrolysis electrolytes at −0.025 V and −0.075 V were diluted 6 times, while the electrolytes obtained at the other potentials were diluted 30 times. As for the NO_2^- tests, the electrolytes obtained at all the potentials were diluted 5 times. Background (Bg) signals were recorded using 0.1 M KOH. **d**, Y_{NH3} of CoSP defined by the geometric area of electrodes. **e**, FE of NH_3 and NO_2^-. Error bars denote the standard deviations of Y_{NH3} and FE calculated from three independent samples.

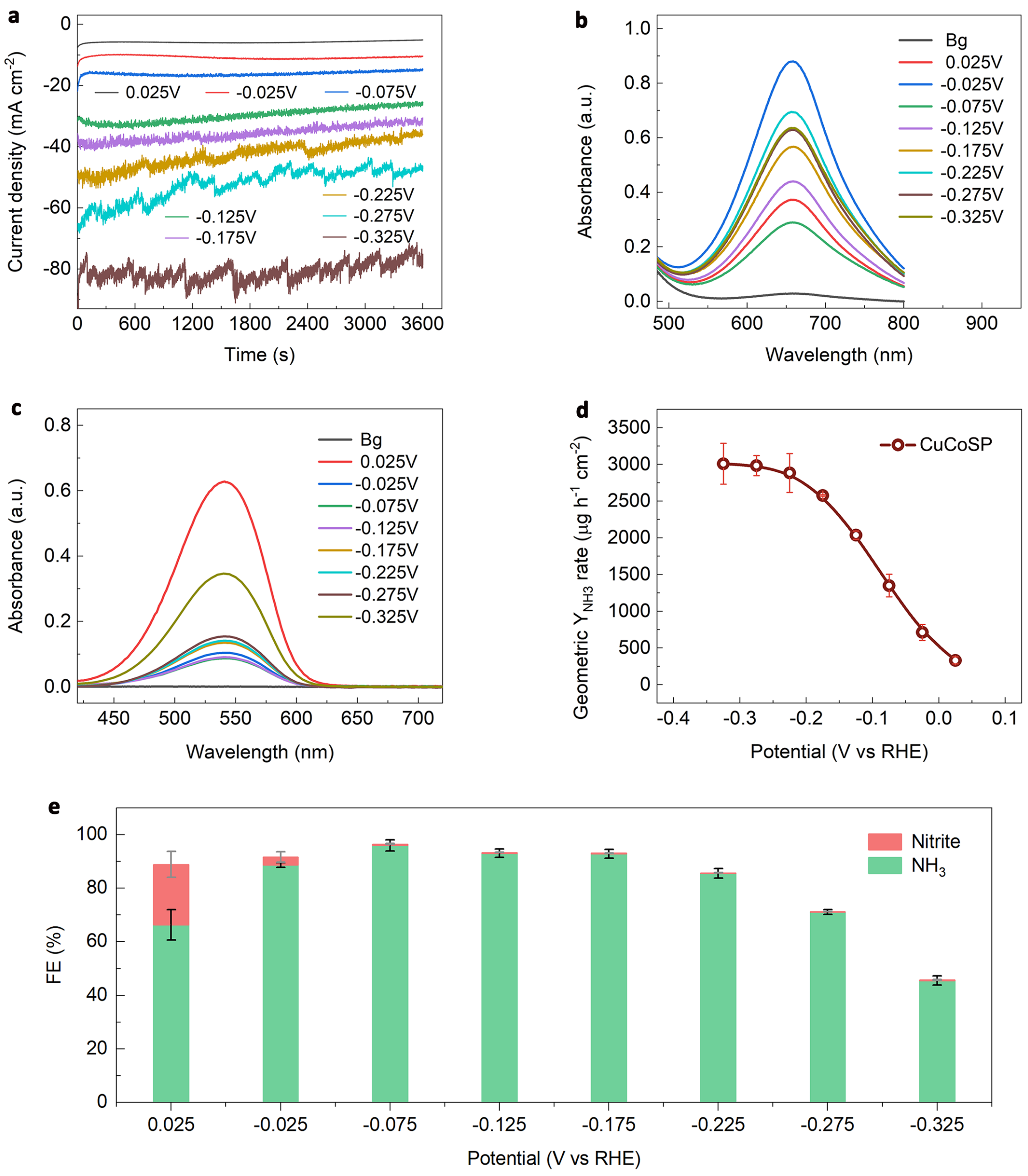

Supplementary Fig. S13 | NH_3 synthesis performance of CuCoSP at a series of potentials. a, Chronoamperometry curves at different potentials for 1 h in 0.1 M KOH and 0.01 M NO_3^-. **b**, **c**, UV–vis absorption spectrum of NH_3 (**b**) and NO_2^- (**c**) measurements. Note that to detect the NH_4^+, the post–electrolysis electrolytes at 0.025 V and −0.025 V were diluted 6 times, while the electrolytes obtained at the other electrolysis potentials were diluted 30 times. As for the NO_2^- tests, the electrolytes obtained at all the electrolysis potentials were diluted 5 times. Background (Bg) signals were recorded using 0.1 M KOH. **d**, Y_{NH3} yield rate of CuCoSP defined by the geometric area of electrodes. **e**, FE of NH_3 and NO_2^-. Error bars denote the standard deviations of Y_{NH3} and FE calculated from three independent samples.

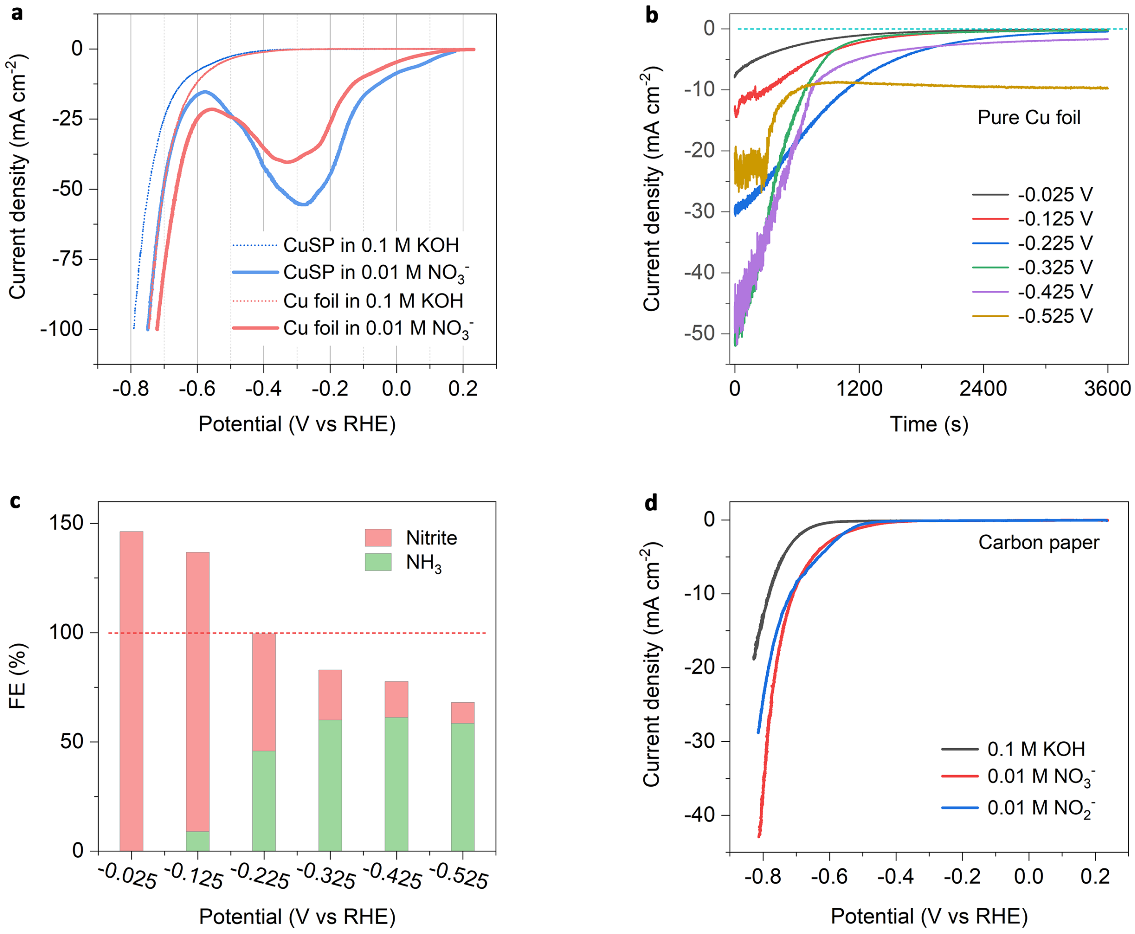

Supplementary Fig. S14 | Activity of pure Cu foil and carbon paper for the NO_3RR. a, Comparison of LSVs recorded on CuSP and Cu foil in 0.1 M KOH in presence and absence of 0.01 M NO_3^-. **b**, Chronoamperometry for 1 h of a Cu foil at different potentials in 0.1 M KOH and 0.01 M NO_3^-. **c**, FE of NH_3 and NO_2^- for Cu foil after one−hour electrolysis at different potentials. **d**, LSVs of carbon paper in 0.1 M KOH with 0.01 M NO_3^-, NO_2^-, or in 0.1 M KOH. LSVs in (**a**) and (**d**) were recorded at a scan rate of 5 mV s^{-1}.

The pure Cu foil has a smooth surface and shows a low electrochemical capacitance (0.126 mF cm^{-2}) (supplementary **Fig. S8a** and **S17**). The Cu foil shows almost the same features of LSV curves for the NO_3RR as the CuSP catalysts, except for lower current densities. However, the Cu foil exhibits a stable NO_3RR activity only at a potential of −0.525 V, which is the same as for the CuSP. At potentials >−0.425 V (vs RHE), the NO_3RR activity of the Cu foil deactivated much more rapidly than that of the CuSP catalyst. The final activity (indicated by the current density) of the Cu foil after one−hour electrolysis is only 5−10% of that of the CuSP activity. This result can be partially explained by the ~17 times smaller ECSA of the Cu foil compared to the CuSP catalysts (supplementary **Fig. S17**). However, as shown in supplementary **Fig. S14c** the Cu foil shows an over 100% FE for NO_3RR at −0.025 V (146 %) and −0.125 V (137 %), with the NO_2^- as the sole or main product, respectively. This result suggests that there is a chemical pathway for NO_2^- formation during electrolysis; i.e., NO_3^- is first electrochemically reduced to NO_2 (one electron transfer), and then the highly oxidative NO_2 species are chemically reduced to NO_2^- by metallic Cu (one electron transfer)[1], resulting in the formation of Cu oxides in alkaline media. This chemical pathway offers additional electrons for the NO_3RR, thus enabling an over 100% FE.

The comparison of the performance of Cu foil and CuSP for the NO_3RR provides two important hints: 1) pure metallic catalysts may play a role of chemical reducing reagents for promoting the NO_3RR, especially at low overpotentials where NO_2 will be easily produced[1], 2) the presence of CuO_x phases in CuSP can help to accelerating the direct reduction of NO_3^- to NO_2^- and in turn decease the formation of oxidative NO_2 species, as supported by the FE of CuSP for the NO_3RR (supplementary **Fig. S11**).

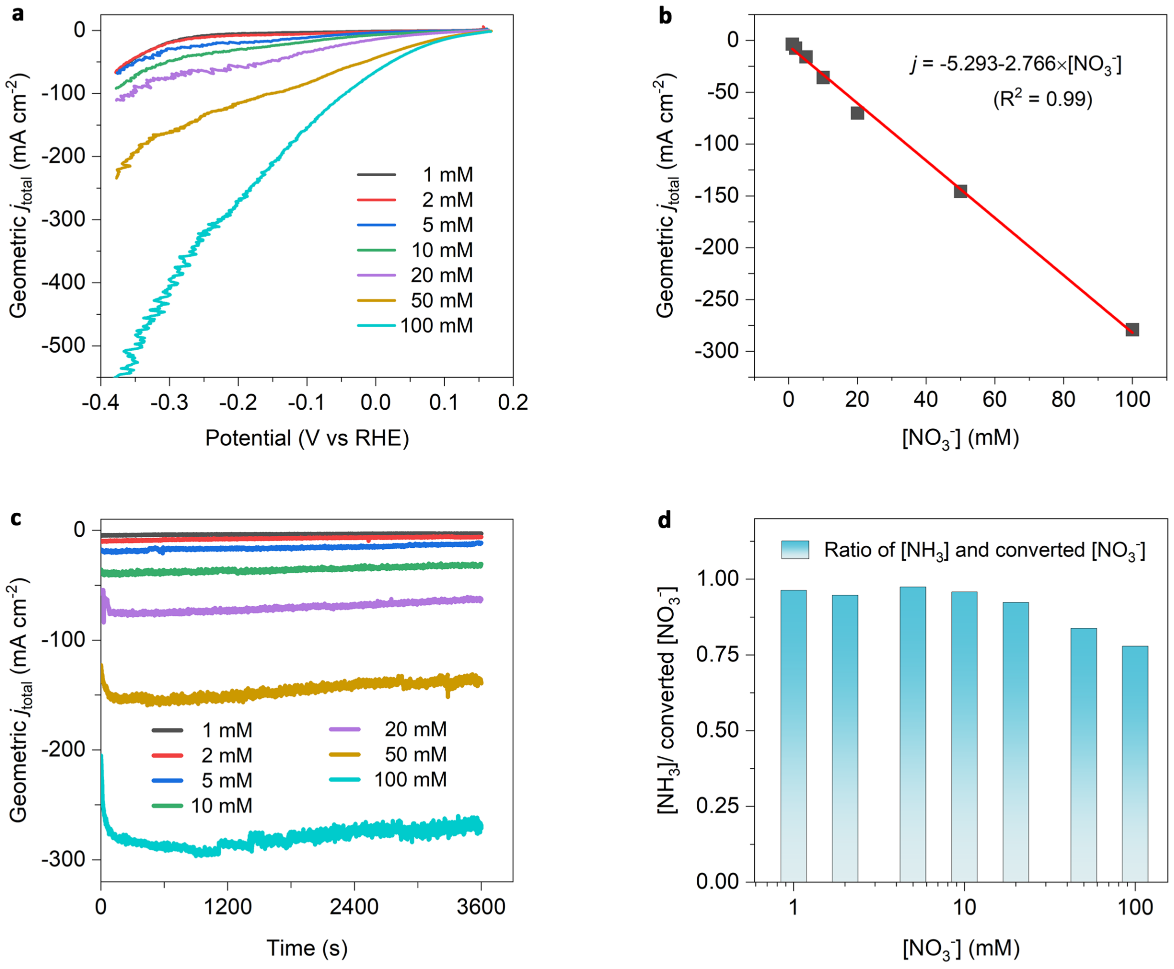

Supplementary Fig. S15 | Electrocatalytic tests of CuCoSP in different concentrations of NO_3^-. a, LSVs of CuCoSP in 0.1 M KOH containing different concentrations of NO_3^- at a scan rate of 5 mV s^{-1}. **b**, Plots of the total current densities (defined by the geometric area of CuCoSP electrodes) at −0.175 V (vs. RHE) against the NO_3^- concentrations ([NO_3^-]). The NO_3^- reduction over CuCoSP follows a first reaction order with respect to [NO_3^-]. **c**, Chronoamperometry curves of CuCoSP under different [NO_3^-] at −0.175 V (vs. RHE). **d**, The turnover numbers (TON) of nitrate on CuCoSP, defined by the ratio of the formed NH_3 concentration [NH_3] to the converted NO_3^- concentration [NO_3^-], which were estimated after one−hour electrolysis at −0.175 V in electrolytes containing different [NO_3^-]. Note that the TON values decrease down to ~0.8 at [NO_3^-] >20 mM, indicating that high [NO_3^-] modulate the pathways of NO_3^- reduction on CuCoSP electrocatalysts, resulting in the formation of gaseous by−products (i.e. N_2 and NO_x).

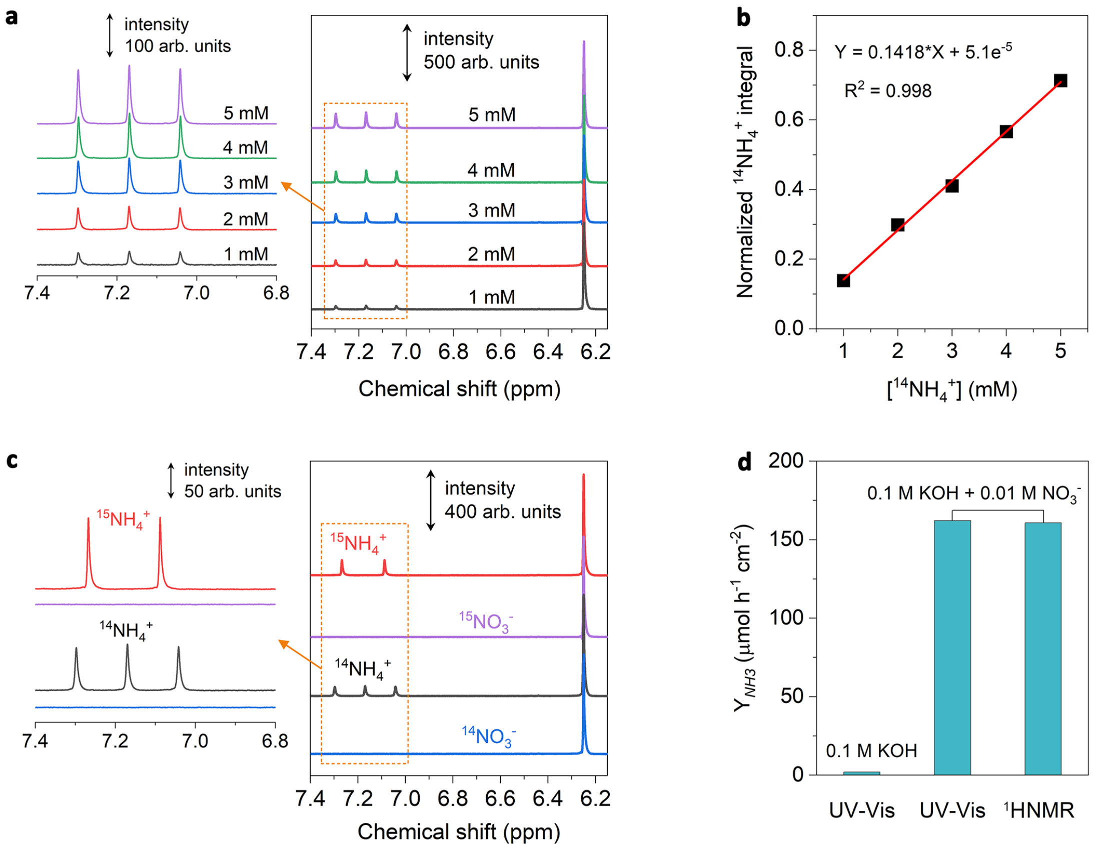

Supplementary Fig. S16 | $^{15}NH_4^+$ detection and $^{14}NH_4^+$ quantification by ^1H NMR spectra. **a**, ^1H NMR spectra of $^{14}NH_4^+$ ions with different concentrations. Maleic acid with a constant concentration was used as an external standard with a proton signal at δ = 6.25 ppm. **b**, Calibration curve for $^{14}NH_4^+$ detection using ^1H NMR, where $^{14}NH_4^+$ peak area integrals were normalized to that of maleic acid. The normalized peak area integral of $^{14}NH_4^+$ is positively correlated to the concentrations of $^{14}NH_4^+$ [$^{14}NH_4^+$]. **c**, ^1H NMR spectra of the electrolytes after electrocatalysis using 0.01 M $^{15}NO_3^-$ or 0.01 M $^{14}NO_3^-$ in 0.1 M KOH as nitrogen source. ^1H NMR of the fresh electrolytes without going through electrolysis (marked as $^{15}NO_3^-$ and $^{14}NO_3^-$) were provided as controls, showing no background signals of ammonia. **d**, Comparison of the ammonia yield rate over CuCoSP quantified by the UV–Vis spectra and ^1H NMR. The electrolysis was carried out at −0.175 V (vs. RHE) for one hour in 0.1 M KOH with and without 0.01 M $^{14}NO_3^-$.

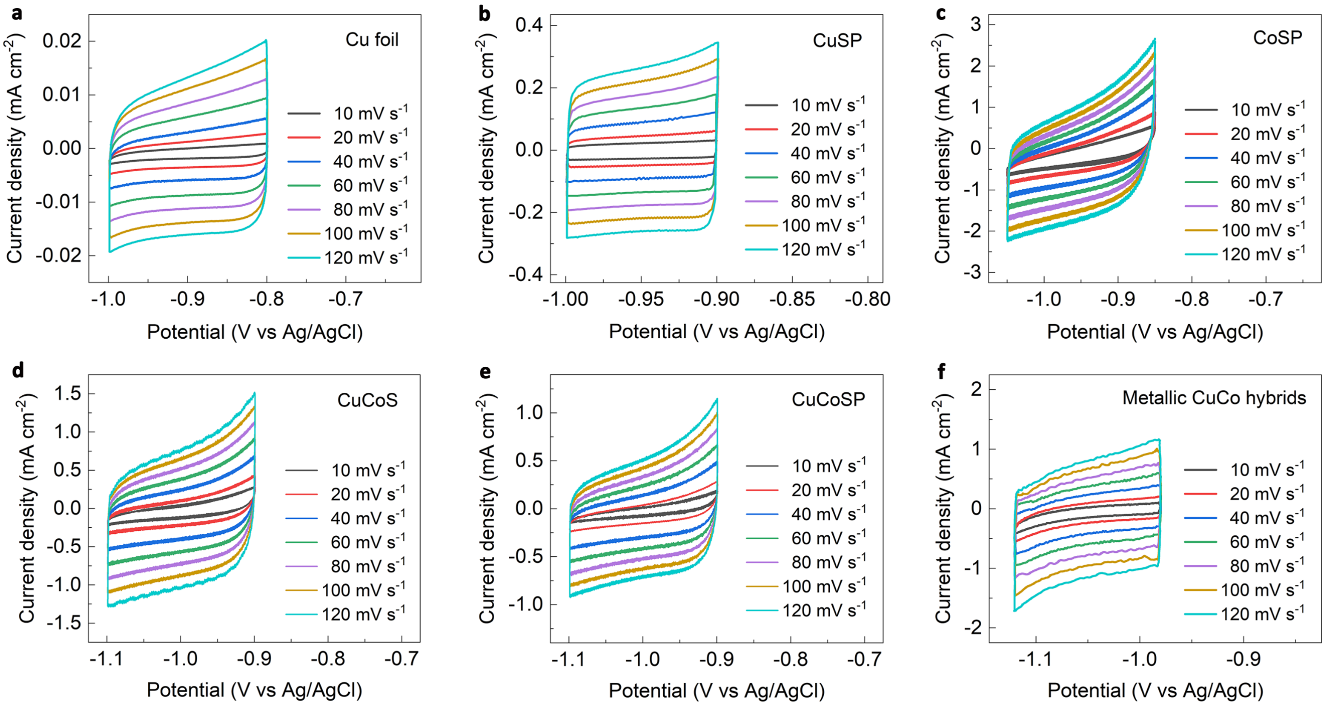

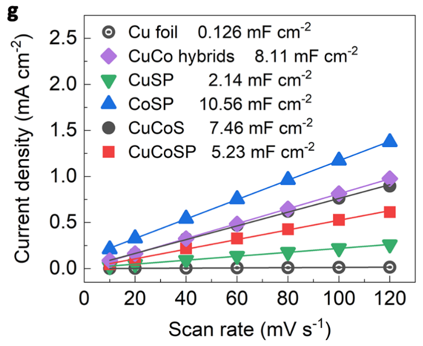

h

Samples	Capacitance per electrode area (mF cm^{-2})	ECSA (cm^{-2})
**Cu foil**	0.126	1
**CuSP**	2.14	16.98
**CoSP**	10.56	83.81
**CuCoSP**	5.23	41.51
**CuCoS**	7.46	59.21
**CuCo hybrids**	8.11	64.37

Supplementary Fig. S17 | Cyclic voltammograms (CV) for the determination of the double−layer capacitance of different samples in Ar−saturated 0.1 M KOH. a, Cu foil, **b**, CuSP. **c**, CoSP. **d**, CuCoS. **e**, CuCoSP. **f**, Metallic CuCo hybrids. **g**, Plots of the current densities against CV scan rates. The slope is correlated with the electrochemical double−layer capacitance per geometric area of the electrode and shows also a correlation to the electrochemical surface area (ECSA). **h**, A table of the double−layer capacitance derived ECSA, where the ECSA of a smooth Cu foil is defined to be 1 cm^2 and the ECSA of the other samples is derived from their double−layer capacitance per geometric electrode area.

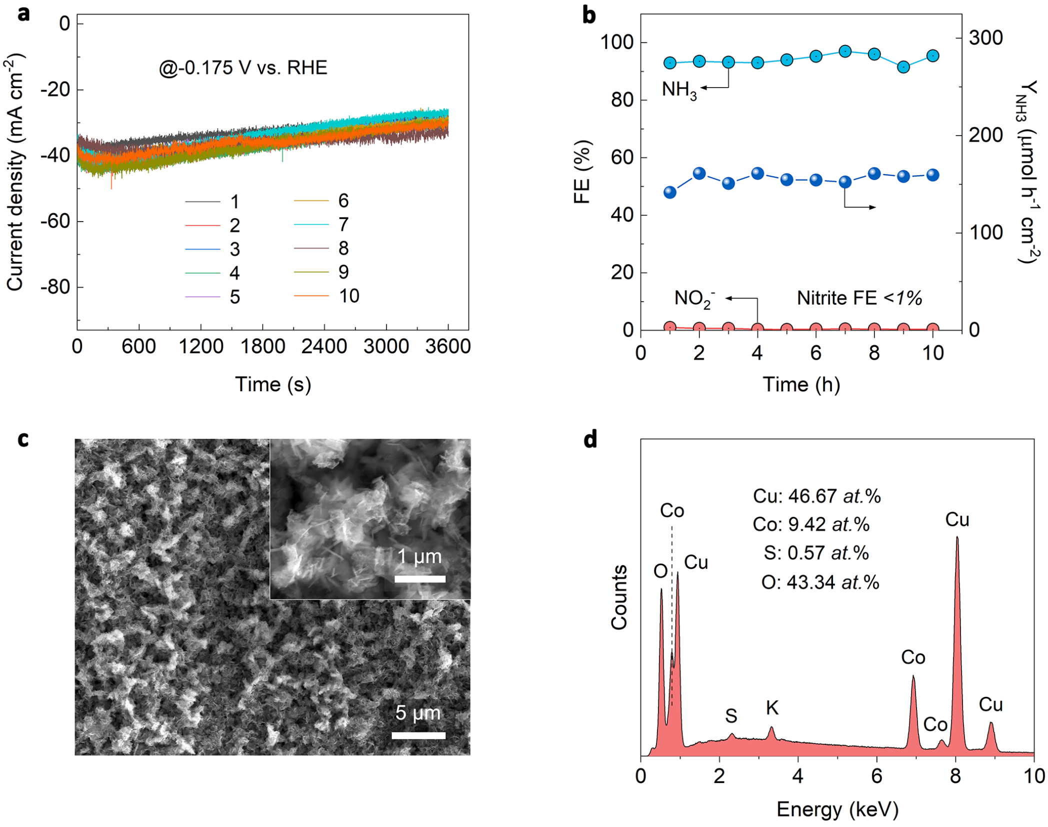

Supplementary Fig. S18 | Stability tests of CuCoSP for ammonia synthesis carried out via repeating one−hour electrolysis for ten times at −0.175 V (vs. RHE). a, Chronoamperometry curves of CuCoSP at different time periods. **b**, Faradaic efficiency and NH_3 yield rate summed by ten independent one−hour tests at −0.175 V. **c**, SEM images of CuCoSP after the stability tests. **d**, EDS patterns of CuCoSP after the stability tests.

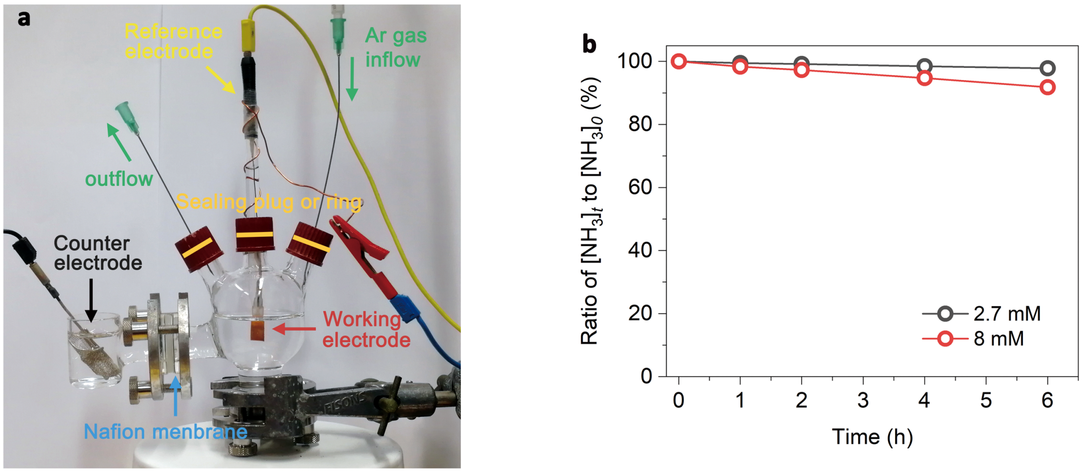

Supplementary Fig. S19 | Ammonia volatilization in the 0.1 M KOH (pH 13) with time. a, Photograph of the H−type cell used in this study. **b**, Change of ammonia concentrations with time. The solution is in the H−type cell under stirring (300 rpm) and Ar flow (10 ml min^{-1}). The initial concentrations of ammonia at t = 0 ($[NH_3]_0$) are 2.7 mM and 8 mM, which correspond to the ammonia yield on CuCoSP after 1 h electrolysis in 0.01 M and 0.1 M nitrate, respectively. The concentration of ammonia at t = 1−6 h is marked as $[NH_3]_t$.

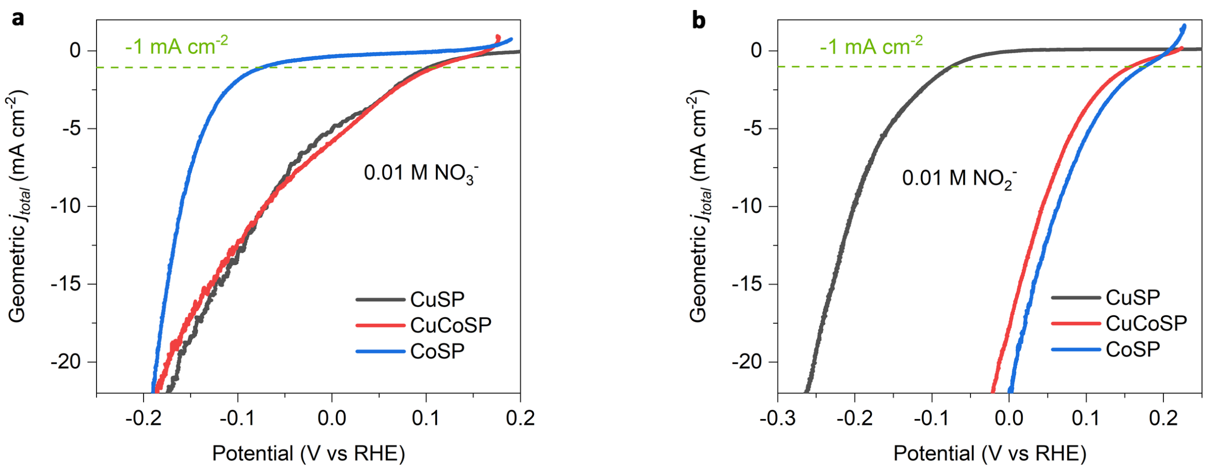

Supplementary Fig. S20 | LSVs of the catalysts recorded at a scan rate of 1 mV s⁻¹. a, in 0.1 M KOH and 0.01 M NO_3^-. **b**, in 0.1 M KOH and 0.01 M NO_2^-. The potentials at −1 mA cm^{-2} (a low current density with negligible mass transferring effects) were marked and used to compare the activities of the catalysts.

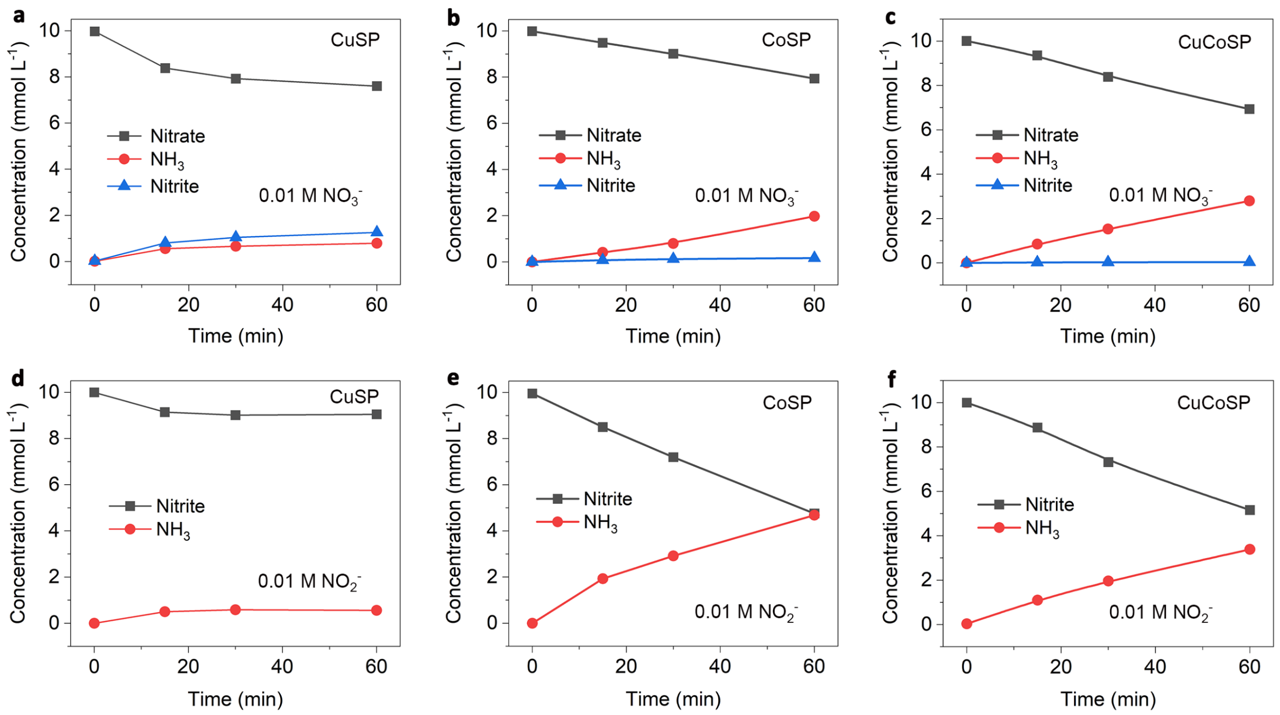

Supplementary Fig. S21 | Concentration−evolving profiles of NO_3^- and NO_2^- ions at −0.175 V vs. RHE. a−c, in 0.1 M KOH and 0.01 M NO_3^- using CuSP (**a**), CoSP (**b**), and CuCoSP (**c**) as catalysts. The corresponding reduction products (NH_3 and NO_2^-) at different electrolysis time were detected and marked. **d−f**, in 0.1 M KOH and 0.01 M NO_2^- ions using CuSP (**d**), CoS (**e**)P, and CuCoSP (**f**) as catalysts. The generated NH_3 at different electrolysis time was detected and marked.

As shown in (**a**), CuSP gradually deactivated during the NO_3^- electrolysis, as supported by the chronoamperometry curves in supplementary **Fig. S11**. In contrast, as shown in (**d**), CuSP completely lost its activity for NO_2^- reduction in 30 min. This finding indicates the dominant impact of NO_2^-−reducing intermediates (e.g., *NO) on the poisoning of CuSP, rather than that of the potential−dependent adsorbed hydrogen[2], due to the same potential applied for NO_3^- and NO_2^- electrolysis.

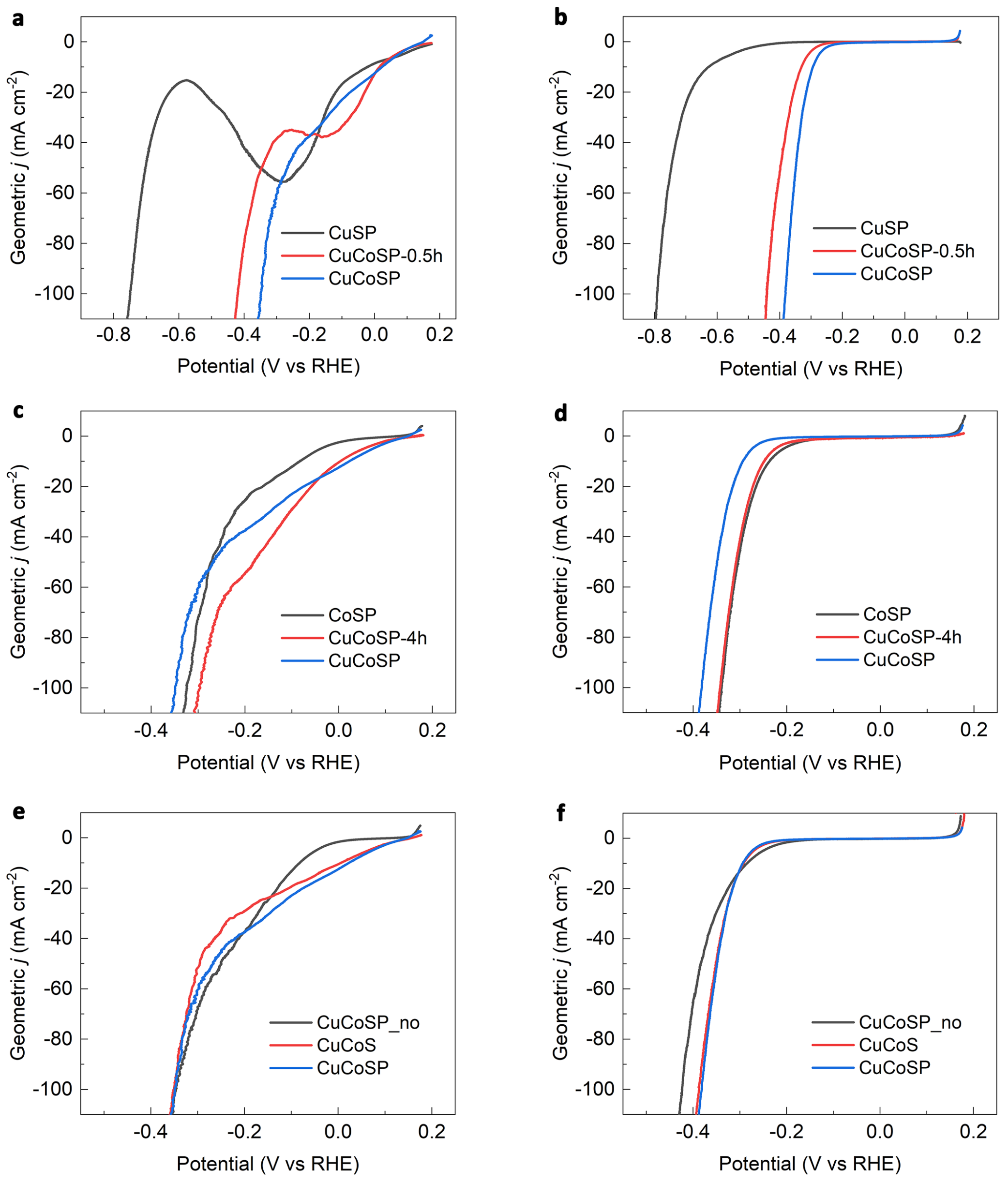

Supplementary Fig. 22 | Comparison of the LSVs of the control samples and CuCoSP in 0.1 M KOH with and without 0.01 M NO_3^-. a, **c**, **e**, Typical LSVs of the catalysts in 0.1 M KOH and 0.01 M NO_3^- and **b**, **d**, **f**, the corresponding LSV curves recorded in 0.1 M KOH. The LSVs were recorded at a scan rate of 5 mV s^{-1}.

In (**a**) and (**b**), the CuCoSP−0.5h catalyst with a lower loading of Co−based phases on Cu foil substrate partly retains the catalytic features of CuSP for nitrate reduction but shows a remarkable enhancement of H_2−evolution reduction (HER) activity. Comparatively, in the presence of 0.01 M NO_3^- (**c**), the CuCoSP−4h catalyst with a higher loading of Co−based phases shows a more negative potential than CuCoSP in the low−current region and much more positive potential in the high−current region. The former indicates that the high loading of Co−based phases blocks the active sites of Cu−based phases, while the latter corresponds to the improved HER activities of CuCoSP−4h, which are close to those of CoSP, as shown in (**d**). In (**e**) and (**f**), the CuCoSP_no catalyst shows a poorer activity for NO_3^- reduction compared to CuCoSP, suggesting that the anionic ligands (S_2^- and PO_4^{3-}) did not play a dominant role in the NO_3^- reduction processes. In (**e**), although the CuCoS shows a little lower activity than the CuCoSP, the CuCoS and CuCoSP have almost the same selectivity and FE for NH_3 production, as shown in supplementary **Fig. S11** and **Fig. S19.** Thus, the difference between CuCoS and CuCoSP in their activity should be from their different morphology. The nanorod morphology of CuCoSP contributes to achieving a higher activity for nitrate reduction.

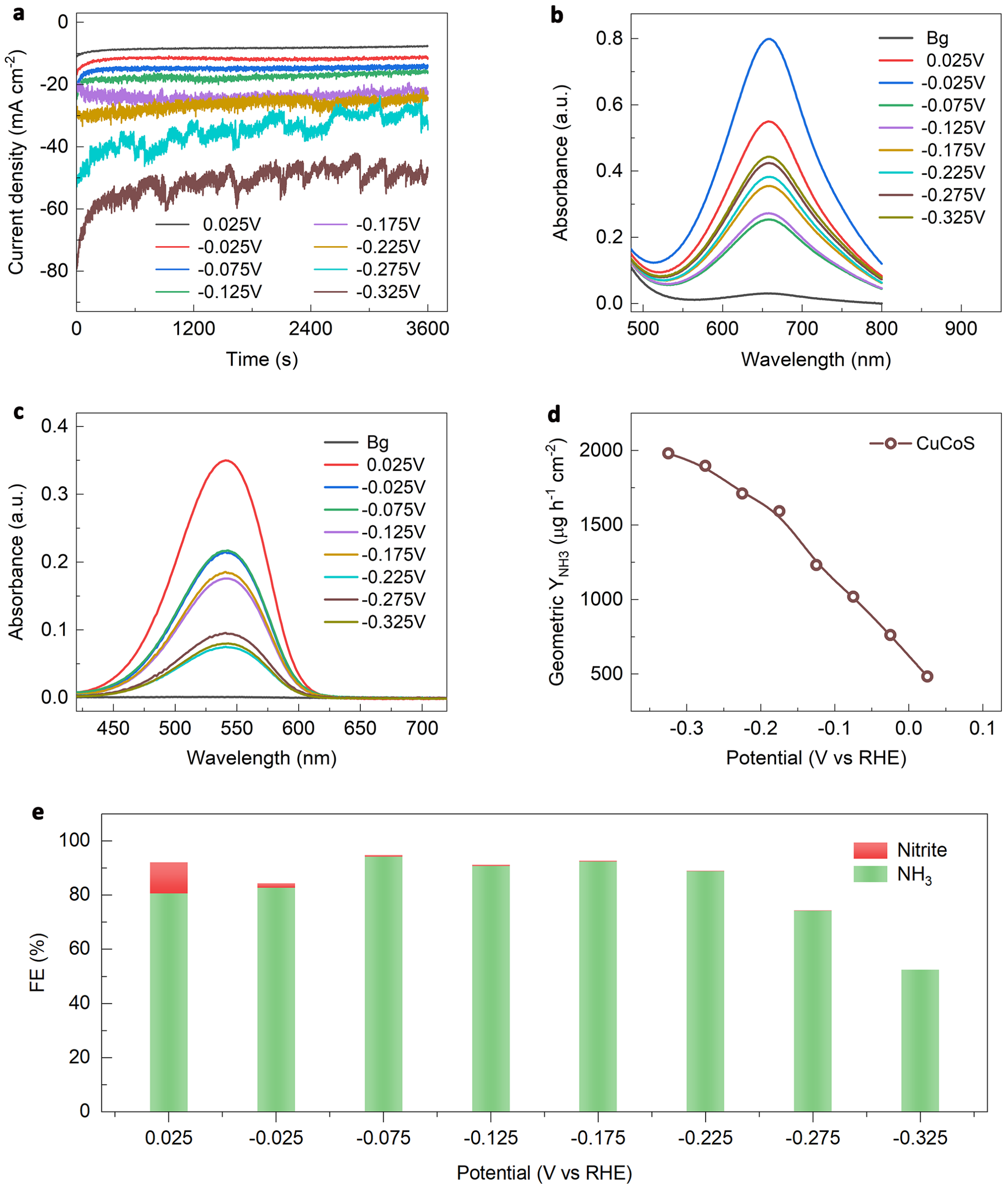

Supplementary Fig. S23 | NH_3 synthesis performance of CuCoS at a series of potentials. a, Chronoamperometry measurements at different potentials for 1 h in 0.1 M KOH and 0.01 M NO_3^-. **b**, **c**, UV–vis absorption spectra of NH_3 (**b**) and NO_2^- (**c**) measurements. Note that to detect the NH_4^+, the post–electrolysis electrolytes at 0.025 V and −0.025 V were diluted 6 times, while the electrolytes obtained at the other electrolysis potentials were diluted 30 times. As for the NO_2^- tests, the electrolytes obtained at all the electrolysis potentials were diluted 5 times. Background (Bg) signals were recorded using 0.1 M KOH. **d**, Y_{NH3} of CuCoS defined by the geometric area of electrodes. **e**, FE of NH_3 and NO_2^-.

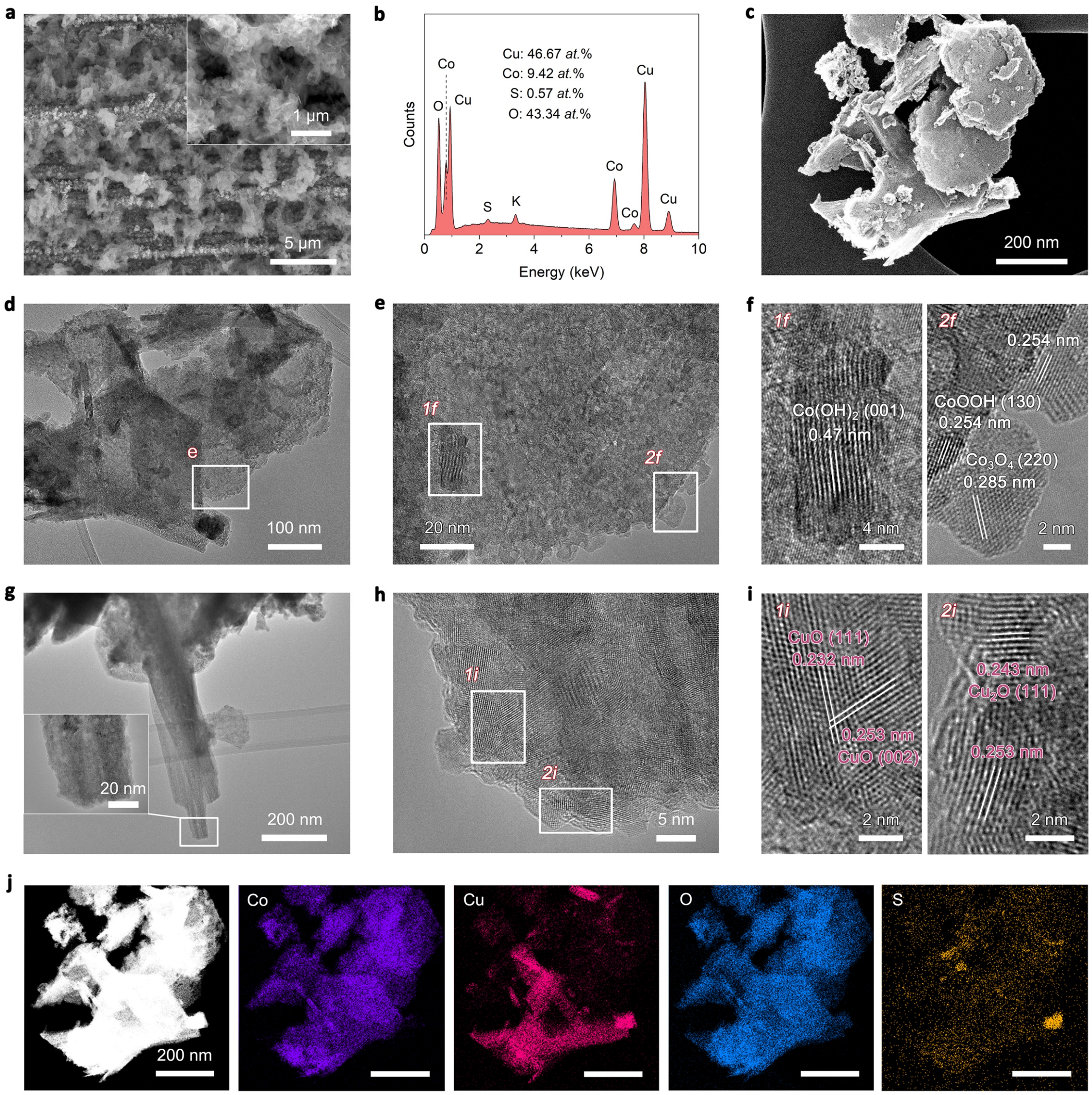

Supplementary Fig. S24 | Characterizations of CuCoSP catalysts after repeating three electrolysis of one hour at −0.325 V (*vs.* RHE) in 0.1 M KOH and 0.01 M NO_3^-. a, **b**, A typical SEM image (**a**) and corresponding EDS patterns (**b**). **c**, **d**, A typical high−resolution SEM image (**c**) and corresponding TEM image (**d**). **e**, **f**, High−resolution TEM images of the marked region in (**d**) and (**e**). **g–i**, Typical TEM and HR−TEM images of the nanorod morphology. **j**, The corresponding high−angle annular dark−field (HAADF) TEM and EDS elemental mapping images.

The high−resolution SEM images, TEM images, and EDX mapping reveal that the Co−based phases are mainly distributed on the hexagon−shaped nanosheets, while the Cu−based phases mainly exist in the form of nanorods. This result suggests that continuous electrolysis indeed promotes the separation of Cu−based phases and Co−based phases in CuCoSP catalysts, rather than the formation of a bulk Cu−Co alloy catalyst (even if the electrolysis potential is at −0.325V). This phase−separation effect contributes to the formation of a tandem catalysis system.

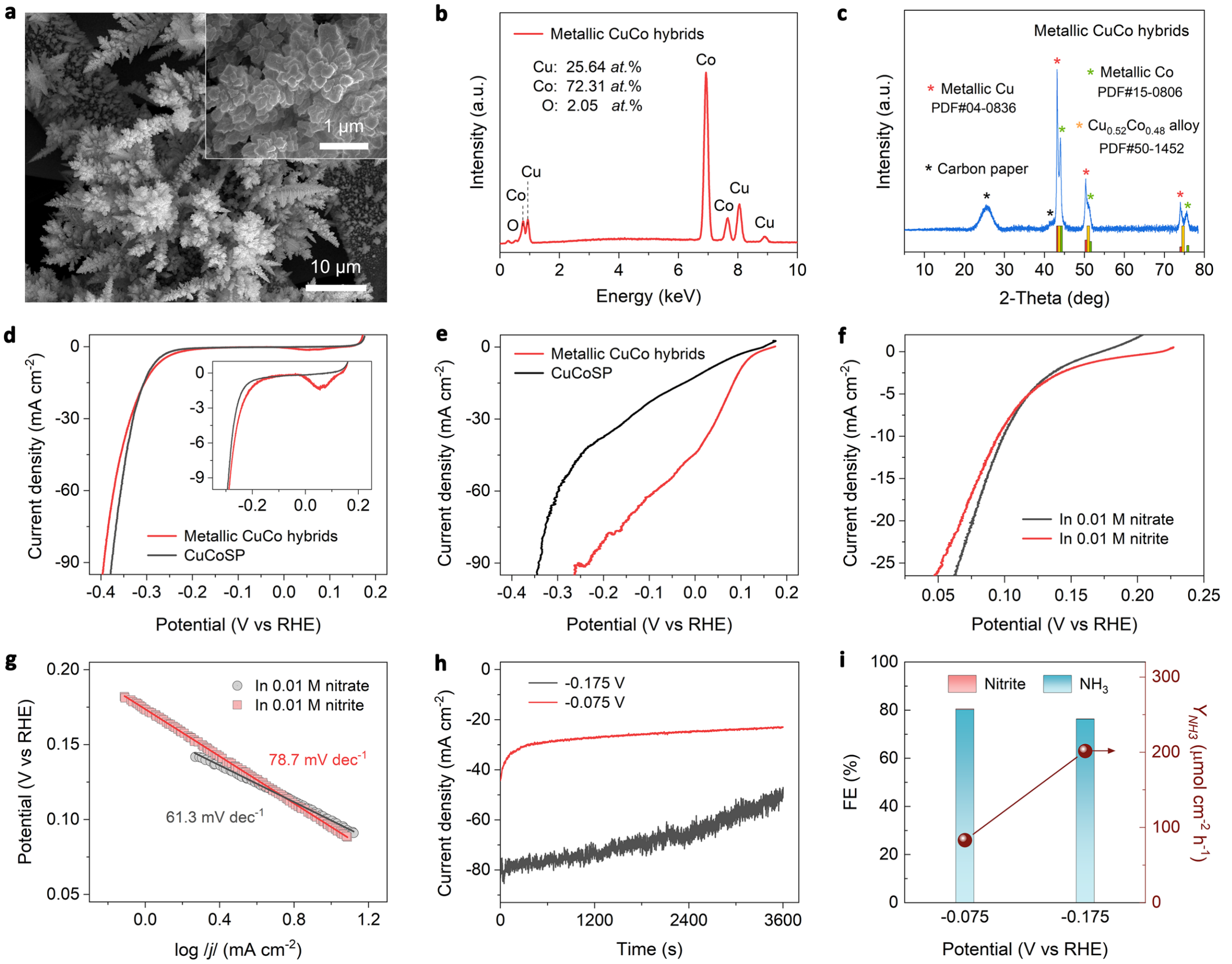

Supplementary Fig. S25 | Characterizations and NO3RR activity of metallic CuCo hybrid catalysts. a, SEM images of CuCo hybrids. **b**, EDX patterns. **c**, XRD patterns. **d**, **e**, LSV curves of CuCo hybrids in 0.1 M KOH (**d**) and 0.1 M KOH containing 0.01 M NO_3^- (**e**), which were recorded at a scan rate of 5 mV s^{-1}. **f**, **g**, LSV curves recorded at a scan rate of 1 mV s^{-1} (**f**) and the derived Tafel slopes (**g**). **h**, **i**, Chronoamperometry curves (**h**) and the corresponding FE and Y_{NH3} at the tested potentials(**i**).

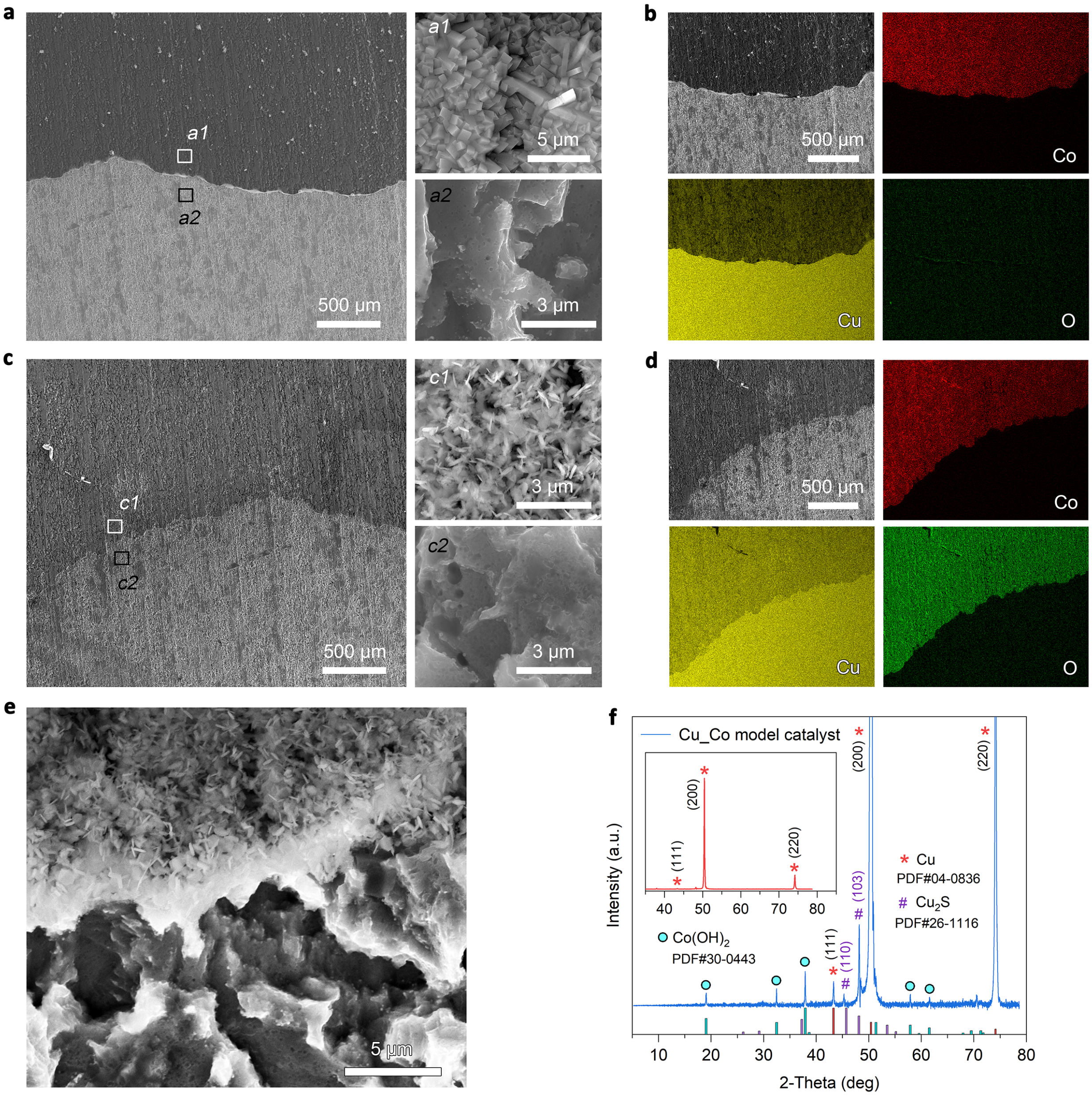

Supplementary Fig. S26 | Characterizations of Cu_Co(OH)$_2$ model catalysts. a, Typical SEM images of a Cu_ZIF(Co) precursor. **b**, EDX mapping of Cu, Co, and O in the Cu_ZIF(Co) precursor. **c**, Typical SEM images of the Cu_Co(OH)$_2$ model catalyst. **d**, EDX mapping of Cu, Co, and O in the Cu_Co(OH)$_2$ model catalyst. **e**, Typical SEM image at the border between the Cu and Co(OH)$_2$ layers. **f**, XRD patterns of the Cu_Co(OH)$_2$ model catalyst.

SEM images, EDX mapping and XRD patterns confirm that the Cu_Co(OH)$_2$ model catalyst consists of an isolated Cu layer and a Co(OH)$_2$ layer, as well as a well–defined border between the two layers, which can be used to simulate the roles of CuSP, CoSP, and CuCoSP, respectively. Note that a porous and rough Cu foil (prepared by HCl–etching CuSP as described in the experimental section) was used as the substrate for growth of ZIF(Co) MOF precursors and the preparation of Cu_Co(OH)$_2$ model catalyst, which is mainly due to the fast deactivation of the smooth Cu foil during the NO_3RR as shown in supplementary **Fig. S14**. Thus, a porous and rough Cu substrate with relatively higher activity and stability for NO_3RR was obtained which is more suitable for performing the subsequent SECM experiments and recording the activity map of NO_2^- and NH_3 formation.

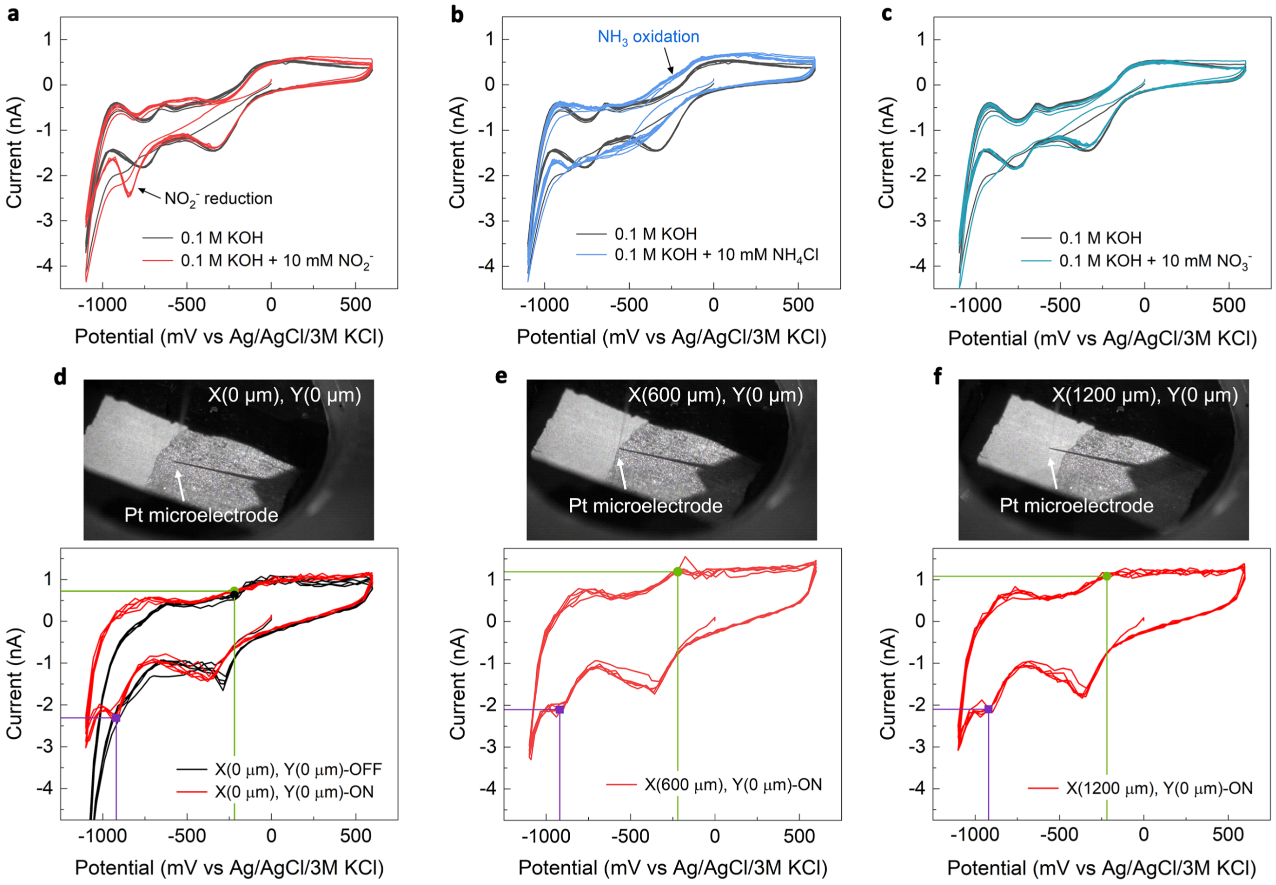

Supplementary Fig. S27 | Electrochemical response of a Pt−ultramicroelectrode (Pt−UME). a, CVs of Pt−UME in Ar−saturated 0.1 M KOH in presence and absence of 10 mM NO_2^-. **b**, CVs of Pt−UME in Ar−saturated 0.1 M KOH in presence and absence of 10 mM NH_4Cl. **c**, CVs of Pt−UME in Ar−saturated 0.1 M KOH in presence and absence of 10 mM NO_3^-. **d−f**, Typical CVs of a Pt−UME recorded in the shear−force interaction region, where the Pt−UME is positioned <500 nm away from the surface of the Cu_Co(OH)$_2$ catalyst. When a potential of −0.12 V (vs. RHE) is applied at the Cu_Co(OH)$_2$ catalyst in 50 mM NO_3^- at pH 13, the situation is marked 'ON'; when no potential is applied, it is marked 'OFF'.

To map the local in−situ generated concentration of NO_2^- and NH_3 above the surface of the Cu_Co(OH)$_2$ catalyst (polarized at −0.12 V), the Pt−UME scans from the side of Cu layer to the side of Co(OH)$_2$ layer. The initial position of the Pt−UME above the Cu layer is marked as X (0 μm), Y (0 μm); the position of the border between the Cu layer and Co(OH)$_2$ layer is at about X (600 μm), Y (0 μm); the final position above the Co(OH)$_2$ layer is marked as X (1200 μm), Y (0 μm). The black point, green points, and purple points marked in (**d**−**f**) correspond to the selected potentials for identifying NH_3 oxidation (at 0.76 V vs RHE) and NO_2^- reduction (at 0.06 V vs. RHE). The current at the corresponding potentials is used to derive the local concentrations of NH_3 and NO_2^- generated on the Cu_Co(OH)$_2$ catalyst polarized at −0.12 V (vs RHE) in 50 mM NO_3^- at pH 13. Note that the profile of CVs of the Pt−UME in the shear−force interaction region (**d**−**f**) is slightly different from those in the bulk of the electrolyte solution (**a**−**c**). This should be attributed to the impact of the microenvironment above the surface, e.g. the local pH value, adsorption of N−containing intermediates, or mass transport limitations, in the shear−force interaction region (<500 nm away from the surface of Cu_Co(OH)$_2$). However, this does not compromise the identification of the NO_2^- reduction and NH_3 oxidation signals.

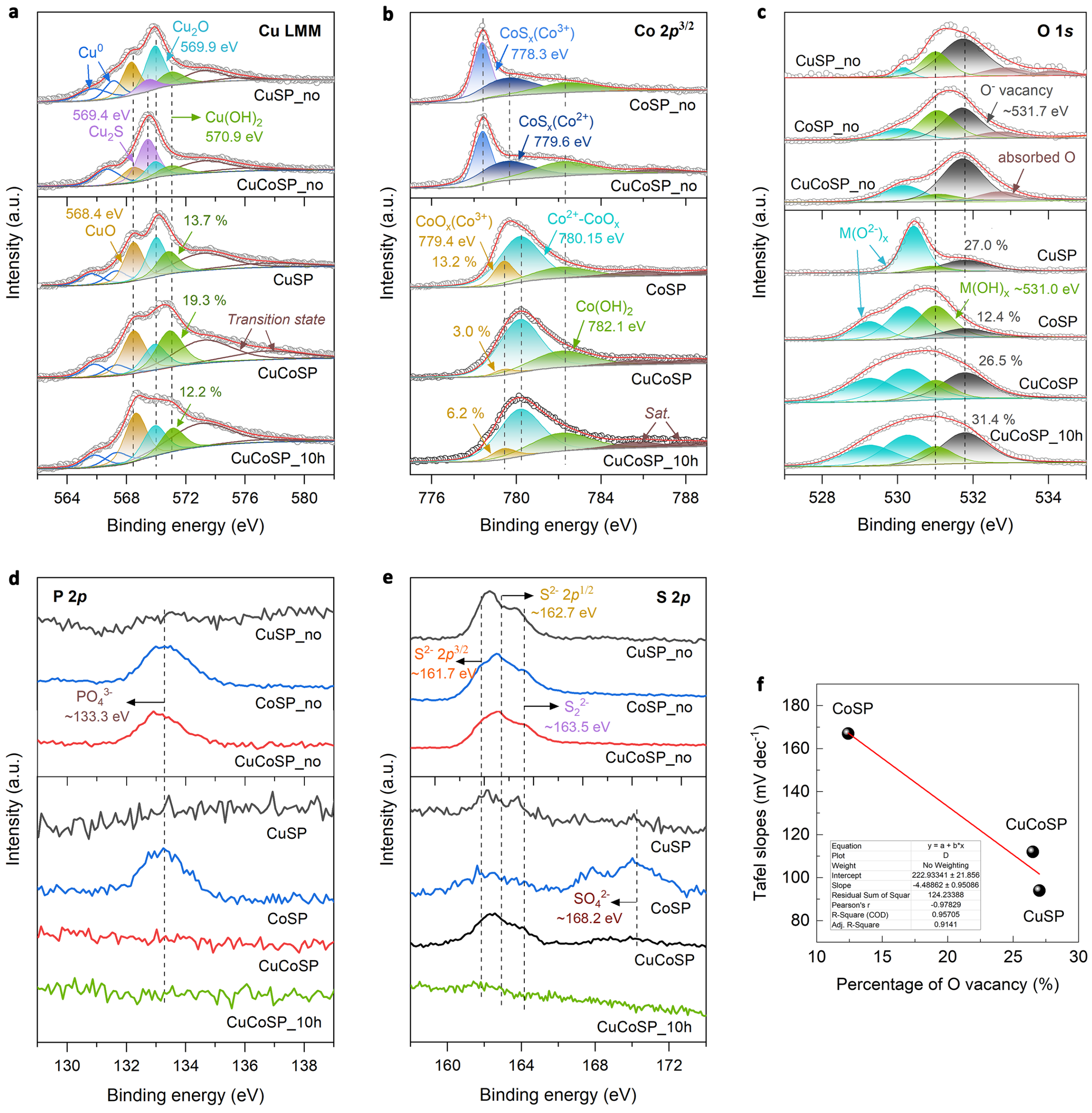

Supplementary Fig. S28 | XPS spectra analysis of CuSP_no, CuSP, CoSP_no, CoSP, CuCoSP_no, CuCoSP and CuCoSP_10h (CuCoSP after stability tests for 10 h). a, Cu LMM spectra and the fitted peaks. **b**, Co $2p^{3/2}$ core−level spectra and the fitted peaks. **c**, O 1*s* core−level XPS spectra and the fitted peaks. **d**, P 2*p* core−level spectra. **e**, S 2*p* core−level spectra. **f**, Correlation of the Tafel slopes and the percentage of O vacancy for CoSP, CuCoSP, and CuSP. The results suggest that the initial Cu and Co metal sulfides (in CuSP_no, CoSP_no and CuCoSP_no) were heavily oxidized and converted into corresponding Co/Cu−based oxides and hydroxides (in CuSP, CoSP and CuCoSP) after the electrochemical redox activation.

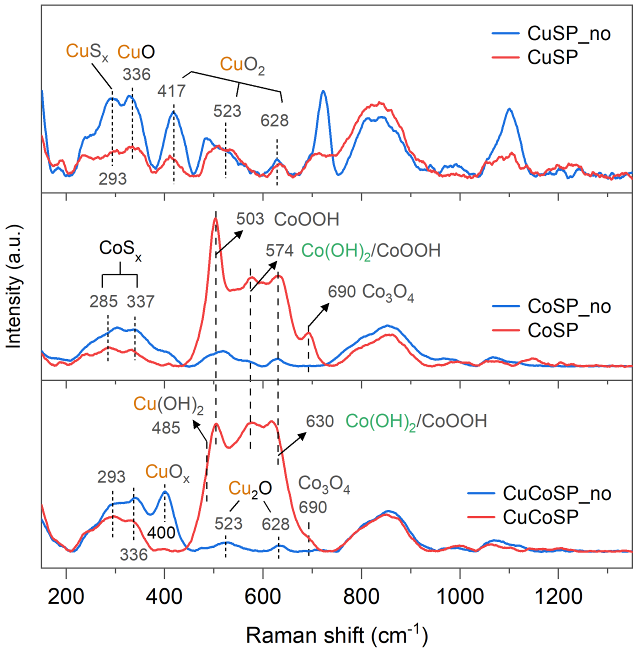

Supplementary Fig. S29 | Ex–situ Raman analysis. Ex–situ Raman spectra of CuSP_no, CoSP_no and CuCoSP_no, as well as their corresponding products (CuSP, CoSP and CuCoSP) after the electrochemical redox activation.

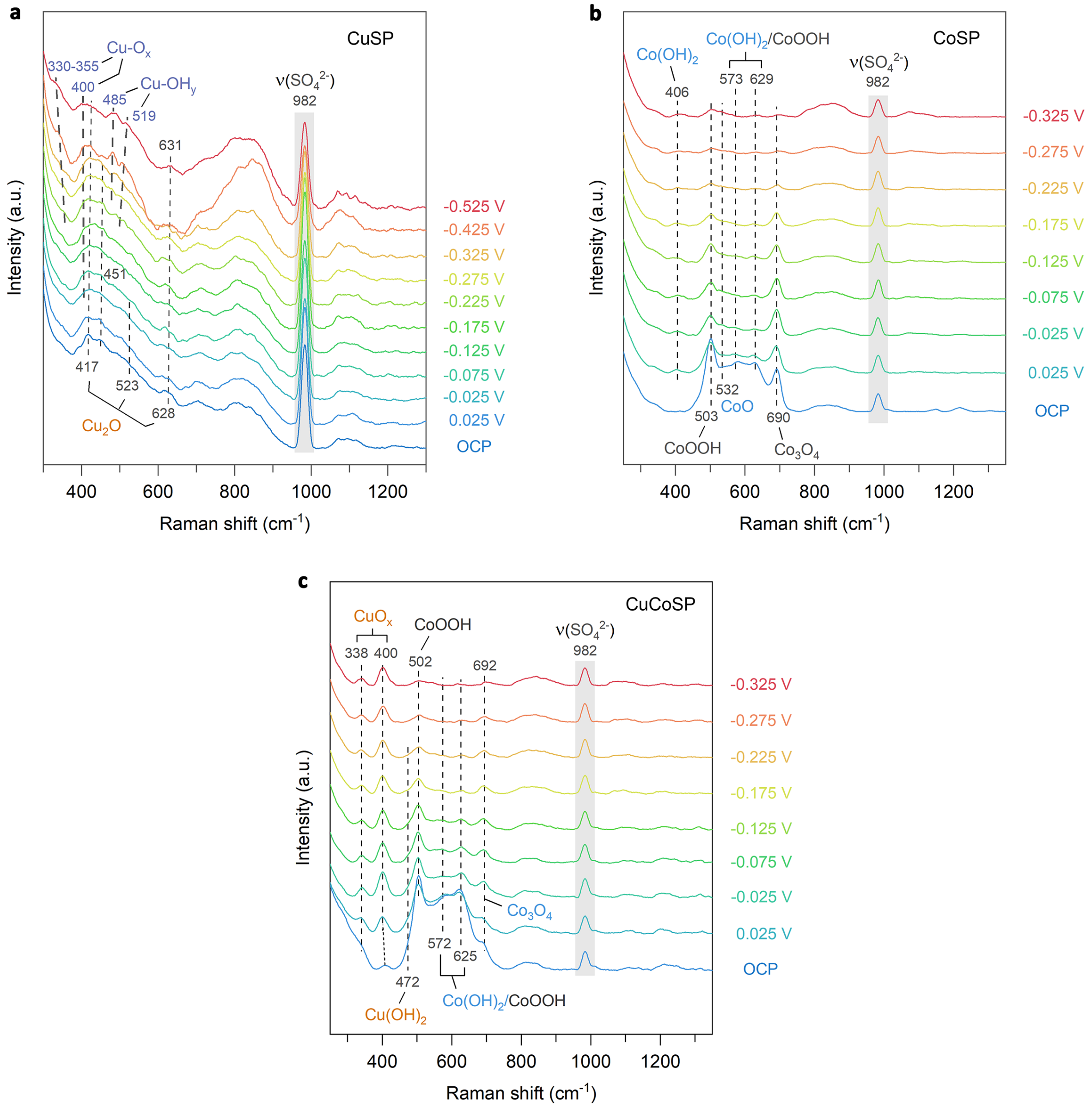

Supplementary Fig. S30 | In–situ Raman spectra of the catalysts in 0.01 M KOH and 0.45 M K_2SO_4 at a series of applied potentials. **a**, CuSP. **b**, CoSP. **c**, CuCoSP. The Raman spectra were recorded after applying a constant potential for 10 min.

Supplementary Notes

Supplementary Note 1 | Assignment of XPS peaks. Cu LMM XPS spectra (supplementary **Fig. S28a)** show the presence of multiple Cu–based phases, including Cu^0 (~567.3 eV) and (~565.5 eV), CuO (568.4 eV), Cu_2S (569.4 eV), Cu_2O (569.9 eV), $Cu(OH)_2$ (570.9 eV), and transition state peaks of LMM spectra (~573.3 eV) and ~577.5 eV) on the surface of CuSP_no and CuCoSP_no[3–5], due to the surface oxidation of the two samples in Air. After redox activation, the signal of surface Cu_2S phase disappeared, accompanied by the increase of the signals associated with Cu–based oxide and hydroxide phases. Compared to CuSP, the CuCoSP catalyst shows a ~1.4 times higher content of $Cu(OH)_2$ phase, implying that the relative ratio of Cu–based phases in CuCoSP is probably impacted by the coexistent Co–based phases. Remarkably, metallic Cu^0 exists in both CuSP and CuCoSP after the redox activation, contributing to the in–situ formation of a Cu/CuO_x active phase for NO_3^-–to–NO_2^- conversion at a series of applied potentials.

Co $2p^{3/2}$ XPS spectra in supplementary **Fig. S28b** show features characteristic of Co^{3+} (778.3 eV) and Co^{2+} (779.6 eV) of CoS_x phase and a $Co(OH)_2$ phase (782.1 eV) in both CoSP_no and CuCoSP_no[6,7]. This result, togethering with that shown in supplementary **Fig. S28a**, suggest that the initial CuCoSP_no precursor is mainly composed of Cu–Co binary metal sulfides, as further supported by the EDX spectra in supplementary **Fig. S1** and HR–TEM images and SAED patterns in **Fig. 1,** supplementary **Fig. S3** and **Fig. S4**. After redox activation, the CoS_x phases in CoSP_no and CuCoSP_no precursors were heavily oxidized and converted to Co–based oxide (Co^{3+} of CoO_x at 779.4 eV and Co^{2+} of CoO_x at 780.15 eV) and hydroxide (782.1 eV) phase[7–10]. Remarkably, the content of Co^{3+}–based phase in CuCoSP is ~4–fold lower than that in CoSP. Considering that the Co^{2+}–based phases are identified as the active phase for NO_2^- reduction to NH_3, the lower content of Co^{3+}–bases phases in CuCoSP indicates that the active Co^{2+}–based phases in CuCoSP might be stabilized by the coexistent Cu–bases phases, which is critical for the observed high activity and selectivity of CuCoSP catalysts for tandem nitrate reduction.

According to the previous reports[11], as shown in O 1*s* spectra (supplementary **Fig. S28c**), the oxygen species at a binding energy lower than 530.6 eV is assigned to the O^{2-} in metal oxides, while the XPS peak at ~531 eV is from metal hydroxides. The XPS peak at ~531.7 eV is related to the O vacancies[12,13], which allows compensation for some deficiencies in the subsurface of transition metal oxides. This oxygen vacancy species was previously described as "O^-" species due to its high covalence bonding to the transition metal.

In P 2*p* spectra (supplementary **Fig. S28d**), the XPS peak at ~133.3 eV is from phosphate (PO_4^{3-}) ions[14]. In particular, there is no detectable PO_4^{3-} ions on CuSP and CuCoSP, suggesting that the PO_4^{3-} ligands in CuSP_no and CuCoSP_no was desorbed after the redox activation. In supplementary **Fig. S28e**, the S 2*p* spectra reveal the presence of S^{2-} (S^{2-} $2p^{3/2}$ at ~161.7 eV and S^{2-} $2p^{1/2}$ at ~162.7 eV) and S_2^{2-} species (~163.5 eV) in CuSP_no, CoSP_no, and CuCoSP_no[6,7]. The signals of these S species are sharply attenuated after the electrochemical redox activation, concomitant with an emergence of SO_4^{2-} signals (at ~168.2 eV)[7].

Supplementary Note 2 | Assignment of Raman peaks. According to the previous reports, the Raman spectra of the synthesized samples (supplementary **Fig. S29**) were carefully identified. As for CuSP_no, the Raman peak at 293 cm^{-1} is assigned to the CuS_x phase[6], while that at 336 cm^{-1} is related to CuO[15,16]. A set of Raman peaks at 417, 523 and 628 cm^{-1} is a typical feature of Cu_2O[15,16].

As for CoSP_no and CoSP, the Raman peaks at ~285 and ~337 cm^{-1} were previously assigned to the E_g and T_g modes of CoS_x[6,17,18], while the Raman peaks at 503 cm^{-1} and 690 cm^{-1} correspond to the strongest Raman signals of CoOOH and Co_3O_4, respectively[8]. Besides, both CoOOH and $Co(OH)_2$ show characteristic board peaks at ~574 cm^{-1} and ~630 cm^{-1} [19–21]. The Raman peak of CoO at ~530 cm^{-1} could not be directly identified in the Raman spectra of CoSP_no and CoSP due to the overlap of the peaks[22].

The CuCoSP_no basically shows the Raman features of both CuSP_no and CoSP_no. A new Raman peak emerges at ~400 cm^{-1} on CuCoSP_no, which exists between the Raman signals of CuO at 336 cm^{-1} and Cu_2O at 417 cm^{-1}. Although the Raman signal at 417 cm^{-1} associated with Cu_2O disappears on CuCoSP_no, the other two signals at 523 and 628 cm^{-1} are retained. Thus, we assign the signal of 400 cm^{-1} to a mixed phase of CuO and Cu_2O (CuO_x).

The signals associated with CuS_x and CoS_x phases are sharply attenuated in the CuSP, CoSP and CuCoSP catalysts, which further demonstrate the conversion of metal sulfide phases (in CuSP_no, CoSP_no and CuCoSP_no) into corresponding metal oxide and hydroxide phases after the electrochemical redox activation.

Supplementary Tables

Supplementary Table S1 | Comparison of the NH_3 synthesis activity of the CuCoSP catalyst with other catalysts reported to date using nitrate as the nitrogen source under ambient conditions. Error denotes the standard deviations of yield rate and Faradaic efficiency calculation from three independent samples.

Catalysts	Electrolyte	Potential (V vs. RHE)	NH_3 yield rate	Faradaic Efficiency (%)	Reference
**CuSP** on *Cu foil*	0.1 M KOH and 0.01 M NO_3^-	−0.175	540.7 ± 52.1 μg h^{-1} cm^{-2}	55.7 ± 2.4	This work
**CoSP** on *carbon paper*	0.1 M KOH and 0.01 M NO_3^-	−0.175	1713.0 ± 225.1 μg h^{-1} cm^{-2}	73.2 ±3.5	This work
**CuCoSP** on *Cu foil*	0.1 M KOH and 0.01 M NO_3^-	−0.175	2576.6 ± 8.4 μg h^{-1} cm^{-2}	92.8 ± 1.7	This work
	0.1 M KOH and 0.1 M NO_3^-	−0.175	1.17 mmol h^{-1} cm^{-2} or 250.41 mA cm^{-2}	90.6	This work
**CuCoSP** on *Cu foil (average values of 10 h stability tests)*	0.1 M KOH and 0.01 M NO_3^-	−0.175	2642.9 ± 104.7 μg h^{-1} cm^{-2}	94.2 ± 1.7	This work
PTCDA/O−**Cu** on *carbon cloth*	0.1 M PBS and 500 ppm NO_3^-	−0.4	436 ± 85 μg h^{-1} cm^{-2}	77 ± 3	H. Wang et al.[23]
**Cu/Cu$_2$**O NWAs *on Cu mesh*	0.5 M Na_2SO_4 and 200 ppm NO_3^-	−0.85	0.2449 mmol h^{-1} cm^{-2}	81	B. Zhang et al.[24]
**CuNi** alloy on *Cu foam*	1 M KOH and 0.1 M NO_3^-	−0.1	NH_3 partial current density 90 mA cm^{-2}	~95	Edward H. Sargent et al.[25]
**Co/Co**O NSAS on *Ni foam*	0.1 M Na_2SO_4 and 200 ppm NO_3^-	−1.3 V *vs.* SCE (~−0.64 V *vs.* RHE)	194.46 μmol h^{-1} cm^{-2}	~93.8	B Zhang et al.[26]
**Co$_3$**O$_4$/Ti	0.1 M Na_2SO_4 and 100 ppm NO_3^-	10 mA cm^{-2}	NH_3 partial current density 6.5 mA cm^{-2}	65	J. Jia et al.[27]
Strained **Ru** nanoclusters on *carbon paper*	1 M KOH and 1 M NO_3^-	−0.2	1.17 mmol h^{-1} cm^{-2}	~100	L. Zhang et al.[28]
**Ti**O$_2$ nanotubes with oxygen vacancies on *Ti foil*	0.5 M Na_2SO_4 and 50−200 ppm NO_3^-	−1.3 V *vs.* SCE (~−0.94 V *vs.* RHE)	0.045 mmol h^{-1} mg^{-1}	95.2	B. Zhang et al.[29]
**Ti** foil	0.4 M NO3− at pH = ~0.77	−1.0	NH_3 partial current density 22 mA cm^{-2}	82	W. Ma et al.[30]
**NiAlMnCoCu** alloy on *Ni foam*	0.5 M KOH and 0.25 M NO_3^-	−1.2 *vs.* SCE (~−0.15 V *vs.* RHE)	----------	89.4	Q. Liu et al.[31]

Supplementary Table S2 | Reaction kinetic parameters of the CuCoSP catalyst and controls for NO_3^- and NO_2^- reduction under ambient conditions. Error denotes the standard deviations of reaction constant calculation from three sampling time (15 min, 30 min and 60 min), as shown in supplementary **Fig. 20**.

Catalysts	Electrolytes	Potential @ −1 mA cm⁻² (mV vs. RHE)	Reaction constant (k) (min⁻¹) at −0.175 V (vs. RHE)	Tafel slopes (mV dec⁻¹)	j_{NH3} (mA cm⁻²) at −0.175 V (vs. RHE)
**CuSP**	0.1 M KOH + 0.01 M NO_3^-	105.24	$K_1 = (8.6 \pm 3.84) \times 10^{-3}$	94	−7.74
	0.1 M KOH + 0.01 M **NO_2^-**	−74.75	$K_2 = (4.01 \pm 2.35) \times 10^{-3}$	72	--------
**CoSP**	0.1 M KOH + 0.01 M NO_3^-	−70.95	$K_1 = (3.87 \pm 0.25) \times 10^{-3}$	167	−19.95
	0.1 M KOH + 0.01 M **NO_2^-**	176.24	$K_2 = (12.19 \pm 1.03) \times 10^{-3}$	106	--------
**CuCoSP**	0.1 M KOH + 0.01 M NO_3^-	111.44	$K_1 = (5.97 \pm 0.95) \times 10^{-3}$	112	−33.15
	0.1 M KOH + 0.01 M **NO_2^-**	158.24	$K_2 = (10.63 \pm 1.78) \times 10^{-3}$	101	---------

During the NO_3^- electroreduction over CoSP and CuCoSP, the local surface NO_2^- concentration formed on CoSP and CuCoSP can be apparently reflected by their k_1 values. A larger k_1 value means a higher reduction rate of NO_3^- to NO_2^-, thus corresponding to a higher local surface NO_2^- concentration. As such, the relative ratio of the local surface NO_2^- concentration on CoSP and on CuCoSP can be estimated as: k_1 (CoSP): k_1 (CuCoSP) = 5.97: 3.87 = 1.54: 1.

References

1. Su, J. F., Ruzybayev, I., Shah, I. & Huang, C. P. The electrochemical reduction of nitrate over micro−architectured metal electrodes with stainless steel scaffold. *Appl. Catal. B: Environ.* **180**, 199−209 (2016).
2. Pérez−Gallent, E., Figueiredo, M. C., Katsounaros, I. & Koper, M. T. M. Electrocatalytic reduction of Nitrate on Copper single crystals in acidic and alkaline solutions. *Electrochim. Acta* **227**, 77−84 (2017).
3. Krylova, V. & Andrulevičius, M. Optical, XPS and XRD Studies of Semiconducting Copper Sulfide Layers on a Polyamide Film. *Int. J. Photoenergy* **2009**, 304308 (2009).
4. Platzman, I., Saguy, C., Brener, R., Tannenbaum, R. & Haick, H. Formation of Ultrasmooth and Highly Stable Copper Surfaces through Annealing and Self−Assembly of Organic Monolayers. *Langmuir* **26**, 191−201 (2010).
5. Platzman, I., Brener, R., Haick, H. & Tannenbaum, R. Oxidation of Polycrystalline Copper Thin Films at Ambient Conditions. *J. Phys. Chem. C* **112**, 1101−1108 (2008).
6. He, W., Liberman, I., Rozenberg, I., Ifraemov, R. & Hod, I. Electrochemically Driven Cation Exchange Enables the Rational Design of Active CO_2 Reduction Electrocatalysts. *Angew. Chem. Int. Ed.* **59**, 8262−8269 (2020).
7. He, W., Ifraemov, R., Raslin, A. & Hod, I. Room−Temperature Electrochemical Conversion of Metal–Organic Frameworks into Porous Amorphous Metal Sulfides with Tailored Composition and Hydrogen Evolution Activity. *Adv. Funct. Mater.* **28**, 1707244 (2018).
8. Yang, J., Liu, H., Martens, W. N. & Frost, R. L. Synthesis and Characterization of Cobalt Hydroxide, Cobalt Oxyhydroxide, and Cobalt Oxide Nanodiscs. *J. Phys. Chem. C* **114**, 111−119 (2010).
9. McIntyre, N. S. & Cook, M. G. X−ray photoelectron studies on some oxides and hydroxides of cobalt, nickel, and copper. *Anal. Chem.* **47**, 2208−2213 (1975).
10. Dou, S. *et al.* Atomic−Scale CoOx Species in Metal–Organic Frameworks for Oxygen Evolution Reaction. *Adv. Funct. Mater.* **27**, 1702546 (2017).
11. Dupin, J.−C., Gonbeau, D., Vinatier, P. & Levasseur, A. Systematic XPS studies of metal oxides, hydroxides and peroxides. *Phys. Chem. Chem. Phys.* **2**, 1319−1324 (2000).

12. Xu, L. *et al.* Plasma–Engraved Co_3O_4 Nanosheets with Oxygen Vacancies and High Surface Area for the Oxygen Evolution Reaction. *Angew. Chem. Int. Ed.* **55**, 5277–5281 (2016).
13. Wang, Z. *et al.* Identifying Copper Vacancies and Their Role in the CuO Based Photocathode for Water Splitting. *Angew. Chem. Int. Ed.* **58**, 17604–17609 (2019).
14. Cobo, S. *et al.* A Janus cobalt–based catalytic material for electro–splitting of water. *Nat. Mater.* **11**, 802–807 (2012).
15. Deng, Y., Handoko, A. D., Du, Y., Xi, S. & Yeo, B. S. In Situ Raman Spectroscopy of Copper and Copper Oxide Surfaces during Electrochemical Oxygen Evolution Reaction: Identification of Cu^{III} Oxides as Catalytically Active Species. *ACS Catal.* **6**, 2473–2481 (2016).
16. Zhao, Y. *et al.* Speciation of Cu Surfaces During the Electrochemical CO Reduction Reaction. *J. Am. Chem. Soc.* **142**, 9735–9743 (2020).
17. Chen, C.–J. *et al.* An integrated cobalt disulfide (CoS_2) co–catalyst passivation layer on silicon microwires for photoelectrochemical hydrogen evolution. *J. Mater. Chem. A* **3**, 23466–23476 (2015).
18. Givalou, L. *et al.* Electrodeposited cobalt–copper sulfide counter electrodes for highly efficient quantum dot sensitized solar cells. *Electrochim. Acta* **210**, 630–638 (2016).
19. Koza, J. A., Hull, C. M., Liu, Y.–C. & Switzer, J. A. Deposition of β–$Co(OH)_2$ Films by Electrochemical Reduction of Tris(ethylenediamine)cobalt(III) in Alkaline Solution. *Chem. Mater.* **25**, 1922–1926 (2013).
20. Liu, Y.–C., Koza, J. A. & Switzer, J. A. Conversion of electrodeposited $Co(OH)_2$ to CoOOH and Co_3O_4, and comparison of their catalytic activity for the oxygen evolution reaction. *Electrochim. Acta* **140**, 359–365 (2014).
21. Gao, Y. Q., Li, H. B. & Yang, G. W. Amorphous $Co(OH)_2$ nanosheet electrocatalyst and the physical mechanism for its high activity and long–term cycle stability. *J. Appl. Phys.* **119**, 034902 (2016).
22. Rivas–Murias, B. & Salgueiriño, V. Thermodynamic CoO–Co_3O_4 crossover using Raman spectroscopy in magnetic octahedron–shaped nanocrystals. *J. Raman Spectrosc.* **48**, 837–841 (2017).
23. Chen, G.–F. *et al.* Electrochemical reduction of nitrate to ammonia via direct eight–electron transfer using a copper–molecular solid catalyst. *Nat. Energy* **5**, 605–613 (2020).
24. Wang, Y., Zhou, W., Jia, R., Yu, Y. & Zhang, B. Unveiling the Activity Origin of a Copper–based Electrocatalyst for Selective Nitrate Reduction to Ammonia. *Angew. Chem. Int. Ed.* **59**, 5350–5354 (2020).
25. Wang, Y. *et al.* Enhanced Nitrate–to–Ammonia Activity on Copper–Nickel Alloys via Tuning of Intermediate Adsorption. *J. Am. Chem. Soc.* **142**, 5702–5708 (2020).
26. Yu, Y., Wang, C., Yu, Y., Wang, Y. & Zhang, B. Promoting selective electroreduction of nitrates to ammonia over electron–deficient Co modulated by rectifying Schottky contacts. *Sci. China Chem.* **63**, 1469–1476 (2020).
27. Su, L. *et al.* Electrochemical nitrate reduction by using a novel Co_3O_4/Ti cathode. *Water Res.* **120**, 1–11 (2017).
28. Li, J. *et al.* Efficient Ammonia Electrosynthesis from Nitrate on Strained Ruthenium Nanoclusters. *J. Am. Chem. Soc.* **142**, 7036–7046 (2020).
29. Jia, R. *et al.* Boosting Selective Nitrate Electroreduction to Ammonium by Constructing Oxygen Vacancies in TiO_2. *ACS Catal.* **10**, 3533–3540 (2020).
30. McEnaney, J. M. *et al.* Electrolyte Engineering for Efficient Electrochemical Nitrate Reduction to Ammonia on a Titanium Electrode. *ACS Sustain. Chem. Eng.* **8**, 2672–2681 (2020).
31. Lu, C., Lu, S., Qiu, W. & Liu, Q. Electroreduction of nitrate to ammonia in alkaline solutions using hydrogen storage alloy cathodes. *Electrochim. Acta* **44**, 2193–2197 (1999).
