# Supplementary material for: Splicing the active phases of copper/cobalt-based catalysts achieves high-rate tandem electroreduction of nitrate to ammonia
Source: Nat Commun. 2022 Mar 2;13:1129. doi: 10.1038/s41467-022-28728-4 (PMC8891333; doi:10.1038/s41467-022-28728-4)
Supplement: Supplementary file 2 — Description of Additional Supplementary Files [file 41467_2022_28728_MOESM2_ESM.docx]

**Table of Contents**

Supplementary Figures 2

Supplementary Fig. S1 | Morphology and composition of ZIF−Co−R, CuCoSP_no and CuCoSP on Cu foils. 2

Supplementary Fig. S2 | Morphology and composition of CuCoS_no and CuCoS on Cu foils. 3

Supplementary Fig. S3 | Nanostructure and element distribution of CuCoSP_no. 4

Supplementary Fig. S4 | X−ray diffraction (XRD) patterns of the samples. 5

Supplementary Fig. S5 | Nanostructure and element distribution of CuCoSP. 6

Supplementary Fig. S6 | Depth−profiling X−ray photoelectron spectroscopies (XPS) of CuCoSP_no and CuCoSP. 7

Supplementary Fig. S7 | Phase separation in CuCoSP_no after electrochemical redox activation. 8

Supplementary Fig. S8 | Morphology and composition of CuSP_no, CuSP, CoSP_no and CoSP. 9

Supplementary Fig. S9 | Linear sweep voltammograms (LSV) tests of CuSP, CoSP and CuCoSP 10

Supplementary Fig. S10 | NH_3_, NO_2_^−^ and NO_3_^−^ quantification using UV−vis absorption spectroscopy. 12

Supplementary Fig. S11 | NH_3_ synthesis performance of CuSP at a series of potentials 12

Supplementary Fig. S12 | NH_3_ synthesis performance of CoSP at a series of potentials. 13

Supplementary Fig. S13 | NH_3_ synthesis performance of CuCoSP at a series of potentials. 14

Supplementary Fig. S14 | Activity of pure Cu foil and carbon paper for the NO_3_RR. 15

Supplementary Fig. S15 | Electrocatalytic tests of CuCoSP in different concentrations of NO_3_^−^. 16

Supplementary Fig. S16 | ^15^NH_4_^+^ detection and ^14^NH_4_^+^ quantification by ^1^H NMR spectra. 17

Supplementary Fig. S17 | Cyclic voltammograms (CV) for the determination of the double−layer capacitance of different samples in Ar−saturated 0.1 M KOH. 18

Supplementary Fig. S18 | Stability tests of CuCoSP for ammonia synthesis carried out via repeating one−hour electrolysis for ten times at −0.175 V (vs. RHE). 19

Supplementary Fig. S19 | Ammonia volatilization in the 0.1 M KOH (pH 13) with time. 19

Supplementary Fig. S20 | LSVs of the catalysts recorded at a scan rate of 1 mV s^−1^ 20

Supplementary Fig. S21 | Concentration−evolving profiles of NO_3_^−^ and NO_2_^−^ ions at −0.175 V vs. RHE. 20

Supplementary Fig. 22 | Comparison of the LSVs of the control samples and CuCoSP in 0.1 M KOH with and without 0.01 M NO_3_^−^. 21

Supplementary Fig. S23 | NH_3_ synthesis performance of CuCoS at a series of potentials. 22

Supplementary Fig. S24 | Characterizations of CuCoSP catalysts after repeating three electrolysis of one hour at −0.325 V (vs. RHE) in 0.1 M KOH and 0.01 M NO_3_^−^. 23

Supplementary Fig. S25 | Characterizations and NO_3_RR activity of metallic CuCo hybrid catalysts. 24

Supplementary Fig. S26 | Characterizations of Cu_Co(OH)_2_ model catalysts. 25

Supplementary Fig. S27 | Electrochemical response of a Pt−ultramicroelectrode (Pt−UME). 26

Supplementary Fig. S28 | XPS spectra analysis of CuSP_no, CuSP, CoSP_no, CoSP, CuCoSP_no, CuCoSP and CuCoSP_10h (CuCoSP after stability tests for 10 h). 27

Supplementary Fig. S29 | Ex−situ Raman analysis. 28

Supplementary Fig. S30 | In−situ Raman spectra of the catalysts in 0.01 M KOH and 0.45 M K_2_SO_4_ at a series of applied potentials. 29

Supplementary Notes 30

Supplementary Note 1 | Assignment of XPS peaks. 30

Supplementary Note 2 | Assignment of Raman peaks. 31

Supplementary Tables 32

Supplementary Table S1 | Comparison of the NH_3_ synthesis activity of the CuCoSP catalyst with other catalysts reported to date using nitrate as the nitrogen source under ambient conditions. 32

Supplementary Table S2 | Reaction kinetic parameters of the CuCoSP catalyst and controls for NO_3_^−^ and NO_2_^−^ reduction under ambient conditions. 33

References 33
